# Supplementary material for: Symbolic metaprogram search improves learning efficiency and explains rule learning in humans
Source: Nat Commun. 2024 Aug 10;15:6847. doi: 10.1038/s41467-024-50966-x (PMC11316799; doi:10.1038/s41467-024-50966-x)
Supplement: Supplementary file 1 — Supplementary Information [file 41467_2024_50966_MOESM1_ESM.pdf]

Supplementary Methods

Type	Description
t1, t2,...	Universally quantified type variables
Int	Integer values
Bool	Boolean values
[X]	List of type X (e.g. [Int] for a list of integers)
X → Y	Arrow type representing a function from X to Y. All functions are curried (i.e. arrows are chained to represent functions of multiple arguments). E.g. Int → [Int] → Bool takes an Int and a list of Ints and returns a Bool.

Supplementary Table 1 | Hindley-Milner typesystem used in the concept language. Lists the formal notation for various types and an English description of the notation's meaning.

**Usage**	**Type**	**Description**
(λ x body)	t1 → t2 → (t1 → t2)	lambda abstraction; binds x for use in body
0, 1, 2,...,99	Int	natural numbers
true, false	Bool	Boolean values
[]	[t1]	empty list
(+ x y)	Int → Int → Int	add x and y
(- x y)	Int → Int → Int	subtract y from x
(* x y)	Int → Int → Int	multiply x and y
(/ x y)	Int → Int → Int	quotient of x divided by y
(% x y)	Int → Int → Int	remainder of x by y
(< x y)	Int → Int → Bool	true if x is greater than y
(> x y)	Int → Int → Bool	true if x is less than y
(is_even x)	Int → Bool	true if x is even
(is_odd x)	Int → Bool	true if x is odd
(and x y)	Bool → Bool → Bool	Boolean conjunction of x and y
(or x y)	Bool → Bool → Bool	Boolean disjunction of x and y
(not x)	Bool → Bool	Boolean negation of x
(if p a b)	Bool → t1 → t1 → t1	a if p is true, else b
(== x y)	t1 → t1 → Bool	true if x and y are structurally identical
(singleton x)	t1 → [t1]	list with a single element, x
(repeat x n)	t1 → Int → [t1]	list repeating x n times
(range i j n)	Int → Int → Int → [Int]	list of numbers from i to j, inclusive, counting by n
(cons x xs)	t1 → [t1] → [t1]	prepend x to xs
(append xs x)	[t1] → t1 → [t1]	append x to xs
(insert x i xs)	t1 → Int → [t1] → [t1]	insert x at index i in xs
(concat xs ys)	[t1] → [t1] → [t1]	concatenate xs and ys
(splice ys i xs)	[t1] → Int → [t1] → [t1]	insert ys into xs, beginning at index i
(first xs)	[t1] → t1	first element of xs
(second xs)	[t1] → t1	second element of xs
(third xs)	[t1] → t1	third element of xs
(last xs)	[t1] → t1	last element of xs
(nth i xs)	Int → [t1] → t1	element i of xs
(replace i x xs)	Int → t1 → [t1] → [t1]	replace element at index i in xs with x
(swap i j xs)	Int → Int → [t1] → [t1]	swap elements at indices i and j in xs
(cut_idx i xs)	Int → [t1] → [t1]	remove element at index i from xs
(cut_val x xs)	t1 → [t1] → [t1]	remove first occurrence of x from xs
(cut_vals x xs)	t1 → [t1] → [t1]	remove all occurrences of x from xs
(drop n xs)	Int → [t1] → [t1]	remove first n elements from xs
(droplast n xs)	Int → [t1] → [t1]	remove last n elements from xs
(cut_slice i j xs)	Int → Int → [t1] → [t1]	remove elements at indices i to j, inclusive from xs
(take n xs)	Int → [t1] → [t1]	first n elements of xs
(takelast n xs)	Int → [t1] → [t1]	last n elements of xs
(slice i j xs)	Int → Int → [t1] → [t1]	sublist of xs from indices i to j, inclusive
(fold f acc xs)	(t2 → t1 → t2) → t2 → [t1] → t2	iteratively accumulate elements of xs into acc via f
(foldi f acc xs)	(Int → t2 → t1 → t2) → t2 → [t1] → t2	like fold, but p is also given the element's index
(filter p xs)	(t1 → Bool) → [t1] → [t1]	keep only elements of xs for which p is true
(filteri p xs)	(Int → t1 → Bool) → [t1] → [t1]	like filter, but p is also given the element's index
(count x xs)	(t1 → Bool) → [t1] → Int	count occurrences of x in xs
(find p xs)	(t1 → Bool) → [t1] → [Int]	returns indices of xs for which p is true
(map f xs)	(t1 → t2) → [t1] → [t2]	apply f to each element of xs
(mapi f x)	(Int → t1 → t2) → [t1] → [t2]	like map, but f has access to each element's index
(group f xs)	(t1 → t2) → [t1] → [[t1]]	group elements, x, of xs based on the key, (f x)
(is_in xs x)	[t1] → t1 → Bool	true if x is in xs
(length xs)	[t1] → Int	length of xs
(max xs)	[t1] → Int	largest element in xs
(min xs)	[t1] → Int	smallest element in xs
(product xs)	[Int] → Int	product of elements in xs
(sum xs)	[Int] → Int	sum of elements of xs
(unique xs)	[t1] → [t1]	unique elements of xs
(sort f xs)	(t1 → Int) → [t1] → [t1]	sort elements, x, of xs by the output of (f x)
(reverse xs)	[t1] → [t1]	xs in reverse order
(flatten xs)	[[t1]] → [t1]	concatenates the list of lists, xs, into a list
(zip xs ys)	[t1] → [t1] → [[t1]]	join xs and ys into a list of two-element lists

Supplementary Table 2 | Primitives used to construct concepts. Lists the primitive applied to arguments, its type per Supplementary Table 1, and a description of the primitive's behavior.

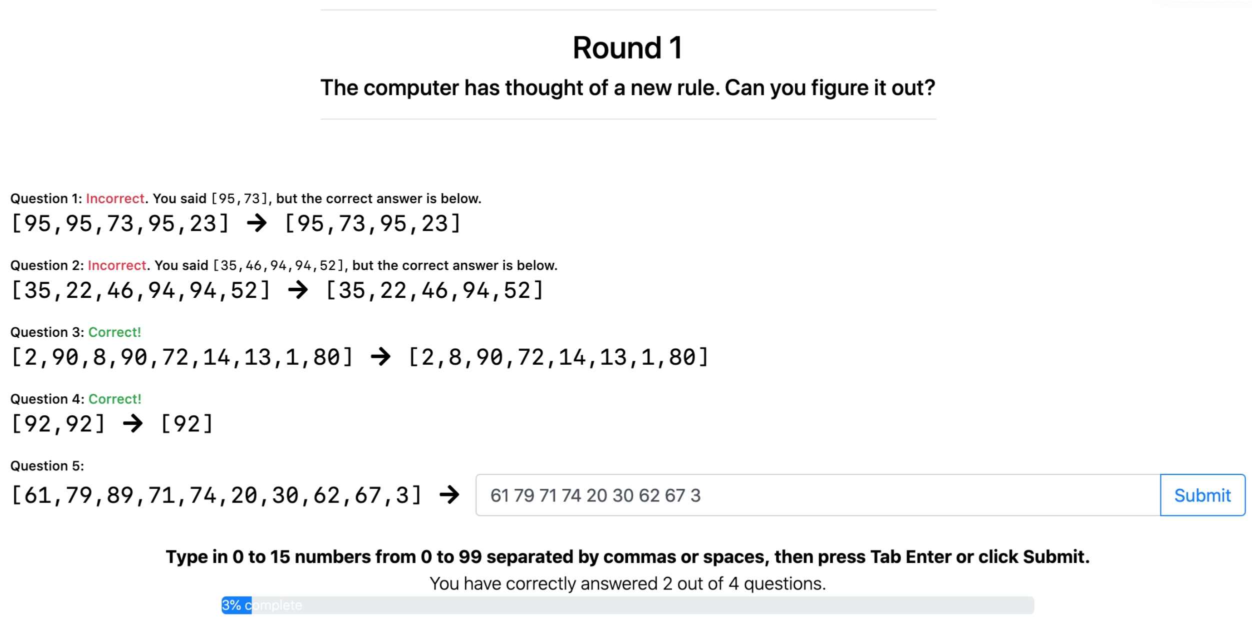

Supplementary Fig. 1 | Sample display from the behavioral paradigm. Previous trials remain onscreen, with a record of the input, output, participant response, and whether the response was correct. The concept displayed here is to *remove the first instance of the largest element in the input*.

Name	Logical Form	Description
**Identity**	$P(A, B) \leftarrow Q(A, B)$	P is Q
**Split**	$P(A, B) \leftarrow Q(A) \wedge R(B)$	Make independent assertions, Q and R, about P's arguments
**Pre-Condition**	$P(A, B) \leftarrow R(A) \wedge Q(A, B)$	P is Q with a pre-condition R
**Post-Condition**	$P(A, B) \leftarrow Q(A, B) \wedge R(B)$	P is Q with a post-condition R
**Chain**	$P(A, B) \leftarrow Q(A, C) \wedge R(C, B)$	Compose Q and R to explain P
**Memorize**	$P(A) \leftarrow \top$	Assert that P always holds for A
**Infer-A**	$P(B, C) \leftarrow Q(A, B, C)$	P is Q, assuming some latent argument A
**Infer-B**	$P(A, C) \leftarrow Q(A, B, C)$	P is Q, assuming some latent argument B

Supplementary Table 3 | Metarules used by the Metagol model. Lists an English title for each rule, the logical form which instantiations of the rule must take, and an English description of the rule's meaning.

Supplementary Note 1: Term Rewriting Systems

Executing MPL metaprograms produces program-like representations composed entirely of domain primitives. These are represented as term rewriting systems (TRS): formal systems that define both a space of tree structures called terms and a sequence of rules which can be used to derive new terms from existing ones.[1,2] MPL uses *first-order* terms; higher-order term rewriting systems have been studied, and place appropriately different constraints on what constitutes a term. Terms can be interpreted as programs, and the rules as the steps by which programs are evaluated. TRSs can thus be seen as self-contained programming languages.

The following situates the use of term rewriting in MPL. Formally, a *term rewriting system* is a pair, $T = (\Sigma, R)$. Σ is a *signature*, a set of symbols with fixed arity called *operators*. For example, a signature for a TRS describing Peano arithmetic might use one symbol with arity 2, $+$, one symbol with arity 1, S, and one symbol with arity 0, Z, written: $\Sigma_{PA} \equiv \{+/2, S/1, Z/0\}$. Operators can be combined with an infinite set of *variables*, $V \equiv \{v_1, v_2, v_3, \ldots\}$, to form a set of *terms*, $T(\Sigma, V)$, according to fixed rules of combination, namely: variables are terms; any operator of arity n applied to n terms is itself a term; and a term cannot contain itself as a subterm. For example, if $\mathcal{T} \equiv T(\Sigma_{PA}, V)$:

$$
\begin{aligned}
v_1 &\in \mathcal{T} && \text{(Variables are terms)} \\
Z &\in \mathcal{T} && \text{(Z has arity 0; it is a constant)} \\
+(S(Z)\ v_1) &\in \mathcal{T} && \text{(Non-constant operators take terms as arguments)} \\
v_1(Z) &\notin \mathcal{T} && \text{(Variables are terms, not operators)} \\
+(Z) &\notin \mathcal{T} && \text{($+$ has arity 2, not 1)} \\
K &\notin \mathcal{T} && \text{($K \notin \Sigma_{PA}$)}
\end{aligned}
$$

An *identity* is a pair $(\ell, r) \in T(\Sigma, V) \times T(\Sigma, V)$, commonly written as $\ell \approx r$. Let Var(t) be a function which takes in a term, t, and returns the set of variables, Var$(t) \subset V$, which appear in t. A *rewrite rule* is an identity where $\ell \notin V$ (i.e. ℓ is not a variable) and Var$(\ell) \supseteq$ Var(r) (i.e. no terms need be invented to complete the rewrite). R is a sequence of rewriting rules, $R \equiv \langle R_1,\ R_2, \ldots, R_n\rangle$. For example:

$$R_{PA} \equiv \langle +(x\ y) \approx +(y\ x),\ \ +(Z\ x) \approx x,\ \ +(S(x)\ y) \approx S(+(x\ y))\rangle$$

R defines a *rewrite relation*, $\rightarrow_R$, describing how terms may be rewritten. A *substitution*, σ, is a mapping from variables to terms. Applying a substitution to a term, t, written $t[\sigma]$, replaces variables in t with terms according to the map defined by σ. A term s can be rewritten to t by rule $\ell \approx r$ when there is some subterm s' of s and some substitution σ such that $\ell[\sigma] = s'$ and replacing s' with $t' = r[\sigma]$ in s equals t.

For example:

$$
\begin{aligned}
+(S(Z)\ +(x\ S(Z))) &\rightarrow_{R_{PA}} S(+(Z\ +(x\ S(Z)))) \\
&\rightarrow_{R_{PA}} S(+(x\ S(Z))) \\
&\rightarrow_{R_{PA}} S(+(S(Z)\ x)) \\
&\rightarrow_{R_{PA}} S(S(+(Z\ x))) \\
&\rightarrow_{R_{PA}} S(S(x))
\end{aligned}
$$

If viewing a TRS as a programming language, Σ defines the set of programs in a learner's mental language. It provides no information about how programs are evaluated to perform computations. This information is contained in R, and $\rightarrow_R$ defines an interpreter according to which these programs are executed. As the name suggests, this interpreter provides the semantics for a learner's mental programs. MPL imposes a decision procedure on the order in which rules can be applied, restricting the space of observed rewrites to a subset of $\rightarrow_R$. This decision procedure is called a *rewriting strategy*; the choice of rewriting strategy can dramatically affect the dynamics of rewriting. We specifically use *normal-order* evaluation (also called *leftmost-outermost*), which will terminate in finite time if a terminating sequence of rewrite steps exists—i.e. rewriting will eventually produce a term to which no further rewrite rules can be applied if such a term exists. Such a term is said to be in *normal form*. Even with normal-order evaluation, however, it is possible to construct terms that have no normal form—i.e. evaluation will never terminate. When more than one rule can be applied to the same subterm, we choose the one which appears first in R.

MPL includes in every TRS a fixed set of background rules implementing the domain primitives (Supplementary Table 4). These rules are automatically available and cannot be changed or removed. For computational efficiency, we rely on the host language to perform arithmetic operations like addition, subtraction, and numerical comparison. This approach effectively adds rules for thousands of specific arithmetic facts to every candidate TRS (e.g. `(+ 23 51)` $\approx$ `74`), but significantly reduces runtime and memory demands. It is also much faster than including general-purpose multi-step algorithms in every program.

Empty(Nil)	$\approx$	True
Empty(Cons(x y))	$\approx$	False
Equal(x x)	$\approx$	True
Equal(x y)	$\approx$	False
If(True x y)	$\approx$	x
If(False x y)	$\approx$	y
Head(Nil)	$\approx$	NAN
Head(Cons(x y))	$\approx$	x
Tail(Nil)	$\approx$	Nil
Tail(Cons(x y))	$\approx$	y

Supplementary Table 4 | Every MPL TRS includes this small, fixed set of background rules. Capitalized symbols denote operators and lower-case symbols denote variables. Addition (+), subtraction (-), and comparison (>) are evaluated directly in the host language.

Supplementary Note 2: Metaprimitives

This work introduces metaprimitives, primitive operators implementing structured program transformations. In particular, MPL includes two families of metaprimitives: observation primitives allow MPL to memorize data and inference primitives apply other kinds of program transformations. MPL combines these with more traditional domain primitives to construct *metaprograms*. Metaprimitives are higher-order intensional functions: they take a program (i.e. term rewriting system) as input, analyze its structure rather than its input/output behavior, and return a new program as output based on this analysis. Because term rewriting is not an intensional formalism, we implement metaprimitives directly in the host language rather than defining them using TRS rules. We briefly describe MPL's metaprimitives below and show example applications for each in Supplementary Table 5. Many metaprimitives are parameterized and represent a range of possible behaviors. For example, `DeleteRule` removes a rule from a TRS, but if the TRS contains more than one rule, the exact rule to be removed must be specified. Moreover, two TRSs may have different numbers of rules such that it may be possible to delete rule 9 from one and impossible to delete it from the other. We resolve these issues with the parameter ψ; it represents a randomly chosen real number in $[0, 1]$. When evaluated, a primitive computes the number of possible options and assigns each an equal portion of the range $[0, 1]$; whichever option is assigned to the range containing ψ is selected.

In what follows, each datum is an input/output pair, (i, o), where i represents some input term and o represents the output term produced by evaluating i. The data is a sequence of m input/output pairs, $D = \langle (i_1, o_1), (i_2, o_2), \ldots, (i_m, o_m) \rangle$. Some metaprimitives take as input a random number $\psi \in [0, 1]$. All metaprimitives take as input a TRS, $t = (\Sigma, R)$, and produce as output a TRS, t'. R is assumed to have length n. Most metaprimitives primarily affect the sequence of rules, R; for any two sequences, F and G, $F \mathbin{+\!\!+} G$ denotes the concatenation of F and G, and $F \backslash G$ filtering G from F, i.e. removing from F all elements syntactically identical to elements in G. Finally, if x and y are rules, then swap(F, x, y) denotes replacing all occurrences of x with y in F.

`MemorizeDatum(`t, ψ`)` is an observation metaprimitive that adds a single datum directly to t. MPL is designed to combine data-driven and hypothesis-driven approaches to learning. This metaprimitive helps MPL to be sensitive to the latent structure in data. Intuitively, it selects a random datum and inserts it as the first rule of the TRS, unless a syntactically identical rule already exists in t. Formally, `MemorizeDatum(`t, ψ`)` returns $t' = (\Sigma, R')$, where $R' = \langle i_j \approx o_j \rangle \backslash R \mathbin{+\!\!+} R$ and $j = \lceil \psi m \rceil$.

`MemorizeAll(`t`)` is an observation metaprimitive similar to `MemorizeDatum` except that it memorizes all the observed data rather than a single datum. Intuitively, it prepends the data to the sequence of TRS rules, unless syntactically identical rules already exist in t. Formally, `MemorizeAll(`t`)` returns $t' = (\Sigma, R')$, where $R' = D \backslash R \mathbin{+\!\!+} R$.

`DeleteRule(`t, ψ`)` is an inference metaprimitive that deletes a single rule from t. Intuitively, it uniformly samples a rule in the TRS and deletes it. Formally, `DeleteRule(`t, ψ`)` returns $t' = (\Sigma, R')$, where $R' = R \backslash \langle R_j \rangle$ and $j = \lceil \psi n \rceil$.

`Variablize(`t, ψ`)` is an inference metaprimitive that encodes the hypothesis that the repetition of some structure in a rule is more important than the form of the structure itself. Intuitively, it selects a single subterm in a single rule in t and replaces all its occurrences within the rule with a fresh variable. ψ jointly specifies the combination of rule and subterm on which the primitive operates. Formally, `Variablize(`t, ψ`)` returns $t' = (\Sigma, R')$, where $R' = \text{swap}(R,\ \ell_j \approx r_j, \ell_j[\sigma] \approx$

$r_j[\sigma]$), and $\sigma = [s_j \mapsto x]$ is a substitution from s_j to a fresh variable, x. $j = \lceil \psi k \rceil$ indexes into $V = \langle v_i \rangle_{i=1}^{k}$, a sequence containing all pairs $v_i = (s_i, \ell_i \approx r_i)$ such that $\ell_i \approx r_i$ is an element of R and s_i is a subterm of ℓ_i.

`AntiUnify(`t`)` is an inference metaprimitive that recursively attempts to anti-unify[1] each pair of rules in t. Anti-unification is a powerful tool for structural alignment and has appeared previously in program learning systems.[6,7] Intuitively, this metaprimitive looks for collections of rules which can be simplified by replacing the entire collection with a single generalized rule, computed using Plotkin's algorithm for first-order least-general generalization.[8,9] This process can be thought of as concurrently walking the tree structures of two rules. If the current position has similar structure in both rules (i.e. uses the same symbol), that structure is retained. If not, the differing structures are replaced with a variable. The algorithm tracks which terms are replaced by which variables so that the same variable is used whenever a given structure is encountered. Formally, `AntiUnify` splits R into a sequence of unexamined rules, U, and a sequence of examined rules, E. Initially, E contains just the first rule in R and U contains everything else, i.e. if $R = \langle r_i \rangle_{i=1}^{n}$, then initially $U = \langle r_i \rangle_{i=2}^{n}$ and $E = \langle r_1 \rangle$. `AntiUnify` then operates iteratively. On each iteration, the first unexamined rule in U, u_1, is paired with the first examined rule in E, e_1, to compute a least-general generalization, LGG(e_1, u_1). If this process produces a non-trivial least-general generalization—i.e. the result is a valid TRS rule—the new rule is retained in the examined list in place of the original rules. If on iteration k, $U_k = \langle u_1 \rangle +\!\!+ U'$ and $E_k = \langle e_i \rangle_{i=1}^{g}$, then $U_{k+1} = U'$ and $E_{k+1} = \langle \text{LGG}(u_1, e_1) \rangle +\!\!+ E_k$. If anti-unification fails, the unexamined rule, u_1, is compared to the next examined rule, e_2. If no generalization can be computed between u_1 and any element of E, u_1 is appended to E. When U is empty, `AntiUnify(`t`)` returns $t' = (\Sigma, E)$.

`Compose(`t, ψ`)` is an inference metaprimitive implementing the hypothesis that the effect of some function is actually best explained as a composition of two functions. Intuitively, it looks for a subterm occurring on the left-hand side of one or more rules which represents a function taking as input a linearly recursive structure (e.g. list, unary number, string) and returning that same type as output. It then replaces these rules with new ones which explain the original rule as the result of calling two new helper functions. The behavior of the helper functions is computed based on the rules being replaced.

Formally, `Compose(`t, ψ`)` returns $t' = (\Sigma', R')$, computed in the following way. The metaprimitive constructs a sequence, $C = \langle c_i \rangle_{i=1}^{k}$, of all possible compositions in R. For a given rule $\ell_i \approx r_i$ in R, the 4-tuple $c = (s,\ p_l,\ p_r,\ e)$ is a composition if s is a subterm of ℓ_i, p_l is the location at which s appears in ℓ_i (using a standard definition of location[1]), e is a type such that s is of type $e \to e$, and p_r is a location in r_i such that the subterm located there has type e. `Compose` then selects c_j, where $j = \lceil \psi k \rceil$. Then, Σ' introduces two new symbols of type $e \to e$ such that $\Sigma' = \Sigma \cup \{f : e \to e,\ g : e \to e\}$. Next, `Compose` creates three empty sequences, P, F, and G, and gives each rule $\ell_i \approx r_i$ a three-fold test: 1) ℓ_i pattern matches against $s\ x$; 2) $\ell_i|_{p_l}$, the subterm of ℓ_i at location p_l, is of type e; and 3) $r_i|_{p_r}$, the subterm of r_i at location p_r, is of type e. If $\ell_i \approx r_i$ passes the test, it is appended to P, $f\ \ell_i|_{p_l} \approx r_i|_{p_r}$ is appended to F and $g\ r_i|_{p_r} \approx r_i$ is appended G. Once all the rules have been processed, R' is constructed such that

[1]Unification is a fundamental concept in computer science with a long history.[3–5] It is the practice of substituting values for variables in a pair of expressions to find a most-general specialization of the two expressions. If variables in an expression are treated as gaps to be filled, unification finds a way to fill those gaps in a pair of terms such that they become the same term. The related concept of anti-unification looks for a way to replace values with variables to find a least-general generalization. It looks for ways to introduce gaps such that two terms become the same.

$R' = \langle s\ x \approx f\ (g\ x)\rangle \mathbin{+\!\!+} F \mathbin{+\!\!+} G \mathbin{+\!\!+} R\backslash P$. `Compose` implements a limited form of composition: more general approaches would have dramatically increased the implementation complexity of the metaprimitive.

`Recurse`(t, ψ) is an inference metaprimitive implementing the hypothesis that one or more rules in a TRS are best explained using recursion. While there are many kinds of recursion, this metaprimitive focuses on a limited form of linear structural recursion most similar to `concatMap`, a standard higher order function also known as `flatMap` or `mapCat` and available in many list processing libraries.[10–14] `concatMap` applies a function to each element of a list which takes the element as input and produces a list as output. It then concatenates these lists together to form a final output. `Recurse` similarly focuses on cases where what gets passed to the recursive call is any substructure of the input argument of the same type as the input argument. In the case of lists constructed through `cons` cells, this could be the tail of the list, or the tail of the tail, etc. In the case of lists, this ability to operate over multiple list elements before making a recursive call makes `Recurse` slightly more general than `concatMap`, which processes the input list exactly one element at a time. `Recurse` could also be applied to other linear structures (e.g. Peano/unary numbers, strings) and could also be used to reason about non-linear structures in a linear way (e.g. extracting a path from a binary tree).

Intuitively, `Recurse` identifies individual rules involving these kinds of functions and decomposes them into a series of rules involving either a general case partially processing the recursive structure in terms of a recursive call, or a set of base cases which terminate the recursion. `Recurse` typically makes a TRS longer but exposes repeated structure that can often be abstracted away by a mechanism like `AntiUnify` or `Variablize`.

Formally, `Recurse`(t, ψ) returns $t' = (\Sigma, R')$, where R' is computed in the following way. The metaprimitive constructs a sequence, $C = \langle c_i \rangle_{i=1}^{k}$, of all possible recursions in R. Recursions are 4-tuples in the same way as for `Compose`. `Recurse` then selects c_j, where $j = \lceil \psi k \rceil$. Next, `Recurse` creates three empty sequences, P, F, and G, and gives each rule $\ell_i \approx r_i$ a three-fold test: 1) ℓ_i pattern matches against $s\ x$; 2) $\ell_i|_{p_l}$, the subterm of ℓ_i at location p_l, is of type e; and 3) $r_i|_{p_r}$, the subterm of r_i at location p_r, is of type e. If $\ell_i \approx r_i$ passes the test, it is appended to P and iteratively processed to produce a series of rules. First, $r_i|_{p_r}$ in $\ell_i \approx r_i$ is replaced with $s\ \ell_i|_{p_l}$ to produce a new rule. If this new rule passes the same three-fold test as the original, it is appended to F and transformed in the same way as the original rule. This process repeats until reaching a rule which fails the three-fold test. This rule is appended to G. Once all the rules in R have been processed, R' is constructed such that $R' = F \mathbin{+\!\!+} G \mathbin{+\!\!+} R\backslash P$. As such, `Recurse` implements a limited form of recursion. In particular, it cannot handle non-linear recursion (e.g. traversing a binary tree) or any form of linear recursion which deviates from the pattern of adding to the structure returned by the recursive call. For list functions, this restriction precludes learning, e.g., `length` because it folds an entire list into a single element rather than extending the list with each recursive call.

involves maintaining and modifying state (e.g. computing the length of a list specifically by using an accumulator variable as opposed to, say, a right fold). Alternative metaprimitives handling these forms of recursion are a focus for future work.

`Generalize`(t) is an inference metaprimitive focused on the hypothesis that one or more rules which are identical in all but a few subterms actually represent specific instances of a more general rule. Intuitively, it replaces each discrepancy on the left-hand side with a fresh variable, and it replaces each discrepancy on the right-hand side with a call to a new function symbol taking the variables from the left-hand side as arguments. It removes the original rules and replaces them with

a copy of the generalized rule, as well as specific examples of each newly introduced function. It thus adds one or more subproblems directly to the output TRS, which can be improved by future mechanisms.

Formally, `Generalize(t)` returns $t' = (\Sigma', R')$. To see how t' is constructed, consider that in term rewriting, a *context* is a term containing one or more *holes*, $\square$.[1] For example, if $S\,K\,(K\,S)$ is a term, then $S\,\square\,(K\,S)$ is a context. Contexts are so named because they form a context from which many complete terms can be created by *filling* the holes, i.e. replacing them with hole-free subterms. A context is a context *of* a term when the term can be created by appropriately filling the holes of the context. Here, we generalize the notion of a context to a *rule context*, a rule containing one or more holes, such as $S\,\square\,(K\,S) \approx \square\,K$. The first step of `Generalize(t)` is to compute $c = \text{LSRC}(R,\, x,\, y\, z)$, the largest rule context containing x holes on the left-hand-side and y holes on the right-hand-side and shared by at least z rules.[2] We can also write c as $\ell_c \approx r_c$.

Next, `Generalize` constructs $\Sigma' = O \cup \Sigma$ where O is computed as follows. Let $\langle h_{\ell i}\rangle_{i=1}^{x}$ be a sequence of the type associated with each hole in ℓ_c and $\langle h_{ri}\rangle_{i=1}^{y}$ be a sequence of the type of each hole in r_c. O is a set containing fresh operators which take as arguments terms of the types in $h_{\ell i}$ and return terms of each type in h_{ri}, $O = \{o_i : h_{\ell 1} \rightarrow \ldots \rightarrow h_{\ell x} \rightarrow h_{ri} \mid 1 \leq i \leq y\}$.

Finally, `Generalize` constructs $R' = \langle \ell_g \approx r_g \rangle +\!\!+ F +\!\!+ R \backslash P$ as follows. It creates a term ℓ_g by replacing each hole in ℓ_c with a fresh variable, also collected in the sequence V. It then creates a term r_g by replacing each hole in r_c with the term constructed by applying the corresponding newly created operator, o to the variables in V, i.e. $o\,v_1\,\ldots\,v_x$. It then creates two empty sequences, P and F. Each rule, $\ell_i \approx r_i$ in R, which fills c is then processed in the following way. First, it collects subterms in ℓ_i filling holes in ℓ_c into a sequence $(s_i)_{i=1}^{x}$. For each hole in r_c, with corresponding operator o, it then collects the value, v, filling that hole and creates a new rule $o\,s_1\,\ldots\,s_x \approx v$. It appends this new rule to F and appends $\ell_i \approx r_i$ to P. This process continues until all rules in R are considered.

[2]For the sake of efficiency, the current implementation limits x to one, y to three, and fixes z to 2.

MemorizeDatum(t, ψ)

```
# where datum 1: C [1, 38, 19, 4] = [19]
# where datum 2: C [31, 41, 59, 62, 5] = [59]
# where datum 3: C [68, 47, 3, 6, 0, 9, 77] = [3]
C [x, y, 19, z] = [19]
```

```
C [31, 41, 59, 62, 5] = [59]
C [x, y, 19, z] = [19]
```

MemorizeAll(t)

```
# where datum 1: C [1, 38, 19, 4] = [19]
# where datum 2: C [31, 41, 59, 62, 5] = [59]
# where datum 3: C [68, 47, 3, 6, 0, 9, 77] = [3]
C [31, 41, 59, 62, 5] = [59]
C [x, y, 19, z] = [19]
```

```
C [1, 38, 19, 4] = [19]
C [68, 47, 3, 6, 0, 9, 77] = [3]
C [31, 41, 59, 62, 5] = [59]
C [x, y, 19, z] = [19]
```

DeleteRule(t, ψ)

```
C [1, 38, 19, 4] = [19]
C [68, 47, 3, 6, 0, 9, 77] = [3]
C [31, 41, 59, 62, 5] = [59]
C [x, y, 19, z] = [19]
```

```
C [1, 38, 19, 4] = [19]
C [68, 47, 3, 6, 0, 9, 77] = [3]
C [x, y, 19, z] = [19]
```

Variablize(t, ψ)

```
C [w, x, y, z] = [y]
C [91, 12, 63, 42, 35] = [63]
```

```
C [w, x, y, z] = [y]
C [91, 12, x, 42, 35] = [x]
```

AntiUnify(t)

```
C [25] = []
C [0] = []
C [9, 25] = [9 | (C [25])]
C [81, 9, 25] = [81 | (C [9, 25])]
C [50, 0] = [50 | (C [0])]
C [28, 50, 0] = [28 | (C [50, 0])]
C [37, 28, 50, 0] = [37 | (C [28, 50, 0])]
```

```
C [x] = []
C [x | y] = [x | (C y)]
```

Compose(t, ψ)

```
C [15, 6, 38] = [0, 15, 6, 38, 99]
C [12, 71] = [0, 12, 71, 99]
C [74, 3, 8, 16] = [0, 74, 3, 8, 16, 99]
```

```
C x = g (f x)
f [15, 6, 38] = [15, 6, 38, 99]
f [12, 71] = [12, 71, 99]
f [74, 3, 8, 16] = [74, 3, 8, 16, 99]
g [15, 6, 38, 99] = [0, 15, 6, 38, 99]
g [12, 71, 99] = [0, 12, 71, 99]
g [74, 3, 8, 16, 99] = [0, 74, 3, 8, 16, 99]
```

Recurse(t, ψ)

```
C [81, 9, 25] = [81, 9]
C [37, 28, 50, 0] = [37, 28, 50]
```

```
C [25] = []
C [0] = []
C [9, 25] = [9 | (C [25])]
C [81, 9, 25] = [81 | (C [9, 25])]
C [50, 0] = [50 | (C [0])]
C [28, 50, 0] = [28 | (C [50, 0])]
C [37, 28, 50, 0] = [37 | (C [28, 50, 0])]
```

Generalize(t)

```
C [x 1] = [1 (* 2 x) (* x x)]
C [x 2] = [4 (* 4 x) (* x x)]
C [x 3] = [9 (* 6 x) (* x x)]
```

```
C [x y] = [(f y) (* (g y) x) (* x x)]
f 1 = 1
f 2 = 4
f 3 = 9
g 1 = 2
g 2 = 4
g 3 = 6
```

Supplementary Table 5 | MPL metaprimitives implement higher-order program transformations. Each metaprimitive is listed; the input program, $\mathtt{t}$, is listed immediately below; and one possible output is listed to the right, assuming the appropriate selection parameter, ψ.

Supplementary Note 3: Behavioral Results

This work uses human learning behavior to assess a collection of formal models rather than attempting to document any particular empirical phenomenon about learning. It is thus important that: 1) participants performed significantly above chance and below ceiling (the task is, overall, neither impossible nor trivial but moderately challenging); 2) performance on individual concepts ranges from floor to ceiling (the task captures a significant portion of the range of human learning abilities); and 3) Mean accuracy is a reasonable estimate of learning difficulty for each concept (learning difficulty can be easily estimated).

Participants performed significantly above chance and below ceiling. Assuming participants uniformly selected lists at random, the expected mean accuracy for our participant pool would be $\approx 9.9 \cdot 10^{-31}$. The observed mean accuracy across all 250 functions was 0.459, much greater than expected by chance ($t(249) = 30.43$, $p < 0.001$, effect size $= 0.459$, 95% CI $=$ $[0.429, 0.489]$) but also significantly lower than perfect performance ($t(249) = -35.84, p < 0.001$, effect size $= 0.459$, 95% CI $= [0.429, 0.489]$). In 10.2% of rounds, participants responded correctly to either all trials or all but the first, showing that while perfect performance is possible, it is relatively unlikely.

Individual functions vary significantly in difficulty. We assess two kinds of variance in our data: variation in overall mean accuracy between functions, and variation in mean accuracy between functions within a given participant. To assess variation in mean overall accuracy between functions (i.e. some functions were easier to respond to correctly than others), we computed the mean accuracy across all participants for each function in our dataset. Supplementary Fig. 2A illustrates these results. It shows the mean accuracy across all 11 trials for each function in our dataset. Performance varies significantly, with some functions at or approaching 90% mean accuracy (i.e. nearly all subjects respond correctly after the first trial) and others at 0% (i.e. no participant ever responded correctly to a single trial). The majority of functions, however, appear "moderately learnable" in that many subjects respond correctly to a significant number of trials. The resulting distribution appears spread evenly from floor nearly to ceiling with no notable plateaus or discrete levels of difficulty (min $=$ 0.000, 25% $=$ 0.256, 50% $=$ 0.491, 75% $=$ 0.666, max $=$ 0.903). A three-parameter logistic item-response theory (IRT) analysis confirms this variation, with estimated mean difficulties ranging from 0.003 to 0.916 (mean $=$ 0.446; Supplementary Fig. 3B). To replicate this result within subjects, we also computed the variation in mean accuracy between functions for each participant; the median variance was 0.099 (min $=$ 0.011, 25% $=$ 0.074, 75% $=$ 0.122, max $=$ 0.193). Nearly all participants showed significant variance in performance across concepts; a bootstrap analysis revealed only 3 participants in 392 (i.e. $< 1\%$) whose 95% confidence interval on performance variation across functions included 0.

While there are likely other forms of significant variation—e.g. in overall mean accuracy between participants and in mean accuracy between participants within a given function—these differences are likely due to factors that go beyond the scope of this study, such as motivation, environment quality, and distractability. Supplementary Fig. 2B, however, shows that modeling individual differences provides fertile ground for future work. It plots the distribution over the total number of correct responses for each subject. While participants behave relatively similarly to one another on the top 25 (mean entropy = 1.64 bits) and bottom 25 (mean entropy = 0.98 bits) functions, the distribution over correct responses is much more diffuse across the middle 200 functions (mean entropy = 2.39 bits). Two-sample t-tests show that the empirical entropies over correct responses

for these middle 200 functions are significantly higher than for either the top ($t(33.358) = 11.273$, $p < 0.001$, 95% CI $= [0.616, 0.887]$) or bottom 25 functions ($t(27.082) = 13.039$, $p < 0.001$, 95% CI $= [1.182, 1.623]$). The IRT analysis also estimates that z-scored measures of skill varied by as much as 4.76 between participants (Supplementary Fig. 3A).

Mean accuracy is a reasonable estimate of performance on each concept. Observed mean accuracy is highly correlated ($R^2 = 0.958$) with estimated difficulty in the IRT analysis (Supplementary Fig. 3C), suggesting that mean accuracy is an effective estimate of difficulty. High or low mean accuracy is not merely the result of a function being assigned to a pool of particularly high-performing or low-performing participants.

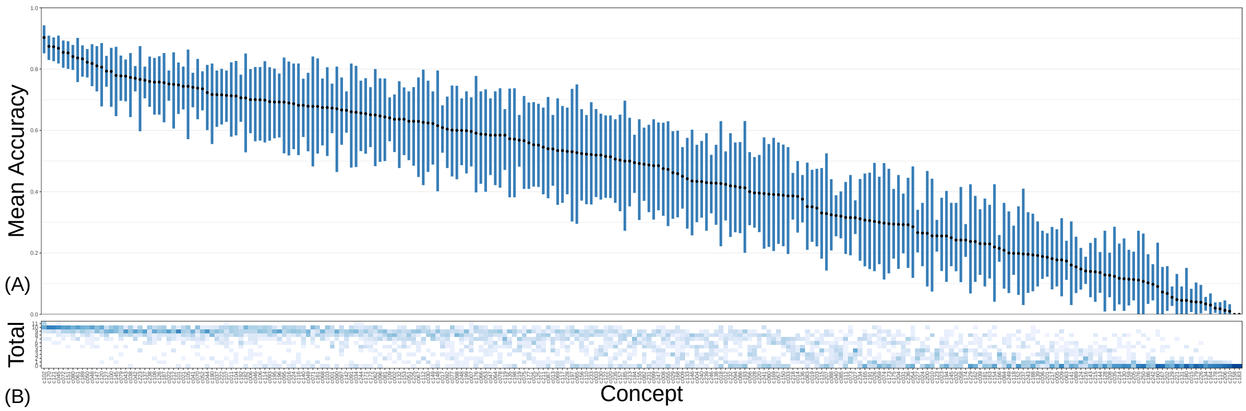

Supplementary Fig. 2 | Human performance varies from floor to ceiling without notable discretization. (A) Mean accuracy ($n = 392$ people) for each function (black dot) with bootstrapped 95% CI (blue bars). (B) The empirical distribution ($n = 392$ people) over correct responses for each concept. Darker squares indicate a higher proportion of participants gave a particular number of correct responses for a particular function.

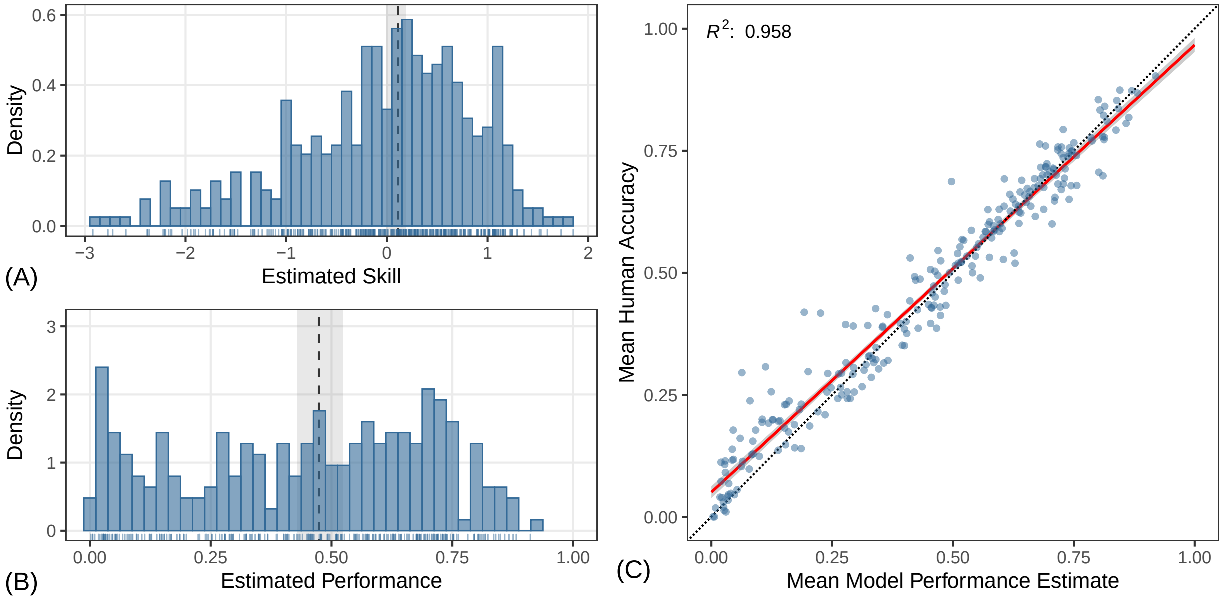

Supplementary Fig. 3 | Mean accuracy reasonably estimates function difficulty. Human item-response theory analysis. (A) Density of z-scored estimated participant skill ($n = 392$ people), with median (dotted line), 95% CI (gray bar), and individual estimates (rug plot). (B) Density of estimated function mean accuracy (250 total), with median (dotted line), 95% CI (gray bar), and individual estimates (rug plot). (C) Correlation between model estimated mean accuracy and human mean accuracy ($n = 392$ people) per function (250 total), with perfect correlation (dotted line) and linear fit to data (red line with 95% CI in gray).

Supplementary Note 4: Individual Learning Curve Analysis

Supplementary Fig. 4 compares individual learning curves for both MPL (5K) and humans, showing that MPL captures qualitative trends in human learning and behaves like a generally performant human. In most cases, MPL either outperforms (top row) or matches (middle row) the average human. This trend holds across a variety of mean accuracies. In some cases, however, MPL learns more slowly than the average human (bottom row, left) or fails to acquire functions which people learn quickly (bottom row, right). While there are cases where different MPL runs capture meaningfully different portions of the human distribution (e.g. c038, c096), MPL generally varies less across runs than humans vary across participants (e.g. c045, c009). While MPL may thus generally be a good model participant, it is less suitable for modeling average behavior on individual trials. The insets confirm this conclusion, showing that MPL's mean learning curves rarely match humans. Supplementary Fig. 5 shows individual learning curves for humans and MPL for all 100 functions in the model comparison.

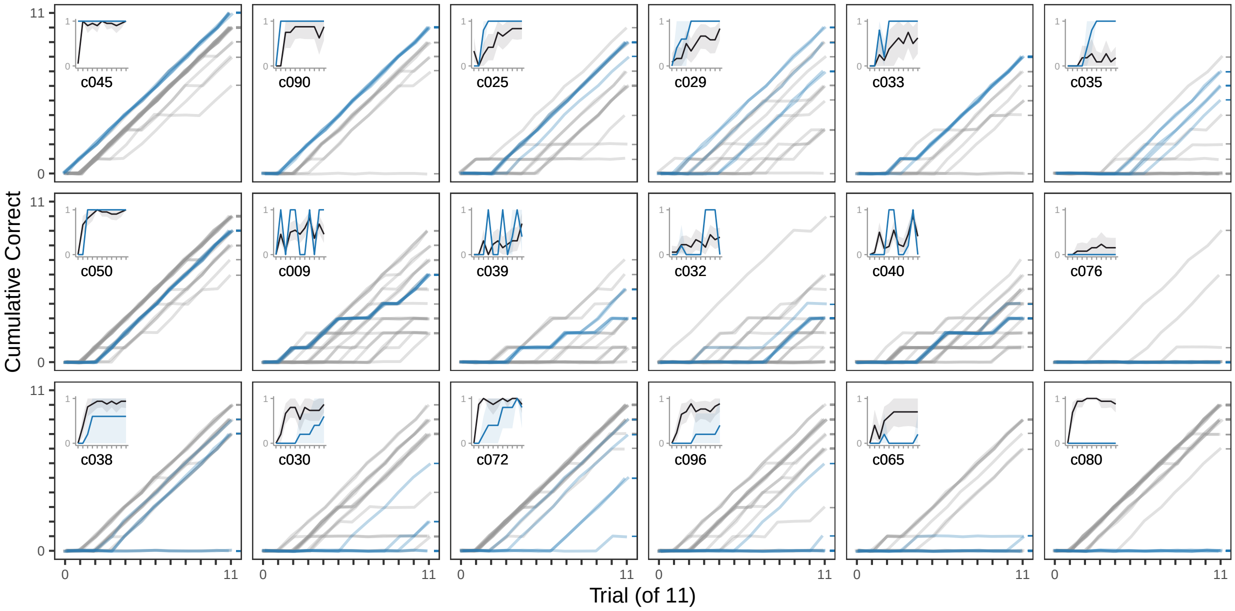

Supplementary Fig. 4 | MPL captures human trial-by-trial performance. Cumulative correct responses by a given trial (11 total) for individual humans ($n = 389$ people total; only those who completed a given function are shown; gray curves) and individual MPL (5K) runs ($n = 5$ total; blue curves) for eighteen representative functions. Panel position indicates MPL's learning rate relative to humans: above average (top row); average (middle row); below average (bottom row, left); or zero (bottom row, right). Each curve follows the progress of a single human participant or model run, plotting the number of correct responses by a given trial. Annotation lists function ID; inset shows mean accuracy by trial by source.

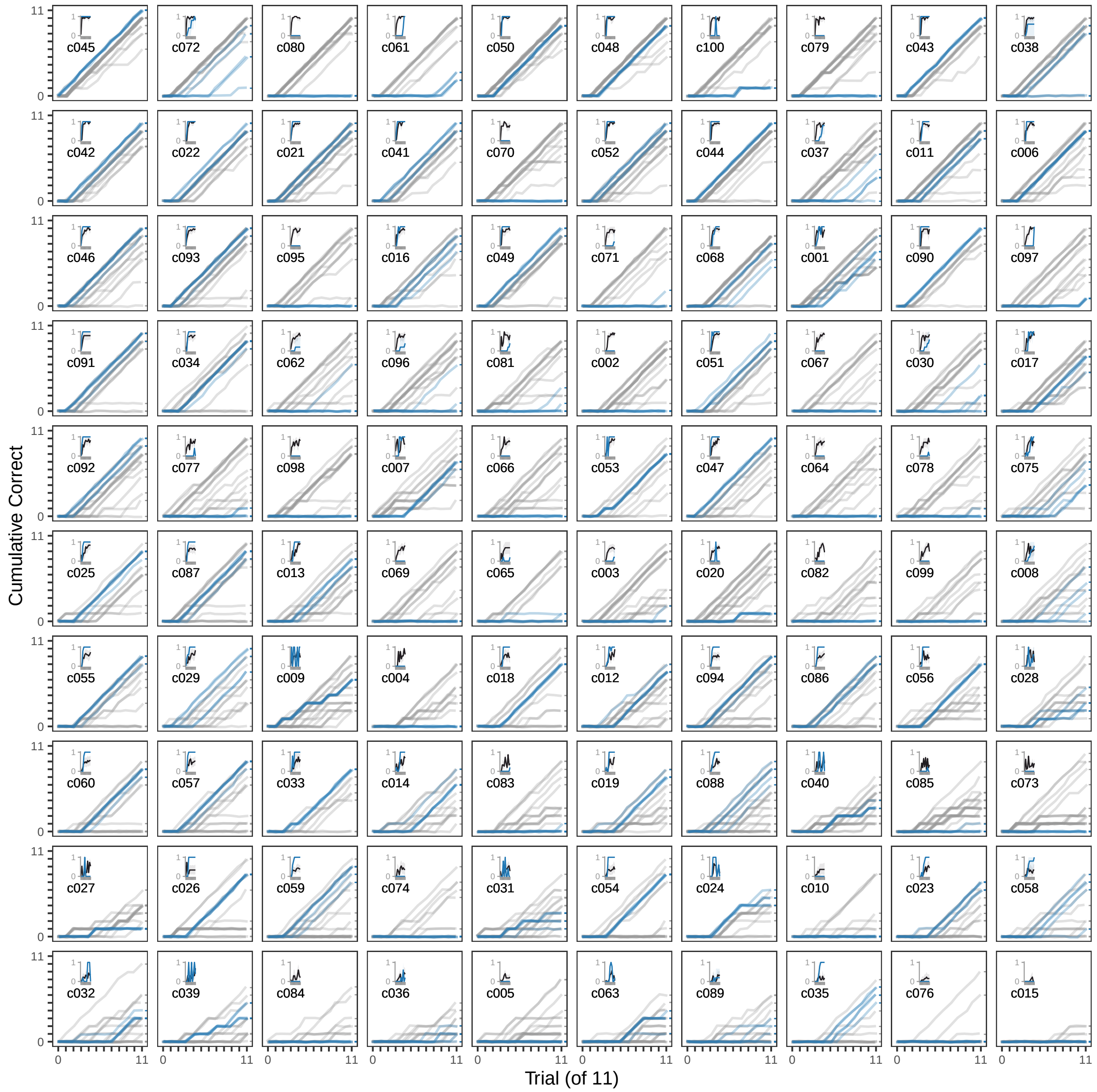

Supplementary Fig. 5 | Individual MPL learning curves approximate a human learner. The number of correct responses by a given trial (11 total) for both individual human participants $n(= 389$ people total; only those who completed a given function are shown; gray curves) and individual MPL runs ($n = 5$ total; blue curves) for the 100 functions in the model comparison (text annotation lists function ID) ordered by mean human accuracy (highest = top-left; lowest = bottom-right). Inset shows mean accuracy by trial by source.

Supplementary Note 5: Model Correlation and Error Analysis

Supplementary Fig. 6 plots the estimated density of mean accuracy across functions for each class of learner, as well as the correlation between these distributions. Model performances are generally correlated with one another and with human performance. In fact, Metagol is the only model which is not significantly correlated with human performance. It is also not significantly correlated with MPL (500K) performance, though it is significantly correlated with Fleet (500K) performance. Even so, Fleet (500K) and MPL (500K) are strongly correlated, largely because of agreement on extremes—i.e. the easiest and most difficult functions. For intermediate performances, they show broad disagreement. This disagreement is another illustration of the way that introducing metaprimitives dramatically reshapes inductive bias. One last point worth noting is that Codex predicts human mean accuracy better than any other model, though this also seems to be largely driven by the way Codex performance clusters toward extreme values.

Supplementary Fig. 7 repeats this analysis to compare versions of MPL and Fleet differing in their search budgets. For large search budgets, MPL and Fleet are highly correlated, which is to be expected. Both search the same space of programs, share a similar likelihood, and vary primarily in their inductive bias. For large search budgets, they should discover similar programs. Differences here perhaps show that the performance advantage of MPL's metaprograms require even greater amounts of search to completely eliminate. As search budgets decrease, Fleet and MPL become much less correlated with one another. The performance of MPL (500K), however, is strongly predictive of MPL (5K) performance; most differences in performance are due to improved performance for MPL (500K) for the functions with lowest performance for MPL (5K). Overall, these results show that MPL uses small amounts of resources more effectively than Fleet in this domain.

Mean accuracy and the rate at which learner's begin to display perfect performance are useful measures of learning and the focus of our main text comparison. They ignore, however, the detailed structure of the responses which humans and models made on our task. This analysis looks at that structure in more detail to better understand how well the responses of each model capture the kinds of responses humans were likely to make on similar problems.

First, for each response from each learner, we computed the edit distance between the learner's response and the correct response. We computed edit distance by converting each list into a standard string-based format (e.g. $[5,\ 87,\ 2,\ 91]\ \rightarrow$ "5 87 2 91") and then, because humans were required to type in responses character by character, computing the Levenshtein distance between these two strings. The Levenshtein distance between two strings, x and y, is the minimum number of insertions, deletions, and substitutions required to transform x into y. The edit distance is 0 only when the two strings are identical, which in this case only happens when the learner responds correctly. A non-zero edit distance indicates some sort of error, and the larger the edit distance, the more severe the error.

Supplementary Figure 8 plots the empirical distribution over edit distances for humans and each model learner. For humans, the prevalence of errors decreases as the severity of the error increases: 52.5% of trials have an edit distance of 0 (i.e. correct responses, matching our findings on overall accuracy); $\approx$$70\%$ have an edit distance ≤ 2; and only $\approx$$10\%$ have edit distances ≥ 10. MPL (500K), MPL (5K), and Fleet (500K) show similar results, though with marginally less in each column because these models occasionally fail to produce an answer. The remaining models perform notably differently. In sum, people tend to be correct or nearly correct on most trials, MPL and Fleet do the best job of capturing this distribution on error severity, and MPL does a better job

than Fleet on a reduced search budget.

A similar distribution over error severity does not imply that models are making errors on the same trials as humans. It is possible that they make similarly severe mistakes but do so on distinct trials. We therefore repeated our edit distance analysis for each model using human responses as the reference instead of the true correct response. Because multiple individuals provided responses for each trial, we computed an edit distance for each possible pair of model response and human response for each trial.

Supplementary Fig. 9 summarizes these results. Because two runs for a given model tend to vary less between than two people on a given trial, we plot results based on the median edit distance between each model response and the pool of human responses. No model responds correctly whenever humans respond correctly, though Codex perhaps comes closest to making correct predictions only when humans will also be correct. Moreover, models tend to be correct either when humans are correct or are close to the correct answer: cases where models are correct and people are incorrect occur most often for edit distances ≤ 3. By contrast, the cases where humans are correct and models are incorrect are not particularly concentrated at any edit distance. Similarly, when models and people are both wrong, they rarely give the same wrong response, though selecting based on the median edit distance makes such a result unlikely.

Supplementary Tables 6–10 provide a sampling of concrete responses to illustrate the trends summarized above. They show cases where the models both agree and disagree with humans, either correctly or incorrectly. Each table could contain tens of thousands of rows; the included rows are randomly sampled and intended to illustrate the findings above more concretely.

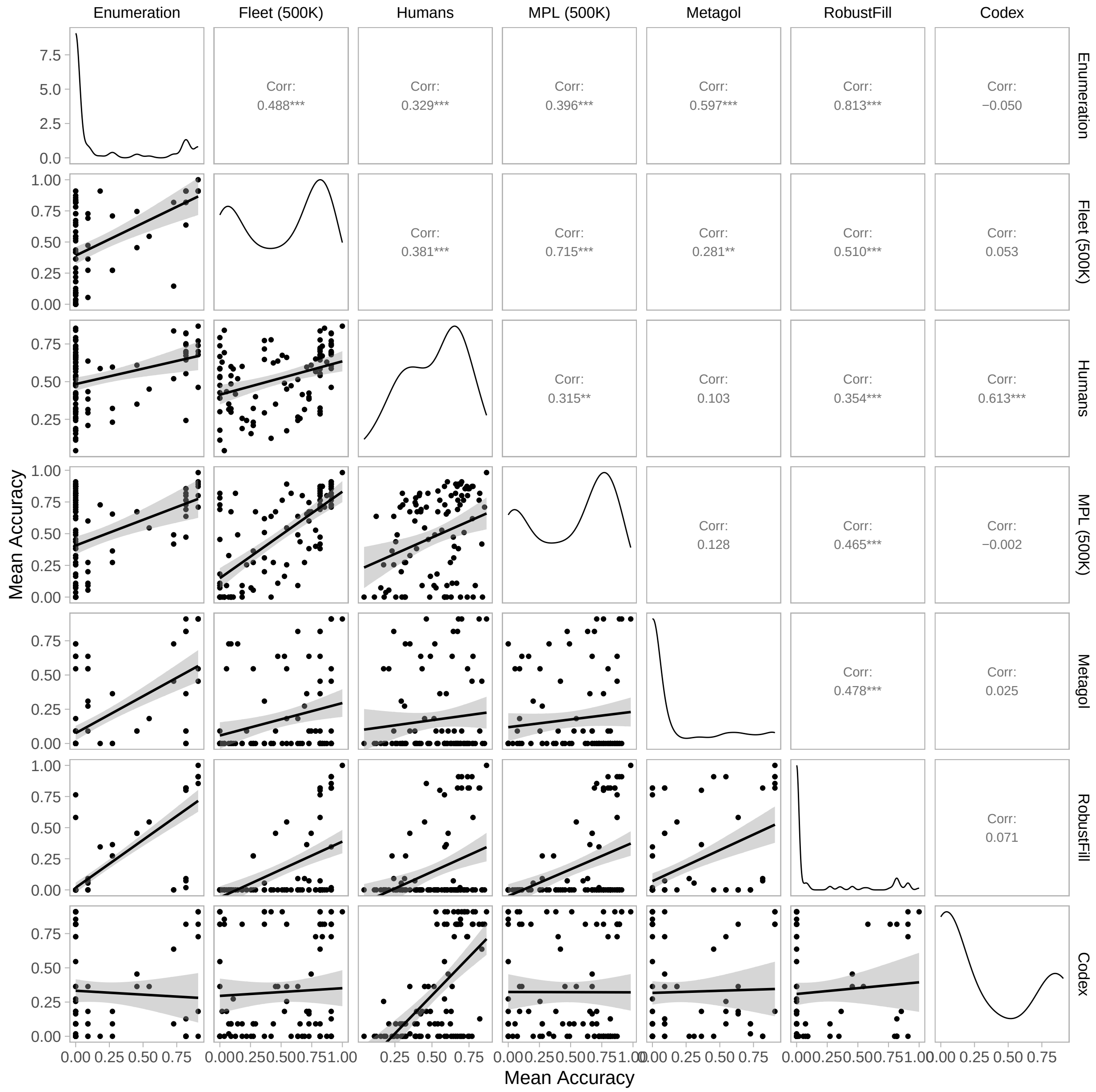

Supplementary Fig. 6 | Correlation Effects. Panels on main diagonal shows estimated density of mean accuracy across 100 functions for various classes of learners ($n = 389$ people). Panels below main diagonal shows Pearson correlation of mean accuracy between models with line of best fit. Panels above diagonal report Pearson correlation numerically.

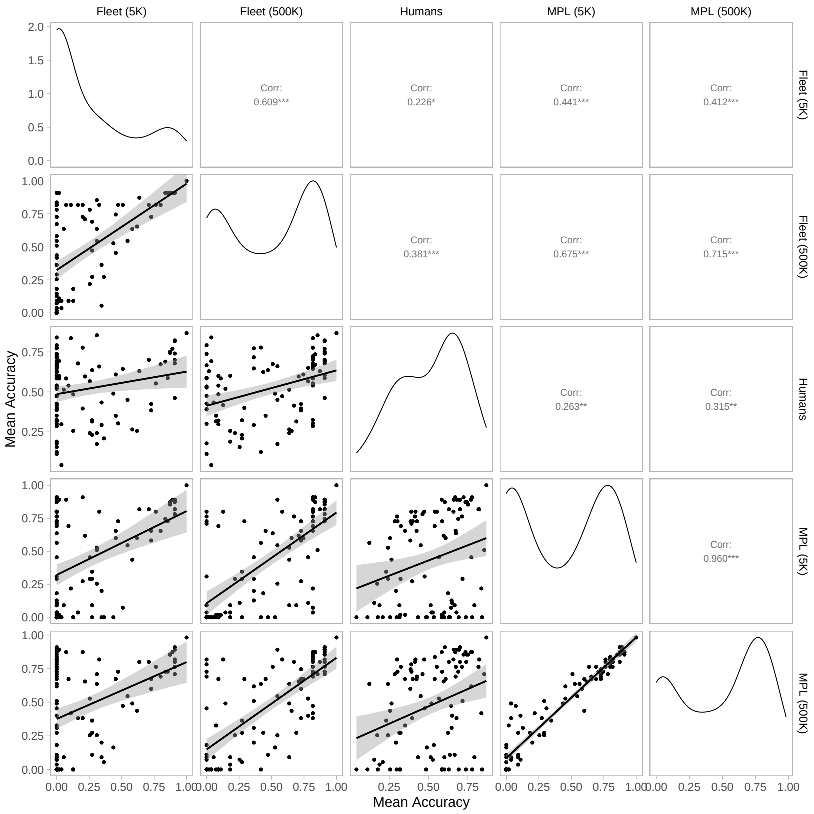

Supplementary Fig. 7 | Fleet and MPL become less correlated as search budgets decrease. Panels on main diagonal shows estimated density of mean accuracy across 100 functions for versions of MPL and Fleet differing in search budget. Panels below main diagonal shows Pearson correlation of mean accuracy between models with line of best fit. Panels above diagonal report Pearson correlation numerically.

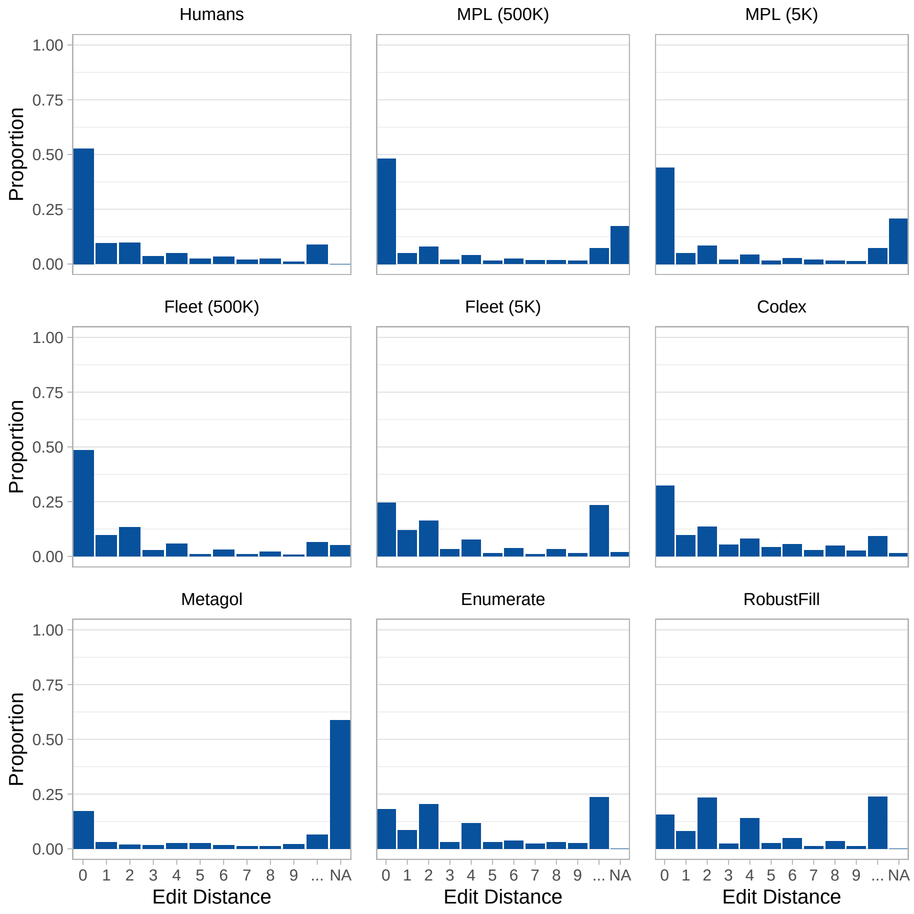

Supplementary Fig. 8 | Humans, MPL, and Fleet (500K) are typically nearly correct. Each bar represents the proportion of responses (y-axis) requiring exactly n edits (i.e. insertions, deletions, or substitutions; x-axis) to match the target response, displayed for each class of learner (panels; $n = 389$ people). $n \geq 10$ is displayed as $\ldots$; cases where a model gave no response are displayed as NA.

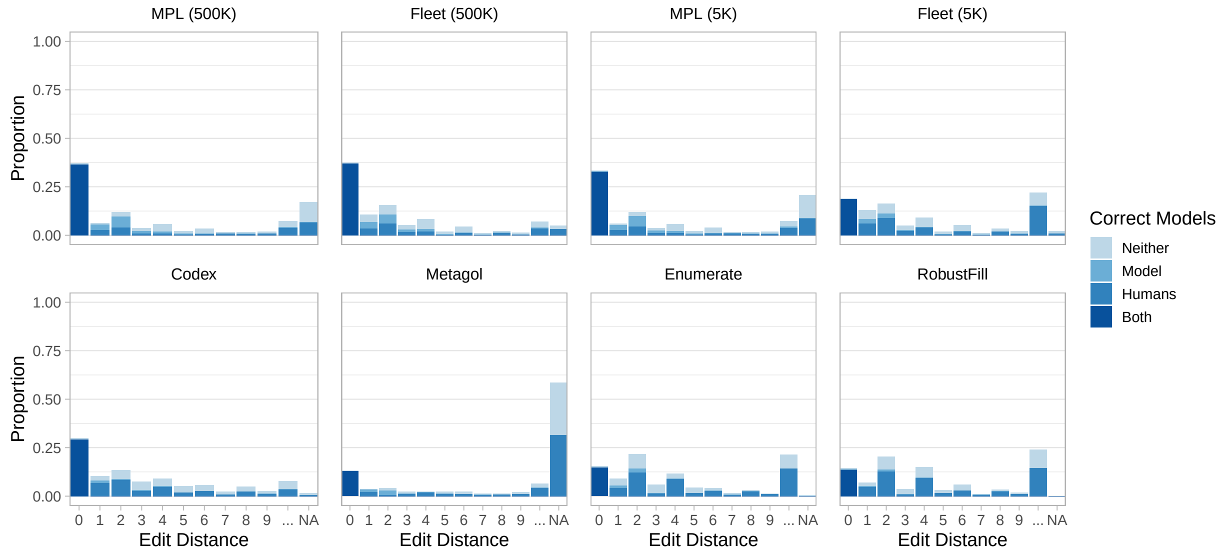

Supplementary Fig. 9 | Errors from MPL and Fleet (500K) are most similar to human errors. Following the previous figure, each bar represents the proportion of responses (y-axis) requiring exactly n edits (i.e. insertions, deletions, or substitutions) to match human responses ($n = 389$ people), displayed for each model (panels). Bars are colored based on whether humans or models gave incorrect responses for a given trial.

ID	Input	Output	Human Response	Model	Model Response	Δ_H	Δ_M	Δ_C
c037	$[9, 9]$	$[9, 9, 3]$	$[9, 9, 3]$	Codex	$[9, 9, 3]$	0.00	0.00	0.00
c061	$[8, 2, 7, 6, 1, 8, 5, 9]$	$[9]$	$[9]$	MPL (500K)	$[9]$	0.00	0.00	0.00
c002	$[7, 9, 0, 2, 1, 8, 6, 3, 5, 4]$	$[0]$	$[0]$	MPL (500K)	$[0]$	0.00	0.00	0.00
c065	$[6, 6, 3, 0, 0, 3]$	$[9, 6, 6, 3, 0, 0, 3, 7]$	$[9, 6, 6, 3, 0, 0, 3, 7]$	MPL (500K)	$[9, 6, 6, 3, 0, 0, 3, 7]$	0.00	0.00	0.00
c050	$[3, 2, 3, 0, 2, 0, 4, 3]$	$[3, 3, 2, 3, 0, 2, 0, 4, 3]$	$[3, 3, 2, 3, 0, 2, 0, 4, 3]$	Fleet (5K)	$[3, 3, 2, 3, 0, 2, 0, 4, 3]$	0.00	0.00	0.00
c050	$[5, 8, 5, 5, 5, 8, 8]$	$[5, 5, 8, 5, 5, 5, 8, 8]$	$[5, 5, 8, 5, 5, 5, 8, 8]$	Fleet (500K)	$[5, 5, 8, 5, 5, 5, 8, 8]$	0.00	0.00	0.00
c063	$[0, 5, 0, 5, 5, 7]$	$[5, 0, 5, 5, 7]$	$[5, 0, 5, 5, 7]$	Fleet (5K)	$[5, 0, 5, 5, 7]$	0.00	0.00	0.00
c052	$[1, 2, 4, 0, 3, 7]$	$[1, 1, 1, 1, 1, 1, 1, 1, 1, 1]$	$[1, 1, 1, 1, 1, 1, 1, 1, 1, 1]$	Codex	$[1, 1, 1, 1, 1, 1, 1, 1, 1, 1]$	0.00	0.00	0.00
c041	$[2, 2, 2]$	$[9]$	$[9]$	Fleet (500K)	$[9]$	0.00	0.00	0.00
c061	$[9, 2, 2, 5, 6, 2]$	$[2]$	$[2]$	MPL (5K)	$[2]$	0.00	0.00	0.00
c071	$[4, 5, 7]$	$[6, 7, 9]$	$[6, 7, 9]$	Codex	$[6, 7, 9]$	0.00	0.00	0.00
c041	$[4, 8, 7, 2, 6, 6, 5]$	$[9]$	$[9]$	RobustFill	$[9]$	0.00	0.00	0.00
c045	$[8, 8]$	$[8, 8]$	$[8, 8]$	MPL (5K)	$[8, 8]$	0.00	0.00	0.00
c050	$[3, 7]$	$[3, 3, 7]$	$[3, 3, 7]$	Codex	$[3, 3, 7]$	0.00	0.00	0.00
c049	$[1, 0, 9, 0, 2]$	$[0, 9, 0, 2]$	$[0, 9, 0, 2]$	Enumerate	$[0, 9, 0, 2]$	0.00	0.00	0.00
c048	$[5, 5, 5, 5, 5, 5]$	$[5]$	$[5]$	Codex	$[5]$	0.00	0.00	0.00
c070	$[7, 1, 1, 2, 1, 2]$	$[7, 1, 1, 2, 1, 2, 7, 1, 1, 2, 1, 2]$	$[7, 1, 1, 2, 1, 2, 7, 1, 1, 2, 1, 2]$	Codex	$[7, 1, 1, 2, 1, 2, 7, 1, 1, 2, 1, 2]$	0.00	0.00	0.00
c043	$[8, 7, 8, 8, 4, 7]$	$[8, 2, 7, 0, 3]$	$[8, 2, 7, 0, 3]$	MPL (500K)	$[8, 2, 7, 0, 3]$	0.00	0.00	0.00
c046	$[4, 7, 0, 5, 0, 3]$	$[7, 4, 7, 0, 5, 0, 3]$	$[7, 4, 7, 0, 5, 0, 3]$	MPL (5K)	$[7, 4, 7, 0, 5, 0, 3]$	0.00	0.00	0.00
c046	$[3, 8, 6, 7, 0, 3, 4]$	$[7, 3, 8, 6, 7, 0, 3, 4]$	$[7, 3, 8, 6, 7, 0, 3, 4]$	Metagol	$[7, 3, 8, 6, 7, 0, 3, 4]$	0.00	0.00	0.00

Supplementary Table 6 | Sampled cases where models and humans agree and are correct. Lists Function ID, the input and output for a specific trial, the response for one of the human participants, the response of one run of one of the models, the edit distance between the output and the human response (Δ_H), the output and the model response (Δ_M), and the human response and the model response (Δ_C).

ID	Input	Output	Human Response	Model	Model Response	Δ_H	Δ_M	Δ_C
c075	[0, 1, 1, 9, 2, 0, 1, 0, 9, 2]	[0, 1, 2, 1, 9]	[0, 2, 0, 0, 2]	Codex	[0, 2, 0, 0, 2]	4.00	4.00	0.00
c076	[6]	[6, 6, 1, 6, 6]	[6]	Codex	[6]	8.00	8.00	0.00
c069	[8]	[9, 3, 4, 0, 8, 7, 2, 9, 1]	[8]	RobustFill	[8]	16.00	16.00	0.00
c035	[3, 3, 3, 2, 2, 6]	[2, 3, 3, 3, 2, 6]	[3, 3, 3, 2, 2, 6]	RobustFill	[3, 3, 3, 2, 2, 6]	2.00	2.00	0.00
c077	[ ]	[0]	[ ]	RobustFill	[ ]	1.00	1.00	0.00
c038	[ ]	[9]	[ ]	Fleet (500K)	[ ]	1.00	1.00	0.00
c014	[6, 3, 0, 4]	[0, 4]	[ ]	Enumerate	[ ]	3.00	3.00	0.00
c077	[ ]	[0]	[ ]	Enumerate	[ ]	1.00	1.00	0.00
c036	[7, 0, 4, 1, 2, 9, 8, 6, 5]	[1, 0, 4, 7, 2, 9, 8, 6, 5]	[7, 4, 0, 1, 2, 9, 8, 6, 5]	MPL (500K)	[7, 4, 0, 1, 2, 9, 8, 6, 5]	4.00	4.00	0.00
c062	[1, 2, 5, 4, 5, 4, 7]	[1, 2, 5, 4, 5, 4]	[1, 2, 5, 4, 5, 4, 7]	RobustFill	[1, 2, 5, 4, 5, 4, 7]	2.00	2.00	0.00
c084	[85, 18, 85, 91, 91, 18, 91]	[91]	[ ]	Fleet (500K)	[ ]	2.00	2.00	0.00
c052	[9, 8, 7, 1]	[9, 9, 9, 9, 9, 9, 9, 9, 9, 9]	[9, 8, 7, 1]	Fleet (5K)	[9, 8, 7, 1]	15.00	15.00	0.00
c087	[36, 77, 25, 3, 1]	[36, 25, 77, 3, 1]	[36, 77, 25, 3, 1]	Fleet (5K)	[36, 77, 25, 3, 1]	4.00	4.00	0.00
c031	[7, 1, 1, 1, 8, 7, 7, 8, 1, 1]	[7, 1, 1, 1, 8, 7, 7, 8]	[1, 1, 8, 7, 7, 8, 1, 1]	MPL (5K)	[1, 1, 8, 7, 7, 8, 1, 1]	7.00	7.00	0.00
c067	[0, 0, 2]	[2, 0, 0]	[0, 0, 2]	RobustFill	[0, 0, 2]	2.00	2.00	0.00
c059	[5, 2, 4, 3, 7, 0, 8, 6]	[5, 2, 4, 6, 7, 0, 8, 3]	[5, 2, 4, 3, 7, 0, 8, 6]	Enumerate	[5, 2, 4, 3, 7, 0, 8, 6]	2.00	2.00	0.00
c081	[55, 74, 69]	[69]	[74]	Codex	[74]	2.00	2.00	0.00
c066	[5, 6, 5, 8]	[6, 5, 8, 5]	[5, 6, 5, 8]	Fleet (5K)	[5, 6, 5, 8]	4.00	4.00	0.00
c078	[7, 1, 5, 4, 0, 2, 8, 6]	[8]	[7]	Fleet (500K)	[7]	1.00	1.00	0.00
c031	[8, 8, 9, 2, 2, 3, 0, 5, 6]	[9, 2, 2, 3, 0, 5, 6]	[8, 8, 9, 2, 2, 3, 0, 5, 6]	RobustFill	[8, 8, 9, 2, 2, 3, 0, 5, 6]	4.00	4.00	0.00

Supplementary Table 7 | Sampled cases where models and humans agree and are incorrect. Lists Function ID, the input and output for a specific trial, the response for one of the human participants, the response of one run of one of the models, the edit distance between the output and the human response (Δ_H), the output and the model response (Δ_M), and the human response and the model response (Δ_C).

ID	Input	Output	Human Response	Model	Model Response	Δ_H	Δ_M	Δ_C
c052	[2, 5, 5]	[2, 2, 2, 2, 2, 2, 2, 2, 2, 2]	[5, 5, 5, 5, 5, 5]	Codex	[9, 9, 9]	14.00	17.00	9.00
c073	[4, 4, 4, 4]	[5, 6, 7, 8]	[4]	Codex	[4, 4, 4, 4]	7.00	4.00	6.00
c010	[4, 4, 4, 4, 4, 4, 4, 4, 4]	[4, 4, 4, 4]	[4, 4, 4]	MPL (5K)	[4, 4, 4, 4, 4, 4, 4, 4]	2.00	8.00	10.00
c064	[4, 5, 0, 0]	[5, 0]	[0]	Fleet (500K)	[4, 5, 0, 0]	2.00	4.00	6.00
c071	[0, 0, 7, 0]	[2, 2, 9, 2]	[1, 2, 3, 4]	MPL (500K)	[0, 0, 7, 0]	3.00	4.00	4.00
c084	[21, 24, 41, 38, 8, 6, 4, 96]	[4]	[21]	Fleet (5K)	[ ]	2.00	1.00	2.00
c065	[1, 3, 1, 5, 6, 4, 4, 3, 8]	[9, 1, 3, 1, 5, 6, 4, 4, 3, 8, 7]	[48]	Fleet (500K)	[9, 1, 3, 1, 5, 6, 4, 4, 3, 8]	19.00	2.00	17.00
c087	[21, 94, 56, 2, 0, 0, 21, 94]	[21, 56, 94, 2, 0, 0, 21, 94]	[21, 94, 2, 56, 0, 0, 21, 94]	Codex	[94, 56, 2, 0, 0, 94, 21, 21]	6.00	9.00	10.00
c003	[0, 5, 7, 4, 9, 1, 3, 6, 7, 8]	[3]	[5]	Enumerate	[0]	1.00	1.00	1.00
c063	[3, 3, 3, 3, 3, 3, 3, 3, 3, 3]	[3, 3, 3, 3, 3, 3]	[3]	Enumerate	[3, 3, 3, 3, 3, 3, 3, 3, 3, 3]	10.00	8.00	18.00
c005	[8, 2, 9, 3, 7, 6, 0, 5, 1, 4]	[1]	[0]	Enumerate	[8, 2, 9, 3, 7, 6, 0, 5, 1, 4]	1.00	18.00	18.00
c050	[0, 4, 8, 4, 0]	[0, 0, 4, 8, 4, 0]	[2, 4, 5, 6, 1]	MPL (5K)	[2, 0, 4, 8, 4, 0]	6.00	1.00	5.00
c040	[5, 7, 7, 3, 0, 2, 0, 6, 1]	[5, 7, 7, 3, 0, 2, 0, 6, 1, 3]	[5, 7, 7, 3, 0, 2, 0, 6, 1, 5]	Enumerate	[5, 7, 7, 3, 0, 2, 0, 6, 1]	1.00	2.00	2.00
c054	[7, 5, 2, 5, 7, 7, 2]	[2, 2, 2, 5, 7, 7, 2]	[7, 5, 5, 5, 2, 2, 2, 5, 5, 5, 7, 7, 2, 2, 2]	Codex	[7, 9, 2, 9, 7, 7, 2]	16.00	3.00	18.00
c063	[3, 3, 3, 3, 3, 3, 3, 3, 3, 3]	[3, 3, 3, 3, 3, 3]	[3]	Fleet (5K)	[3, 3, 3, 3, 3]	10.00	2.00	8.00
c075	[1, 7, 0, 8, 8, 3, 1]	[1, 0, 8, 1]	[1, 0, 8, 3]	MPL (5K)	[1, 0, 8, 1, 1]	1.00	2.00	3.00
c012	[7, 8, 2, 0, 4, 2]	[8, 2, 0]	[7, 8]	RobustFill	[3, 4, 7]	4.00	3.00	4.00
c057	[2, 7, 8, 4, 0, 6, 5, 1]	[2, 7, 8, 4, 0, 6, 4, 5, 1]	[2, 7, 8, 4, 0, 6, 5, 6, 1]	MPL (500K)	[2, 2, 2, 2, 2, 2, 4, 2]	2.00	8.00	9.00
c037	[9, 9]	[9, 9, 3]	[9, 9, 10]	Fleet (5K)	[9, 9]	2.00	2.00	3.00
c042	[9, 3, 8, 0]	[5, 2]	[0, 3, 6, 9]	MPL (5K)	[9, 3, 8, 0]	6.00	6.00	3.00

Supplementary Table 8 | Sampled cases where models and humans disagree and are both incorrect. Lists Function ID, the input and output for a specific trial, the response for one of the human participants, the response of one run of one of the models, the edit distance between the output and the human response (Δ_H), the output and the model response (Δ_M), and the human response and the model response (Δ_C).

ID	Input	Output	Human Response	Model	Model Response	Δ_H	Δ_M	Δ_C
c047	[9, 0]	[9, 6, 3, 8, 5, 9, 0]	[9, 9, 0, 6, 3, 8, 5, 6]	Fleet (500K)	[9, 6, 3, 8, 5, 9, 0]	7.00	0.00	7.00
c099	[95, 7]	[95]	[ ]	Fleet (5K)	[95]	2.00	0.00	2.00
c063	[0, 5, 0, 5, 5, 7]	[5, 0, 5, 5, 7]	[0, 5, 0, 5, 5, 7]	MPL (500K)	[5, 0, 5, 5, 7]	2.00	0.00	2.00
c048	[2, 1]	[2]	[3]	MPL (5K)	[2]	1.00	0.00	1.00
c050	[0, 4, 8, 4, 0]	[0, 0, 4, 8, 4, 0]	[2, 4, 5, 6, 1]	Fleet (500K)	[0, 0, 4, 8, 4, 0]	6.00	0.00	6.00
c010	[1, 0, 2, 1, 2]	[0]	[0, 2, 1, 2]	Fleet (500K)	[0]	6.00	0.00	6.00
c014	[2, 3, 0]	[0]	[ ]	Fleet (500K)	[0]	1.00	0.00	1.00
c028	[5, 2, 7, 9, 6, 4, 1, 0, 8, 3]	[5, 7, 9, 6, 4, 1, 0, 8, 3]	[2, 5, 3, 1, 5]	Fleet (5K)	[5, 7, 9, 6, 4, 1, 0, 8, 3]	12.00	0.00	12.00
c019	[6, 6]	[6, 6]	[6, 7]	Fleet (500K)	[6, 6]	1.00	0.00	1.00
c031	[3, 3, 5, 0, 0, 3]	[5, 0, 0, 3]	[3, 5, 0, 3]	Fleet (5K)	[5, 0, 0, 3]	2.00	0.00	2.00
c019	[8, 2, 6, 7, 9]	[8, 2, 6, 7, 9]	[8, 3, 6, 7, 9]	Enumerate	[8, 2, 6, 7, 9]	1.00	0.00	1.00
c058	[8, 2, 9, 1, 5, 3, 4, 0, 8, 6]	[0, 8, 6]	[8, 6]	MPL (500K)	[0, 8, 6]	2.00	0.00	2.00
c017	[9, 2, 1, 6, 4, 0]	[9, 8, 1, 6, 4, 0]	[9, 2, 8, 6, 4, 0]	Fleet (5K)	[9, 8, 1, 6, 4, 0]	2.00	0.00	2.00
c028	[7, 8, 0, 1, 0, 3]	[7, 8, 1, 0, 3]	[7, 8, 1, 3]	Fleet (500K)	[7, 8, 1, 0, 3]	2.00	0.00	2.00
c029	[7, 5, 1, 9, 4, 4, 4, 8]	[1, 9, 4, 4, 4, 8]	[1, 9, 4, 4, 8]	MPL (5K)	[1, 9, 4, 4, 4, 8]	2.00	0.00	2.00
c009	[0, 5, 2, 2, 2]	[0, 5, 2, 2, 2]	[0, 5, 2, 2]	Fleet (500K)	[0, 5, 2, 2, 2]	2.00	0.00	2.00
c095	[16, 51, 51, 16, 16, 0, 0, 85, 51, 9]	[51, 51, 16, 16, 0, 0, 85, 51]	[16, 51, 85]	Codex	[51, 51, 16, 16, 0, 0, 85, 51]	15.00	0.00	15.00
c041	[4, 8, 7, 2, 6, 6, 5]	[9]	[0]	Enumerate	[9]	1.00	0.00	1.00
c058	[3, 0, 1, 4, 8, 2, 7]	[ ]	[0, 1, 2, 3, 4]	Enumerate	[ ]	9.00	0.00	9.00
c031	[4, 4, 8, 3, 4, 9, 9, 9, 3, 6]	[8, 3, 4, 9, 9, 9, 3, 6]	[4, 4, 9]	MPL (500K)	[8, 3, 4, 9, 9, 9, 3, 6]	11.00	0.00	11.00

Supplementary Table 9 | Sampled cases where models are correct and humans are incorrect. Lists Function ID, the input and output for a specific trial, the response for one of the human participants, the response of one run of one of the models, the edit distance between the output and the human response (Δ_H), the output and the model response (Δ_M), and the human response and the model response (Δ_C).

ID	Input	Output	Human Response	Model	Model Response	Δ_H	Δ_M	Δ_C
c012	[4, 4]	[4]	[4]	Codex	[ ]	0.00	1.00	1.00
c034	[0, 8, 3, 1, 4, 9, 5, 9, 5]	[0, 3, 8, 1, 4, 9, 5, 9, 5]	[0, 3, 8, 1, 4, 9, 5, 9, 5]	Codex	[0, 8, 4, 3, 1, 9, 5, 9, 5]	0.00	4.00	4.00
c077	[2, 4]	[2]	[2]	MPL (5K)	[4]	0.00	1.00	1.00
c064	[3, 0, 3]	[0]	[0]	MPL (5K)	[0, 3]	0.00	2.00	2.00
c055	[9, 7, 7, 5, 5, 7]	[7, 5, 9, 7, 5, 7]	[7, 5, 9, 7, 5, 7]	Fleet (5K)	[7, 5, 5, 7]	0.00	4.00	4.00
c070	[5, 5]	[5, 5, 5, 5]	[5, 5, 5, 5]	MPL (500K)	[7, 1, 1, 2, 1, 2, 5, 5]	0.00	10.00	10.00
c024	[5, 2]	[5, 5, 2]	[5, 5, 2]	RobustFill	[5, 2]	0.00	2.00	2.00
c045	[1, 1, 2, 0]	[1, 1, 2, 0]	[1, 1, 2, 0]	Enumerate	[ ]	0.00	7.00	7.00
c022	[6, 4, 9, 5, 2, 7, 8, 2, 4]	[6, 5, 4, 9, 5, 2, 7, 8, 2, 4]	[6, 5, 4, 9, 5, 2, 7, 8, 2, 4]	Codex	[5, 6, 4, 9, 5, 2, 7, 8, 2, 4]	0.00	2.00	2.00
c059	[8, 3, 9, 2, 7, 4, 6, 3]	[8, 3, 9, 3, 7, 4, 6, 2]	[8, 3, 9, 3, 7, 4, 6, 2]	RobustFill	[8, 3, 9, 2, 7, 4, 6, 3]	0.00	2.00	2.00
c072	[6, 9, 2, 8, 0, 5]	[6, 6, 9, 9, 2, 2, 8, 8, 0, 0, 5, 5]	[6, 6, 9, 9, 2, 2, 8, 8, 0, 0, 5, 5]	Metagol	[6, 1, 3, 3, 3, 3, 7, 7]	0.00	15.00	15.00
c063	[0, 9, 7, 1, 1, 5, 4, 3, 6, 6]	[9, 7, 1, 1, 5, 4, 3, 6, 6]	[9, 7, 1, 1, 5, 4, 3, 6, 6]	RobustFill	[0, 9, 7, 1, 1, 5, 4, 3, 6, 6]	0.00	2.00	2.00
c018	[0, 6, 0, 6, 6, 6]	[0, 6, 0, 6, 6, 3]	[0, 6, 0, 6, 6, 3]	Fleet (5K)	[0, 6, 0, 6, 6, 6]	0.00	1.00	1.00
c076	[4, 8, 3]	[8, 3, 3, 4, 3]	[8, 3, 3, 4, 3]	Fleet (5K)	[8, 3]	0.00	6.00	6.00
c011	[9, 2, 6, 4, 6, 2, 0]	[2, 6, 4]	[2, 6, 4]	Enumerate	[9, 2, 6, 4, 6, 2, 0]	0.00	8.00	8.00
c087	[0, 9, 0, 68, 68, 9]	[0, 0, 9, 68, 68, 9]	[0, 0, 9, 68, 68, 9]	Metagol	[0, 25, 77, 3, 1]	0.00	8.00	8.00
c039	[3, 8, 6]	[3, 8, 6, 3]	[3, 8, 6, 3]	Fleet (5K)	[3, 8, 6]	0.00	2.00	2.00
c042	[6, 7, 3]	[5, 2]	[5, 2]	Codex	[0, 1]	0.00	2.00	2.00
c055	[8, 9, 4, 4]	[4, 4, 8, 9]	[4, 4, 8, 9]	Fleet (500K)	[4, 4]	0.00	4.00	4.00
c073	[1]	[2]	[2]	Fleet (500K)	[5, 6, 7, 8]	0.00	7.00	7.00

Supplementary Table 10 | Sampled cases where models are incorrect and humans are correct. Lists Function ID, the input and output for a specific trial, the response for one of the human participants, the response of one run of one of the models, the edit distance between the output and the human response (Δ_H), the output and the model response (Δ_M), and the human response and the model response (Δ_C).

Supplementary Note 6: Extended Search Budget Analysis

The main text reports that MPL preserves its human-like function acquisition rate even as resources drop by several orders of magnitude. Supplementary Fig. 10 shows a similar preservation of median error down to 50 steps/trial. Similar to the acquisition rate analysis, though, even MPL (10)'s error rate is notably better than Metagol, Enumerate, or RobustFill with abundant resources (main text Fig. 3B) or Fleet (5K) (main text Fig. 4C).

To better understand the efficiency gains introduced by metaprimitives, we created an auxiliary model which allowed MPL to search over both programs and metaprograms and allot variable proportions of the search budget to each. We first ran our auxiliary model for 5,000 search steps per trial while allocating equal amounts of search to programs and metaprograms. We denote this MPL_{aux} (5K). For all but 3 of the 100 functions, MPL_{aux} (5K) favored metaprogram-based hypotheses over program-based hypotheses or did not solve the problem. For each of the 3 exceptions, the model discovered a metaprogram whose posterior was within a factor of 20 of the program-based hypothesis. In 2 cases, these metaprograms reduced to the discovered program; it may not have done so for the third case due to the low search budget. Program search thus infrequently outperforms metaprogram search in our dataset. When it does, metaprogram search can either duplicate program-based solutions or find nearly equivalent alternatives.

Supplementary Fig. 11A shows how MPL_{aux} (5K) performance varies with the proportion of search time allocated to metaprograms versus programs. Consistent with MPL's robust performance in the face of resource constraints, performance remains stable even as the proportion of metaprogram search decreases. When metaprogram search is completely eliminated, performance drops significantly and is comparable to Fleet (5K)'s performance. In short, MPL's performance as an explanation of human learning depends on the use of metaprimitives.

We ran a separate simulation for 500,000 steps per trial—i.e. MPL_{aux} (500K)—and found similar results in terms of how frequently top hypotheses used metaprimitives. For all but 4 of the 100 functions, MPL_{aux} (500K) favored metaprogram-based hypotheses over program-based hypotheses or did not solve the problem. Three of these four also have metaprogram-based solutions whose posterior is within a factor of 20 of the program-based solution. Supplementary Fig. 11B shows how MPL_{aux} (500K) performance varies with the proportion of search time allocated to metaprograms versus programs. Because MPL is more robust to resource constraints than models lacking metaprimitives, we expect a wider performance gap at 5,000 search steps/trial than at 500,000. Because the lack or presence of metaprimitives alters a model's inductive bias, we also expect that, with sufficient search budget, a mixture of program-based and metaprogram-based solutions might outperform either individual model. Fig. 11B confirms these predictions. These results support the findings in the main text while also showing that given sufficient resources, program search and metaprogram search have complementary strengths that allow a model with access to both to learn more than it otherwise could.

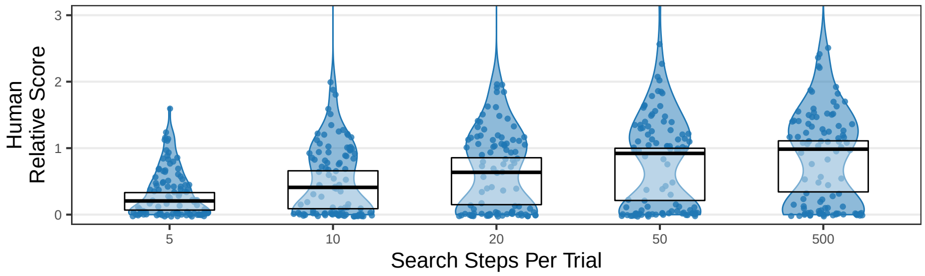

Supplementary Fig. 10 | MPL recovers median human mean accuracy with ≈50 search steps per trial. Ratio of MPL mean accuracy to human mean accuracy ($n = 389$ people) per function (100 total) per search budget, with parity between model and humans (dotted line), a kernel density estimate (colored regions) and median with 95% bootstrapped CI (crossbars).

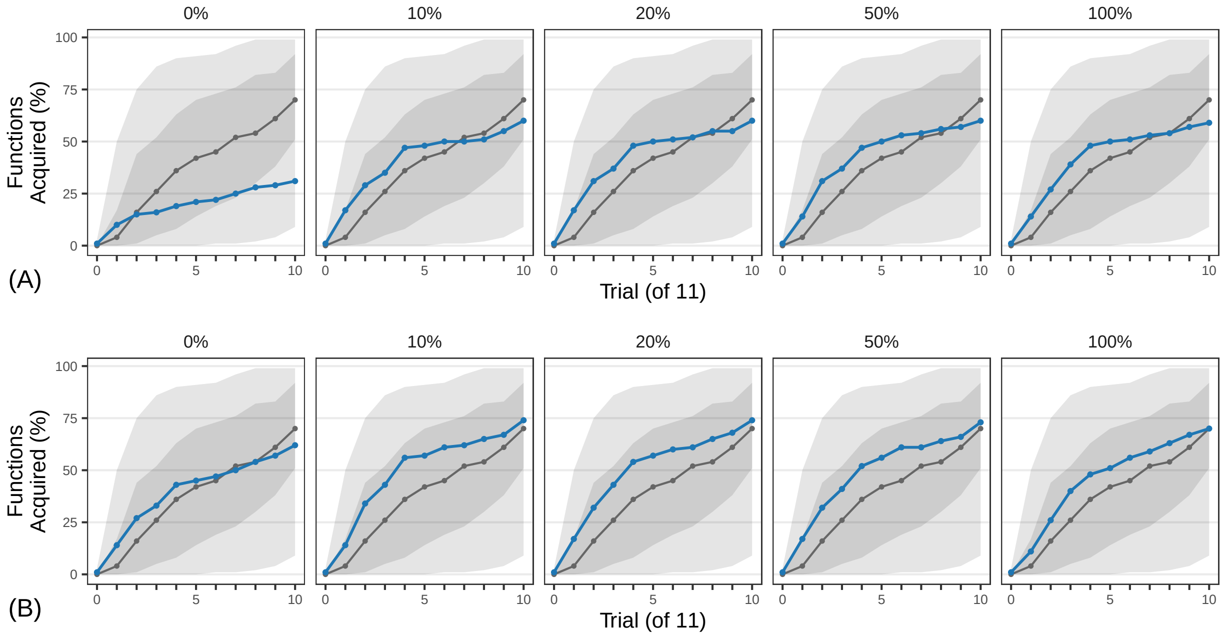

Supplementary Fig. 11 | Metaprogram search is efficient. Percentage of functions (100 total) acquired by an auxiliary MPL model dedicating various proportions its search budget to metaprogram vs. program search (subplots) by a given trial (11 total) with human median performance ($n = 389$ people; gray curve), 25%–75% human performance (dark gray band), and best–worst human performance (light gray band). We measure acquisition using the strict criterion of generating correct predictions on all future trials. Subfigures differ in search budget: (A) uses 5K steps per trial and (B) uses 500K steps per trial.

Supplementary Note 7: Extended MPL Simplicity Analysis

Supplementary Fig. 12 compares the prior probabilities of MPL metaprograms and the programs they generate for the highest-posterior hypotheses in each trial. This analysis appears in the main text (Fig. 5B), but here we replot it by trial. In trial 1, MPL has an empty set of observations and thus no data to memorize. In that case, the simplest solutions are constructed purely from domain primitives, so the metaprogram prior and program prior are equal. The points are mostly co-located because, without data to constrain the form of the program, MPL nearly always produces the simplest possible program. From trial 2 onward, however, MPL's behavior becomes more interesting and displays three overall trends. First, in each of trials 2–11, 81–83% of MPL's top hypotheses have metaprograms that are simpler to describe than the programs they produce. The simplicity benefits of metaprograms are consistently strong and present as soon as data becomes available. Second, additional data (and additional search) supports the discovery of increasingly complex metaprograms and programs. The mean $-\log_e$ metaprogram and $\log_e -\log_e$ program prior probability in trial 2 are 10.50 and 3.05 (i.e. $\ln 27.6$), respectively, and 25.90 and 3.78 (i.e. $\ln 120.9$), respectively, by trial 11. These distributions are significantly different for both metaprograms ($t(572.79) = -11.131$, $p < 0.001$, 95% CI = $[-18.07, -12.65]$) and programs ($t(781.59) = -9.865$, $p < 0.001$, 95% CI = $[-0.883, -0.590]$). Third, a single notable cluster forms in which the program is much simpler than the metaprogram. It contains cases where MPL discovers a complex way of creating the trivial program. Because a metaprogram which consists of solely the trivial program is excluded from search, this happens to be one of the simplest metaprograms that produces a trivial program. It is the simplest way for MPL to admit it doesn't have an answer.

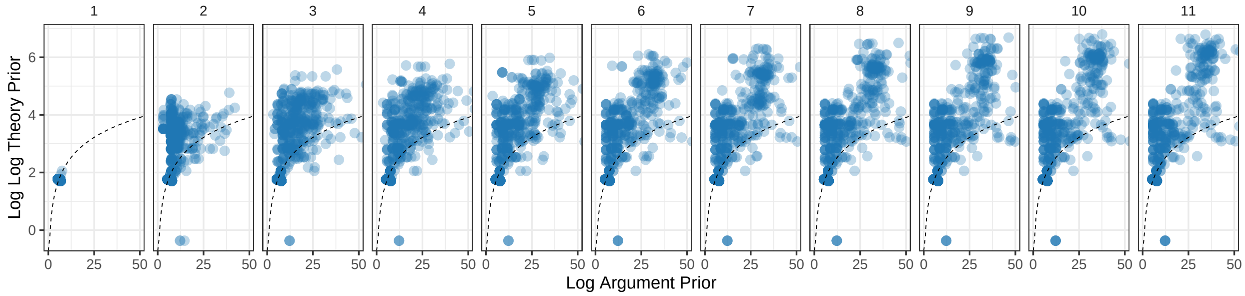

Supplementary Fig. 12 | MPL metaprograms are simpler than corresponding programs. MPL's $\log_e - \log_e$ program prior relative to MPL's $\log_e$ metaprogram prior per function (100 total; dots) per trial (11 total); dotted curve shows where the two are equal.

Supplementary Note 8: Targeted Human Behavioral Replication

To confirm our findings, we conducted a targeted replication, i.e. an additional behavioral experiment involving human participants. Our procedure is similar to that reported in the main text and complies with all relevant ethical regulations and was approved by the Institutional Review Board (IRB) at University of California Berkeley where the study was conducted. Participants provided informed consent and received a flat fee of \$16.00 for participating plus a \$0.05 bonus for each correct response. This study was not preregistered.

Our experiment focused on the 100 functions examined in the main text's model comparison. 183 people provided informed consent and participated in the experiment, hosted on Prolific using JATOS and JSPsych. 25 were excluded due to a technical error during data collection, and 1 more was excluded according to the exclusion criteria described in the main text. In addition to the 183 from whom we collected data, an additional 73 returned the task or timed out. Returning may happen when participants open the task and decline consent or revoke consent partway through the experiment. We do not have details on exactly when tasks were returned.

We analyzed data from the remaining 157 participants, where mean task time was 70.2min (95% CI [63.2, 77.1]), number of mean correct responses was 70.2 (95% CI [68.1, 72.4]), and mean number of appearances of the most common response was 7.5 (95% CI [7.1, 8.1]). Participant age for this group ranged from 19yrs to 86yrs (median: 34yrs), with 92 males and 64 females (1 participant did not report sex to Prolific). We did not actively assess language skills but requested that participants speak English fluently. Participants received a median compensation of \$19.60 for a median 54.8min of work. Participants found the task difficult but engaging with a mean self-reported difficulty rating of 4.1 and a mean self-reported engagement rating of 5.3, both on a 7-point Likert scale. Because each participant completed 10 rounds of trials, we collected data from about 16 participants for each list function.

Of the 157 participants in our sample, 65 (41%; mean accuracy = 0.62) indicate no prior programming experience, an additional 52 (33%; mean accuracy = 0.63) indicate social exposure to programming concepts and perhaps simple website construction. 25 (16%; mean accuracy = 0.67) report encountering programming through introductory coursework or by building several websites. Only 15 (10%, mean accuracy = 0.70) indicate significant academic or professional exposure to programming.

Supplementary Fig. 13A–B replicates Supplementary Fig. 2, showing again that humans provide a challenging target for model learners. While per-function mean accuracy is strongly correlated between the replication sample and the original sample (Supplementary Fig. 13C; $t(98) = 19.996$, $p < 0.001$, $r = 0.896$, 95% CI = $[0.849, 0.929]$), a paired t-test shows that replication participants outperform the original participants ($t(99) = 12.554$, $p < 0.001$, mean = 0.117, 95% CI = $[0.099, 0.136]$). Other analyses confirm these results. Mean accuracy averaged across functions was high (Mean = 0.638, 95% CI [0.600, 0.677]; SD = 0.208, 95% CI [0.178, 0.234]) relative to chance, and ranged from 0.080 to 0.903 for individual functions.

Similarly, the rate of function acquisition in the replication sample was highly correlated with the original sample ($t(9) = 11.087$, $p < 0.001$, $r = 0.965$, 95% CI = $[0.868, 0.991]$), but a paired t-test shows that the replication sample acquired functions significantly faster ($t(10) = 7.734$, $p < 0.001$, mean = 21.27, 95% CI = $[15.14, 27.40]$). Indeed, 80% of functions were acquired by $\geq 50\%$ of human learners within eight trials. 66% of functions were acquired by at least one person after a single trial, 84% after two trials, and fully 99% within eight trials. 11% were acquired by

all participants within eight trials.

These results are not surprising. Our original sample was collected using Amazon Mechanical Turk, and our replication sample was collected using Prolific. Recent behavioral comparisons have shown that participants on Prolific are more likely to pass attention checks, respond meaningfully, follow instructions, and work at an appropriate pace.[15] Our Prolific sample is more likely reflective of how people can perform on this task when engaged and motivated to participate.

Even so, the strong performance of the replication sample does not weaken any of the crucial points of our main argument. If anything, it reinforces the idea that people can learn algorithmically sophisticated concepts from relatively sparse data. Moreover, MPL still predicts human performance on this task better than the other tested models (Compare Supplementary Fig. 14A and with Fig. 3A and and Supplementary Fig. 14B with Fig. 3B.), particularly with plausible computational resources (Compare Supplementary Fig. 15A with Fig. 4B and Supplementary Fig. 15B with Fig. 4C.).

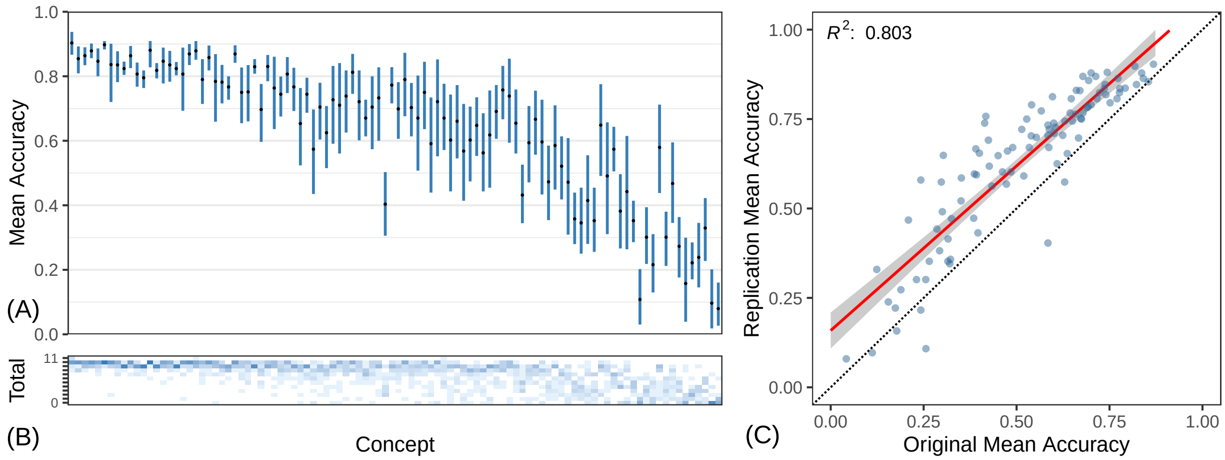

Supplementary Fig. 13 | Human performance in the replication sample varies from floor to ceiling without notable discretization. (A) Mean human accuracy ($n = 157$ people) in the replication sample for each function (100 total; black dots) with bootstrapped 95% CI (blue bars). (B) The empirical distribution over correct responses for each concept ($n = 157$ people). Darker squares indicate a higher proportion of participants gave a particular number of correct responses for a particular function. (C) Mean accuracy in the original sample ($n = 389$ people) compared with the replication sample ($n = 157$ people) for each function (100 total; blue dots) with a line of best fit (red curve; gray contour is 95%CI), along with a reference showing a perfect correlation (dotted line).

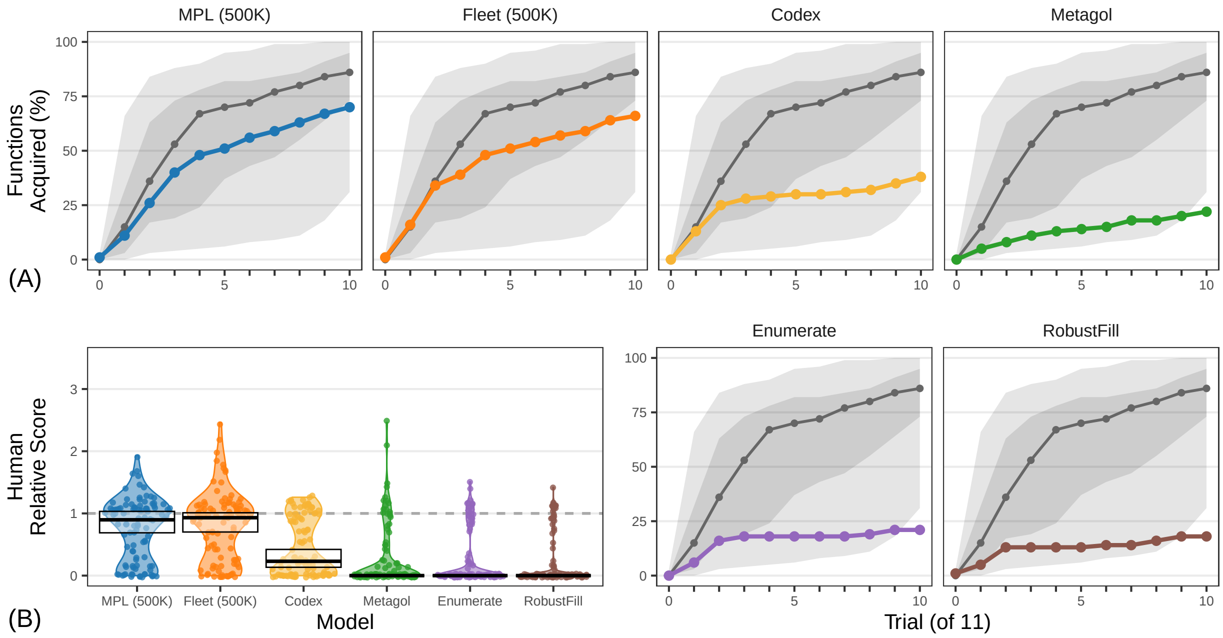

Supplementary Fig. 14 | MPL and Fleet remain closest to human performance in the replication sample. (A) Percentage of functions (100 total) acquired per model (subplots) by a given trial (11 total) with human median performance ($n = 157$ people; gray curve), 25%–75% human performance (dark gray band), and best–worst human performance (light gray band). We measure acquisition using the strict criterion of generating correct predictions on all future trials. (B) Ratio of model mean accuracy to human mean accuracy ($n = 157$ people) per concept (dots; 100 total) per model, with parity between models and humans (dotted line) and a kernel density estimate (colored regions). The crossbars show the median across functions with a 95% bootstrapped CI. Each model is associated with a unique color for easier comparison across figures.

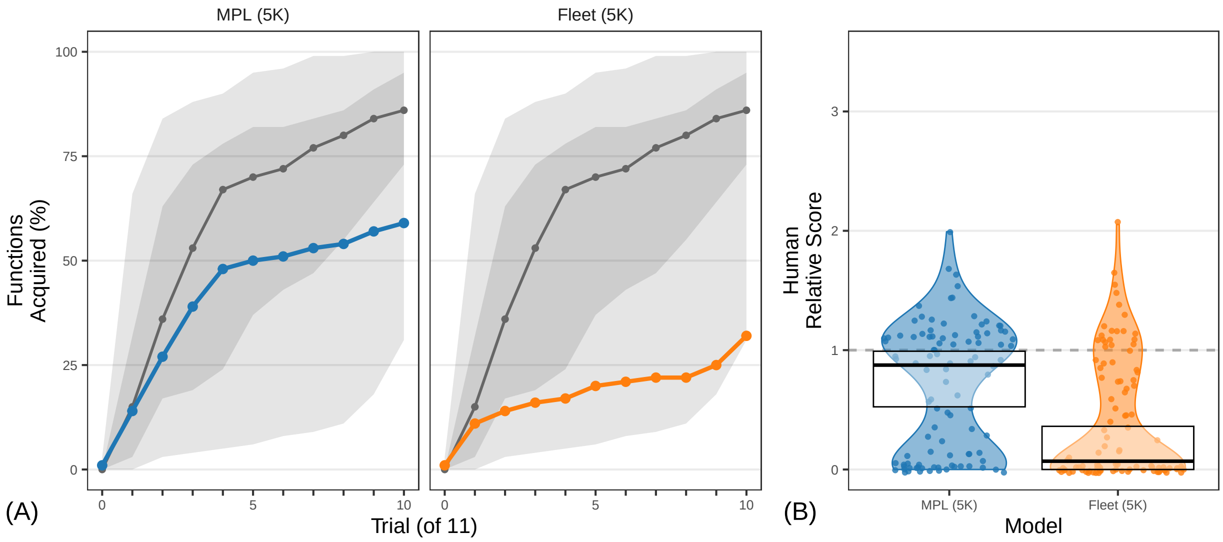

Supplementary Fig. 15 | With plausible resources, MPL predicts human performance in the replication sample better than Fleet. (A) follows Supplementary Figure 14A, but with only 5K search steps per trial. (B) similarly follows Supplementary Figure 14B, but with only 5K search steps per trial.

Supplementary Table 11 | The 250 list functions in our dataset vary significantly in difficulty and algorithmic content. Functions are sorted according to mean human performance (μ). Also listed are the function's ID, a natural language description, the description length ($\mathcal{L}$) and program in the DSL described in Extended Data Table 2, the description length ($\mathcal{L}$) and program in the DSL described in Table 1 (model comparison functions only), and the first five examples shown to participants. Functions may appear twice if tested both with the numbers 0–9 and 0–99. The model comparison involved c001–c100.

μ	ID/$\mathcal{L}$	Description, Program, & Examples
0.903	c102	*the input*
	2	`(λ (x) x)`
		[4, 72, 68, 63, 97] → [4, 72, 68, 63, 97]
		[79, 50, 92, 5, 8, 91, 27, 2, 43] → [79, 50, 92, 5, 8, 91, 27, 2, 43]
		[26, 86, 51] → [26, 86, 51]
		[0, 75, 58, 55] → [0, 75, 58, 55]
		[36, 57, 94, 1, 87, 38] → [36, 57, 94, 1, 87, 38]
0.874	c170	*remove all but element 1 and last element*
	12	`(λ (x) (cons (first x) (singleton (last x))))`
		[15, 4, 87, 8, 64, 14] → [15, 14]
		[90, 35, 8, 1, 5, 6, 21, 70, 48, 51] → [90, 51]
		[57, 74, 80, 40, 60, 25, 0, 52] → [57, 52]
		[44, 3, 19, 58, 50, 38, 29, 39, 2] → [44, 2]
		[56, 72, 9, 32, 7, 11, 30] → [56, 30]
0.873	c121	*remove all but last element*
	6	`(λ (x) (singleton (last x)))`
		[90, 80, 31, 14, 50] → [50]
		[11, 79, 83] → [83]
		[17, 59, 64, 22] → [22]
		[65, 43, 10, 73, 3, 51, 56, 8, 0, 2] → [2]
		[26, 24, 7, 85, 54, 52] → [52]
0.868	c045	*the input*
	2	`(λ (x) x)`
	2	`(λ (x) x)`
		[1, 1, 2, 0] → [1, 1, 2, 0]
		[0] → [0]
		[8, 8] → [8, 8]
		[] → []
		[5, 7, 9, 1, 3, 6, 4, 8, 2] → [5, 7, 9, 1, 3, 6, 4, 8, 2]

μ	ID/$\mathcal{L}$	Description, Program, & Examples
0.855	c072	*repeat every element 2 times in order of appearance*
	15	`(λ (x) (flatten (map (λ (y) (cons y (singleton y))) x)))`
	32	`(λ (x) (fix x (λ (y z) (if (is_empty z) empty (cons (head z) (cons (head z) (y (tail z))))))))`
		[1, 3, 3, 7] → [1, 1, 3, 3, 3, 3, 7, 7]
		[6, 9, 2, 8, 0, 5] → [6, 6, 9, 9, 2, 2, 8, 8, 0, 0, 5, 5]
		[9] → [9, 9]
		[4, 4, 4] → [4, 4, 4, 4, 4, 4]
		[5, 6, 4, 8, 9, 7, 3] → [5, 5, 6, 6, 4, 4, 8, 8, 9, 9, 7, 7, 3, 3]
0.853	c151	*repeat each element, M, M times, in order of appearance*
	13	`(λ (x) (flatten (map (λ (y) (repeat y y)) x)))`
		[2, 1, 3, 5, 0] → [2, 2, 1, 3, 3, 3, 5, 5, 5, 5, 5]
		[3, 4, 2, 0] → [3, 3, 3, 4, 4, 4, 4, 2, 2]
		[3] → [3, 3, 3]
		[0, 1, 1] → [1, 1]
		[1, 0] → [1]
0.841	c080	*elements in reverse order*
	4	`(λ (x) (reverse x))`
	50	`(λ (x) (fix x (λ (y z) (if (is_empty z) empty (fix (y (tail z)) (λ (u v) (if (is_empty v) (cons (head z) empty) (cons (head v) (u (tail v))))))))))`
		[0, 5, 5, 5] → [5, 5, 5, 0]
		[6, 7, 9, 1, 4, 8, 2, 0, 2, 3] → [3, 2, 0, 2, 8, 4, 1, 9, 7, 6]
		[6, 3, 1] → [1, 3, 6]
		[0, 7] → [7, 0]
		[9, 5, 3, 0, 7, 4, 7, 1, 6] → [6, 1, 7, 4, 7, 0, 3, 5, 9]
0.836	c061	*remove all but the last element*
	6	`(λ (x) (singleton (last x)))`
	22	`(λ (x) (fix x (λ (y z) (if (is_empty (tail z)) z (y (tail z))))))`
		[9, 3, 2, 4] → [4]
		[1, 7, 5, 6, 9, 8] → [8]
		[4, 3] → [3]
		[2, 0, 1] → [1]
		[6, 6] → [6]
0.833	c189	*count from the smallest element to the largest element*
	12	`(λ (x) (range (min x) 1 (max x)))`
		[2, 7, 6, 8, 4] → [2, 3, 4, 5, 6, 7, 8]
		[69, 65, 65, 65] → [65, 66, 67, 68, 69]
		[98, 98, 98, 98] → [98]
		[10, 5, 10, 9, 4, 6, 4] → [4, 5, 6, 7, 8, 9, 10]
		[0, 0, 4, 3, 1, 5, 0, 1] → [0, 1, 2, 3, 4, 5]
0.823	c050	*prepend element 1*
	8	`(λ (x) (cons (first x) x))`
	8	`(λ (x) (cons (head x) x))`
		[2, 4, 9, 3] → [2, 2, 4, 9, 3]
		[0, 4, 8, 4, 0] → [0, 0, 4, 8, 4, 0]
		[6, 6, 9, 7, 5, 9] → [6, 6, 6, 9, 7, 5, 9]
		[3, 7] → [3, 3, 7]
		[5] → [5, 5]

μ	ID/$\mathcal{L}$	Description, Program, & Examples
0.818	c048	*remove all but element 1*
	6	`(λ (x) (take 1 x))`
	8	`(λ (x) (cons (head x) empty))`
		[6, 4, 7, 9] → [6]<br>[4, 8, 6] → [4]<br>[3, 3, 3] → [3]<br>[2, 2] → [2]<br>[1, 9, 9, 5, 5] → [1]
0.811	c147	*each element, followed by its original index*
	16	`(λ (x) (flatten (mapi (λ (y z) (cons z (singleton y))) x)))`
		[88, 93, 73, 54, 79] → [88, 1, 93, 2, 73, 3, 54, 4, 79, 5]<br>[11, 0, 85, 98] → [11, 1, 0, 2, 85, 3, 98, 4]<br>[62, 53, 21] → [62, 1, 53, 2, 21, 3]<br>[90, 33] → [90, 1, 33, 2]<br>[68, 49, 92, 75, 8, 17, 40] → [68, 1, 49, 2, 92, 3, 75, 4, 8, 5, 17, 6, 40, 7]
0.806	c120	*remove all but first element*
	6	`(λ (x) (singleton (first x)))`
		[74, 1, 93, 44, 5] → [74]<br>[52, 27, 13, 3, 0, 60, 51, 80, 21] → [52]<br>[19, 54] → [19]<br>[46, 7, 84, 59, 89, 6, 2] → [46]<br>[62, 4, 98, 65, 42, 22] → [62]
0.793	c127	*remove last element*
	6	`(λ (x) (droplast 1 x))`
		[74, 12, 59, 87, 7] → [74, 12, 59, 87]<br>[9, 28, 91] → [9, 28]<br>[30, 36, 65, 95, 2, 4, 23, 93, 6, 73] → [30, 36, 65, 95, 2, 4, 23, 93, 6]<br>[45, 71, 78, 34, 3, 89, 67, 10, 96] → [45, 71, 78, 34, 3, 89, 67, 10]<br>[90, 83, 81, 1, 58, 88] → [90, 83, 81, 1, 58]
0.792	c100	*reversed input*
	4	`(λ (x) (reverse x))`
	50	`(λ (x) (fix x (λ (y z) (if (is_empty z) empty (fix (y (tail z)) (λ (u v) (if (is_empty v) (cons (head z) empty) (cons (head v) (u (tail v))))))))))`
		[31, 0, 51, 90] → [90, 51, 0, 31]<br>[6, 1, 9, 13, 70, 66, 8, 40, 7] → [7, 40, 8, 66, 70, 13, 9, 1, 6]<br>[5, 2] → [2, 5]<br>[64, 64, 97] → [97, 64, 64]<br>[75, 4, 7, 5, 33] → [33, 5, 7, 4, 75]
0.779	c145	*replace every element with element 1*
	9	`(λ (x) (map (λ (y) (first x)) x))`
		[45, 30, 33, 4, 64] → [45, 45, 45, 45, 45]<br>[70, 43, 11, 75] → [70, 70, 70, 70]<br>[51, 46, 52, 74, 5, 72, 9] → [51, 51, 51, 51, 51, 51, 51]<br>[55, 22, 7, 94, 24, 60, 79, 97, 67] → [55, 55, 55, 55, 55, 55, 55, 55, 55]<br>[54, 2, 10, 8, 6, 95] → [54, 54, 54, 54, 54, 54]

μ	ID/$\mathcal{L}$	Description, Program, & Examples
0.778	c079	*sum of elements*
	6	`(λ (x) (singleton (sum x)))`
	30	`(λ (x) (cons (fix x (λ (y z) (if (is_empty z) 0 (+ (head z) (y (tail z)))))) empty))`
		[0, 4, 1, 3] → [8]<br>[5, 0] → [5]<br>[1, 1, 7] → [9]<br>[3, 3] → [6]<br>[] → [0]
0.777	c043	*the list 8, 2, 7, 0, 3*
	20	`(λ (x) (cons 8 (cons 2 (cons 7 (cons 0 (singleton 3))))))`
	22	`(λ (x) (cons 8 (cons 2 (cons 7 (cons 0 (cons 3 empty))))))`
		[2, 2, 2, 2] → [8, 2, 7, 0, 3]<br>[5, 5, 5, 5, 5] → [8, 2, 7, 0, 3]<br>[9, 3, 7, 8, 2, 1, 9, 0] → [8, 2, 7, 0, 3]<br>[5, 0, 6, 4, 5, 5, 9, 6, 4, 1] → [8, 2, 7, 0, 3]<br>[4, 1, 6, 4, 6, 1, 6, 3, 4] → [8, 2, 7, 0, 3]
0.773	c038	*append 9*
	6	`(λ (x) (append x 9))`
	30	`(λ (x) (fix x (λ (y z) (if (is_empty z) (cons 9 empty) (cons (head z) (y (tail z)))))))`
		[4, 2, 2, 2] → [4, 2, 2, 2, 9]<br>[1, 0] → [1, 0, 9]<br>[6] → [6, 9]<br>[7, 9, 5] → [7, 9, 5, 9]<br>[8, 6, 4, 5, 1, 9, 8, 3] → [8, 6, 4, 5, 1, 9, 8, 3, 9]
0.77	c042	*the list 5, 2*
	8	`(λ (x) (cons 5 (singleton 2)))`
	10	`(λ (x) (cons 5 (cons 2 empty)))`
		[9, 3, 8, 0] → [5, 2]<br>[1, 1, 0, 7, 7] → [5, 2]<br>[6, 7, 3] → [5, 2]<br>[9, 8] → [5, 2]<br>[4, 4] → [5, 2]
0.766	c223	*swap the digits of each element*
	23	`(λ (x) (map (λ (y) (+ (* (% y 10) 10) (/ y 10))) x))`
		[4, 69, 95, 9, 49] → [40, 96, 59, 90, 94]<br>[68, 99, 24] → [86, 99, 42]<br>[54, 62, 9, 7, 32, 57, 45, 92, 47] → [45, 26, 90, 70, 23, 75, 54, 29, 74]<br>[15, 17, 25, 19] → [51, 71, 52, 91]<br>[18, 3, 6, 11, 58, 48] → [81, 30, 60, 11, 85, 84]
0.764	c137	*remove all occurrences of 3*
	6	`(λ (x) (cut_vals 3 x))`
		[8, 3, 1, 3, 3] → [8, 1]<br>[73, 19, 51, 99, 67, 5, 47, 4, 3] → [73, 19, 51, 99, 67, 5, 47, 4]<br>[5, 3, 34, 63, 38, 3] → [5, 34, 63, 38]<br>[44, 66, 3, 46, 2, 6, 88, 75] → [44, 66, 46, 2, 6, 88, 75]<br>[68, 76, 1, 3, 8, 12, 42, 0, 6, 18] → [68, 76, 1, 8, 12, 42, 0, 6, 18]

μ	ID/$\mathcal{L}$	**Description, Program, & Examples**
0.76	c238	*keep only elements that appear exactly once*
	17	`(λ (x) (filter (λ (y) (== 1 (count (== y) x))) x))`
		[8, 0, 5, 12, 0, 2] → [8, 5, 12, 2]<br>[8, 19, 7, 8, 8, 8, 7, 7, 7, 7] → [19]<br>[0, 1, 18, 9, 9, 0, 15, 6, 1] → [18, 15, 6]<br>[0, 17, 4, 8, 4, 10, 1] → [0, 17, 8, 10, 1]<br>[5, 3, 1, 6, 6, 3, 4, 4] → [5, 1]
0.758	c108	*sum of elements*
	6	`(λ (x) (singleton (sum x)))`
		[9, 6, 15, 3, 43] → [76]<br>[] → [0]<br>[12, 15, 7, 10, 8, 29] → [81]<br>[39, 23, 34] → [96]<br>[24, 46] → [70]
0.758	c126	*remove element 1*
	6	`(λ (x) (drop 1 x))`
		[39, 52, 17, 56, 10] → [52, 17, 56, 10]<br>[42, 5, 82, 8, 16, 19, 99, 3] → [5, 82, 8, 16, 19, 99, 3]<br>[80, 0, 88, 30, 7, 15, 55] → [0, 88, 30, 7, 15, 55]<br>[36, 73, 54] → [73, 54]<br>[4, 24, 81, 64, 14, 70, 13, 32, 2, 9] → [24, 81, 64, 14, 70, 13, 32, 2, 9]
0.756	c187	*concatenate input with itself, separated by 0*
	10	`(λ (x) (concat x (cons 0 x)))`
		[83, 90, 11, 35, 5] → [83, 90, 11, 35, 5, 0, 83, 90, 11, 35, 5]<br>[53, 73] → [53, 73, 0, 53, 73]<br>[77, 7, 22] → [77, 7, 22, 0, 77, 7, 22]<br>[89, 50, 2, 95] → [89, 50, 2, 95, 0, 89, 50, 2, 95]<br>[16] → [16, 0, 16]
0.752	c022	*insert a 5 as element 2*
	8	`(λ (x) (insert 5 2 x))`
	14	`(λ (x) (cons (head x) (cons 5 (tail x))))`
		[6, 5, 3, 3] → [6, 5, 5, 3, 3]<br>[8, 4, 4, 4, 8, 4] → [8, 5, 4, 4, 4, 8, 4]<br>[1, 1] → [1, 5, 1]<br>[0, 2, 6] → [0, 5, 2, 6]<br>[1, 9, 0, 9, 1] → [1, 5, 9, 0, 9, 1]
0.75	c212	*insert 3 at index 3, 3 times*
	18	`(λ (x) (splice (cons 3 (cons 3 (singleton 3))) 3 x))`
		[5, 9, 7, 80, 82] → [5, 9, 3, 3, 3, 7, 80, 82]<br>[6, 54, 74, 26, 8, 95] → [6, 54, 3, 3, 3, 74, 26, 8, 95]<br>[59, 96, 98, 25, 87, 86, 4] → [59, 96, 3, 3, 3, 98, 25, 87, 86, 4]<br>[72, 15, 39] → [72, 15, 3, 3, 3, 39]<br>[2, 65, 53, 68] → [2, 65, 3, 3, 3, 53, 68]
0.748	c101	*the list 11, 19, 24, 33, 42, 5, 82, 0, 64, 9*
	42	`(λ (x) (cons 11 (cons 19 (cons 24 (cons 33 (cons 42 (cons 5 (cons 82 (cons 0 (cons 64 (cons 9 empty)))))))))))`
		[2, 67, 32, 46, 12] → [11, 19, 24, 33, 42, 5, 82, 0, 64, 9]<br>[77, 51, 8, 27, 39, 7, 4, 92, 2, 71] → [11, 19, 24, 33, 42, 5, 82, 0, 64, 9]<br>[36, 86, 78, 66, 6, 1, 70, 72] → [11, 19, 24, 33, 42, 5, 82, 0, 64, 9]<br>[37, 47, 3, 74, 20, 20, 3] → [11, 19, 24, 33, 42, 5, 82, 0, 64, 9]<br>[31, 80, 97, 98, 85, 60] → [11, 19, 24, 33, 42, 5, 82, 0, 64, 9]

μ	ID/$\mathcal{L}$	Description, Program, & Examples
0.744	c021	*insert an 8 as element 2*
	8	`(λ (x) (insert 8 2 x))`
	14	`(λ (x) (cons (head x) (cons 8 (tail x))))`
		[1, 6, 9, 3] → [1, 8, 6, 9, 3]<br>[7, 4] → [7, 8, 4]<br>[0, 2, 3] → [0, 8, 2, 3]<br>[7, 9, 1, 2, 6, 3, 0, 4, 7, 5] → [7, 8, 9, 1, 2, 6, 3, 0, 4, 7, 5]<br>[0, 8, 6, 4, 0] → [0, 8, 8, 6, 4, 0]
0.744	c105	*concatenate all but the last element with all but the first element*
	16	`(λ (x) (splice (drop 1 (droplast 1 x)) 2 x))`
		[31, 40, 16, 44, 73] → [31, 40, 16, 44, 40, 16, 44, 73]<br>[5, 1, 10, 24] → [5, 1, 10, 1, 10, 24]<br>[26, 25, 85, 7, 94, 46] → [26, 25, 85, 7, 94, 25, 85, 7, 94, 46]<br>[45, 62, 96, 4, 79, 57] → [45, 62, 96, 4, 79, 62, 96, 4, 79, 57]<br>[6, 35, 75] → [6, 35, 35, 75]
0.74	c041	*the list 9*
	4	`(λ (x) (singleton 9))`
	6	`(λ (x) (cons 9 empty))`
		[7, 3, 6, 4] → [9]<br>[8, 7, 5, 5, 1, 6] → [9]<br>[] → [9]<br>[2, 2] → [9]<br>[0] → [9]
0.738	c070	*concatenate input with itself*
	6	`(λ (x) (concat x x))`
	26	`(λ (x) (fix x (λ (y z) (if (is_empty z) x (cons (head z) (y (tail z)))))))`
		[3, 2, 0, 9] → [3, 2, 0, 9, 3, 2, 0, 9]<br>[7, 1, 1, 2, 1, 2] → [7, 1, 1, 2, 1, 2, 7, 1, 1, 2, 1, 2]<br>[7] → [7, 7]<br>[0, 8, 4, 3, 6, 8, 4] → [0, 8, 4, 3, 6, 8, 4, 0, 8, 4, 3, 6, 8, 4]<br>[5, 5] → [5, 5, 5, 5]
0.736	c052	*repeat element 1 ten times*
	8	`(λ (x) (repeat (first x) 10))`
	62	`(λ (x) (cons (head x) (cons (head x) (cons (head x) (cons (head x) (cons (head x) (cons (head x) (cons (head x) (cons (head x) (cons (head x) (cons (head x) empty)))))))))))`
		[9, 8, 7, 1] → [9, 9, 9, 9, 9, 9, 9, 9, 9, 9]<br>[2, 5, 5] → [2, 2, 2, 2, 2, 2, 2, 2, 2, 2]<br>[4, 0, 6, 7, 3, 5, 1, 6, 3] → [4, 4, 4, 4, 4, 4, 4, 4, 4, 4]<br>[6, 7, 1] → [6, 6, 6, 6, 6, 6, 6, 6, 6, 6]<br>[0] → [0, 0, 0, 0, 0, 0, 0, 0, 0, 0]
0.723	c044	*the list 1, 9, 4, 3, 2, 5, 8, 0, 4, 9*
	40	`(λ (x) (cons 1 (cons 9 (cons 4 (cons 3 (cons 2 (cons 5 (cons 8 (cons 0 (cons 4 (singleton 9)))))))))))`
	42	`(λ (x) (cons 1 (cons 9 (cons 4 (cons 3 (cons 2 (cons 5 (cons 8 (cons 0 (cons 4 (cons 9 empty)))))))))))`
		[2, 7, 7, 3] → [1, 9, 4, 3, 2, 5, 8, 0, 4, 9]<br>[6, 6, 6] → [1, 9, 4, 3, 2, 5, 8, 0, 4, 9]<br>[0, 0, 1, 6, 1, 6, 1] → [1, 9, 4, 3, 2, 5, 8, 0, 4, 9]<br>[] → [1, 9, 4, 3, 2, 5, 8, 0, 4, 9]<br>[7] → [1, 9, 4, 3, 2, 5, 8, 0, 4, 9]

μ	ID/$\mathcal{L}$	Description, Program, & Examples
0.717	c190	*count by 2 from the first element to the last element*
	12	`(λ (x) (range (first x) 2 (last x)))`
		[46, 19, 96, 58, 58] → [46, 48, 50, 52, 54, 56, 58]<br>[2, 8, 1, 14, 7, 4, 61, 0, 6] → [2, 4, 6]<br>[66, 3, 6, 46, 82, 88, 17, 1, 93, 76] → [66, 68, 70, 72, 74, 76]<br>[8, 92, 0, 2, 94, 41, 14] → [8, 10, 12, 14]<br>[24, 4, 32] → [24, 26, 28, 30, 32]
0.717	c037	*append 3*
	6	`(λ (x) (append x 3))`
	30	`(λ (x) (fix x (λ (y z) (if (is_empty z) (cons 3 empty) (cons (head z) (y (tail z)))))))`
		[2, 0, 6, 0] → [2, 0, 6, 0, 3]<br>[9, 9] → [9, 9, 3]<br>[3, 1, 7] → [3, 1, 7, 3]<br>[4, 4, 5, 5, 4, 4, 5, 4] → [4, 4, 5, 5, 4, 4, 5, 4, 3]<br>[2, 6, 9, 6, 7, 1] → [2, 6, 9, 6, 7, 1, 3]
0.716	c222	*replace each element with the input length*
	9	`(λ (x) (map (λ (y) (length x)) x))`
		[34, 83, 11, 82, 31] → [5, 5, 5, 5, 5]<br>[74, 59, 14, 50] → [4, 4, 4, 4]<br>[25] → [1]<br>[58, 80] → [2, 2]<br>[22, 92, 28] → [3, 3, 3]
0.714	c107	*unique elements*
	4	`(λ (x) (unique x))`
		[87, 87, 17, 17, 17, 87] → [87, 17]<br>[3, 92, 18, 6, 49, 49, 1, 38, 80] → [3, 92, 18, 6, 49, 1, 38, 80]<br>[68, 68, 68, 68, 68, 68, 68, 68, 68, 68] → [68]<br>[89, 89, 89, 36, 55, 14, 7, 14] → [89, 36, 55, 14, 7]<br>[81, 69, 85, 81, 69, 74, 0, 24, 74, 61] → [81, 69, 85, 74, 0, 24, 61]
0.713	c011	*elements 2 through 4*
	8	`(λ (x) (slice 2 4 x))`
	32	`(λ (x) (cons (head (tail x)) (cons (head (tail (tail x))) (cons (head (tail (tail (tail x)))) empty))))`
		[6, 1, 3, 0, 4, 9] → [1, 3, 0]<br>[7, 2, 4, 2, 4, 7, 2, 2, 4] → [2, 4, 2]<br>[6, 8, 6, 9, 9, 8, 8, 6] → [8, 6, 9]<br>[1, 1, 4, 0, 3, 1, 3, 5, 0] → [1, 4, 0]<br>[5, 8, 5, 8, 3, 8, 7, 3, 7] → [8, 5, 8]
0.712	c104	*maximum element*
	6	`(λ (x) (singleton (max x)))`
		[5, 26, 31, 76, 89] → [89]<br>[9, 42, 54, 18, 83, 7, 34] → [83]<br>[4, 24, 58, 93, 28, 60, 2, 0, 22, 8] → [93]<br>[48, 47] → [48]<br>[63, 46, 6] → [63]
0.706	c192	*replace each element, M, with its tens digit*
	11	`(λ (x) (map (λ (y) (/ y 10)) x))`
		[31, 14, 3, 18, 32] → [3, 1, 0, 1, 3]<br>[61, 40, 77, 2] → [6, 4, 7, 0]<br>[92, 47, 62] → [9, 4, 6]<br>[13] → [1]<br>[45, 58] → [4, 5]

μ	ID/$\mathcal{L}$	Description, Program, & Examples
0.706	c182	*keep every third element*
	16	`(λ (x) (filteri (λ (y z) (== (% y 3) 0)) x))`
		[64, 6, 85, 21, 47, 46, 60, 4, 7] → [85, 46, 7]<br>[56, 12, 39, 29, 71, 2, 1, 55, 9, 93] → [39, 2, 9]<br>[20, 92, 79, 36, 7, 34, 26, 25, 1] → [79, 34, 1]<br>[41, 67, 38, 84, 14, 80, 99, 91, 23, 8] → [38, 80, 23]<br>[42, 77, 3, 57, 5, 0, 79, 13, 83, 4] → [3, 0, 83]
0.701	c006	*the first 2 elements*
	6	`(λ (x) (take 2 x))`
	16	`(λ (x) (cons (head x) (cons (head (tail x)) empty)))`
		[7, 8, 5, 7] → [7, 8]<br>[3, 1, 1, 1, 3, 1, 1] → [3, 1]<br>[8, 0, 8, 7, 4, 0, 4] → [8, 0]<br>[2, 0, 4, 6, 5] → [2, 0]<br>[9, 9, 9] → [9, 9]
0.701	c046	*prepend 7*
	6	`(λ (x) (cons 7 x))`
	6	`(λ (x) (cons 7 x))`
		[8, 8, 5, 5] → [7, 8, 8, 5, 5]<br>[] → [7]<br>[9] → [7, 9]<br>[1, 4] → [7, 1, 4]<br>[3, 8, 6, 7, 0, 3, 4] → [7, 3, 8, 6, 7, 0, 3, 4]
0.7	c106	*elements in ascending order*
	7	`(λ (x) (sort (λ (y) y) x))`
		[36, 86, 2, 97, 10] → [2, 10, 36, 86, 97]<br>[50, 94, 0, 83, 77, 71, 5, 3, 57, 8] → [0, 3, 5, 8, 50, 57, 71, 77, 83, 94]<br>[85, 26, 7, 23, 48, 39] → [7, 23, 26, 39, 48, 85]<br>[89, 4, 21, 35, 78, 96, 11, 90, 47] → [4, 11, 21, 35, 47, 78, 89, 90, 96]<br>[68, 1, 44, 93] → [1, 44, 68, 93]
0.699	c114	*prepend the last element*
	8	`(λ (x) (cons (last x) x))`
		[86, 84, 60, 20, 21] → [21, 86, 84, 60, 20, 21]<br>[10, 4, 51, 57] → [57, 10, 4, 51, 57]<br>[67, 72] → [72, 67, 72]<br>[35, 49, 2, 45, 46, 92, 9, 6, 58] → [58, 35, 49, 2, 45, 46, 92, 9, 6, 58]<br>[55, 5, 56] → [56, 55, 5, 56]
0.694	c093	*repeat element 1 ten times*
	8	`(λ (x) (repeat (first x) 10))`
	62	`(λ (x) (cons (head x) (cons (head x) (cons (head x) (cons (head x) (cons (head x) (cons (head x) (cons (head x) (cons (head x) (cons (head x) (cons (head x) empty)))))))))))`
		[94, 36, 57, 91] → [94, 94, 94, 94, 94, 94, 94, 94, 94, 94]<br>[93, 7, 37, 90, 0, 99, 6, 6] → [93, 93, 93, 93, 93, 93, 93, 93, 93, 93]<br>[62] → [62, 62, 62, 62, 62, 62, 62, 62, 62, 62]<br>[19, 5, 2, 76, 61, 5, 43, 1, 20] → [19, 19, 19, 19, 19, 19, 19, 19, 19, 19]<br>[4, 63, 0, 58, 61, 9, 0, 1, 85, 8] → [4, 4, 4, 4, 4, 4, 4, 4, 4, 4]

μ	ID/$\mathcal{L}$	Description, Program, & Examples
0.693	c195	*element 1, followed by 23, 68, 42, 99, 71, followed by last element*
	32	`(λ (x) (cons (first x) (cons 23 (cons 68 (cons 42 (cons 99 (cons 71 (singleton (last x)))))))))`
		[1, 72, 24, 78, 31] → [1, 23, 68, 42, 99, 71, 31]
		[6, 61, 57, 53, 51, 95, 26] → [6, 23, 68, 42, 99, 71, 26]
		[79, 7, 54, 1, 38, 84] → [79, 23, 68, 42, 99, 71, 84]
		[64, 41, 89, 75, 63, 40, 2, 43, 21, 9] → [64, 23, 68, 42, 99, 71, 9]
		[56, 55, 17, 46, 94, 50, 29, 5, 0] → [56, 23, 68, 42, 99, 71, 0]
0.693	c196	*concatenate 17, 38, 82, input, and 1, 55, 27*
	30	`(λ (x) (concat (cons 17 (cons 38 (singleton 82))) (concat x (cons 1 (cons 55 (singleton 27))))))`
		[90, 0, 19, 94, 8] → [17, 38, 82, 90, 0, 19, 94, 8, 1, 55, 27]
		[24, 49, 53] → [17, 38, 82, 24, 49, 53, 1, 55, 27]
		[86, 77] → [17, 38, 82, 86, 77, 1, 55, 27]
		[51, 52, 91, 9] → [17, 38, 82, 51, 52, 91, 9, 1, 55, 27]
		[15] → [17, 38, 82, 15, 1, 55, 27]
0.692	c095	*remove the first and last elements*
	10	`(λ (x) (drop 1 (droplast 1 x)))`
	30	`(λ (x) (fix (tail x) (λ (y z) (if (is_empty (tail z)) empty (cons (head z) (y (tail z)))))))`
		[8, 97, 65, 9, 54, 97] → [97, 65, 9, 54]
		[16, 51, 51, 16, 16, 0, 0, 85, 51, 9] → [51, 51, 16, 16, 0, 0, 85, 51]
		[6, 21, 6, 59, 6, 59, 6, 12, 12] → [21, 6, 59, 6, 59, 6, 12]
		[56, 39, 5, 5, 2, 24, 24] → [39, 5, 5, 2, 24]
		[46, 46, 46, 46, 32, 32, 32, 46] → [46, 46, 46, 32, 32, 32]
0.689	c016	*replace element 2 with an 8*
	8	`(λ (x) (replace 2 8 x))`
	16	`(λ (x) (cons (head x) (cons 8 (tail (tail x)))))`
		[1, 1, 0, 2] → [1, 8, 0, 2]
		[6, 1, 2, 4, 3, 5, 3, 9, 0, 7] → [6, 8, 2, 4, 3, 5, 3, 9, 0, 7]
		[5, 5, 5] → [5, 8, 5]
		[8, 1, 9, 6, 0, 7, 5] → [8, 8, 9, 6, 0, 7, 5]
		[9, 2] → [9, 8]
0.687	c224	*the last element, followed by element 1, followed by the second to last element, followed by element 2, and so on*
	16	`(λ (x) (fold (λ (y z) (cons z (reverse y))) empty x))`
		[80, 31, 6, 69, 38] → [38, 6, 80, 31, 69]
		[29, 17, 49, 99, 41, 93, 0, 2, 5, 3] → [3, 2, 93, 99, 17, 29, 49, 41, 0, 5]
		[68, 4, 34, 17, 24, 85, 82, 7, 52] → [52, 82, 24, 34, 68, 4, 17, 85, 7]
		[87, 73, 92, 8] → [8, 73, 87, 92]
		[10, 65, 16, 45, 97, 22, 30] → [30, 97, 16, 10, 65, 45, 22]
0.682	c116	*reflect the input on itself*
	12	`(λ (x) (concat (reverse (drop 1 x)) x))`
		[52, 72, 4, 18, 70] → [70, 18, 4, 72, 52, 72, 4, 18, 70]
		[48, 47, 27] → [27, 47, 48, 47, 27]
		[67, 23, 25, 54] → [54, 25, 23, 67, 23, 25, 54]
		[31, 2, 68, 11, 5, 65, 81, 28] → [28, 81, 65, 5, 11, 68, 2, 31, 2, 68, 11, 5, 65, 81, 28]
		[64, 66] → [66, 64, 66]

μ	ID/$\mathcal{L}$	Description, Program, & Examples
0.682	c140	*replace element 2 with a 9*
	8	`(λ (x) (replace 2 9 x))`
		[75, 78, 54, 76, 56] → [75, 9, 54, 76, 56]
		[35, 24, 0, 8, 51, 42, 60, 20, 4] → [35, 9, 0, 8, 51, 42, 60, 20, 4]
		[16, 31, 77, 74, 38, 23] → [16, 9, 77, 74, 38, 23]
		[7, 2, 0, 6, 67, 64, 5, 30, 95, 70] → [7, 9, 0, 6, 67, 64, 5, 30, 95, 70]
		[25, 48, 96, 89] → [25, 9, 96, 89]
0.679	c049	*remove element 1*
	6	`(λ (x) (drop 1 x))`
	4	`(λ (x) (tail x))`
		[3, 3, 3, 3] → [3, 3, 3]
		[7, 1, 4, 1, 0, 8] → [1, 4, 1, 0, 8]
		[1, 0, 9, 0, 2] → [0, 9, 0, 2]
		[4, 9, 7, 6, 6, 4, 5] → [9, 7, 6, 6, 4, 5]
		[2] → []
0.678	c071	*add 2 to every element*
	11	`(λ (x) (map (λ (y) (+ 2 y)) x))`
	30	`(λ (x) (fix x (λ (y z) (if (is_empty z) empty (cons (+ 2 (head z)) (y (tail z)))))))`
		[0, 0, 7, 0] → [2, 2, 9, 2]
		[6, 7, 7, 6, 1, 4, 2, 6, 5] → [8, 9, 9, 8, 3, 6, 4, 8, 7]
		[6] → [8]
		[5, 5] → [7, 7]
		[1, 4, 3, 6, 0] → [3, 6, 5, 8, 2]
0.678	c161	*replace each element, M, with M + the input length - M's index*
	18	`(λ (x) (mapi (λ (y z) (+ z (- (length x) y))) x))`
		[75, 25, 38, 55, 91, 26] → [80, 29, 41, 57, 92, 26]
		[5, 30, 2, 9, 3, 19, 92, 15] → [12, 36, 7, 13, 6, 21, 93, 15]
		[38, 10, 66, 49, 50, 8, 61, 59, 64] → [46, 17, 72, 54, 54, 11, 63, 60, 64]
		[11, 19, 0, 31, 40, 16, 78] → [17, 24, 4, 34, 42, 17, 78]
		[89, 4, 7, 8, 82, 3, 9, 45, 38, 94] → [98, 12, 14, 14, 87, 7, 12, 47, 39, 94]
0.675	c068	*concatenate input and 7, 3, 8, 4, 3*
	24	`(λ (x) (concat x (cons 7 (cons 3 (cons 8 (cons 4 (singleton 3)))))))`
	46	`(λ (x) (fix x (λ (y z) (if (is_empty z) (cons 7 (cons 3 (cons 8 (cons 4 (cons 3 empty))))) (cons (head z) (y (tail z)))))))`
		[8, 0, 8, 0] → [8, 0, 8, 0, 7, 3, 8, 4, 3]
		[5, 5, 4, 7, 4, 7, 5, 4] → [5, 5, 4, 7, 4, 7, 5, 4, 7, 3, 8, 4, 3]
		[0] → [0, 7, 3, 8, 4, 3]
		[] → [7, 3, 8, 4, 3]
		[6, 2, 1, 6, 2, 1] → [6, 2, 1, 6, 2, 1, 7, 3, 8, 4, 3]
0.675	c103	*input length*
	6	`(λ (x) (singleton (length x)))`
		[38, 51, 18, 72, 13] → [5]
		[] → [0]
		[83] → [1]
		[73, 91, 96, 60, 61, 42, 2, 6, 33] → [9]
		[90, 93, 81, 3, 57, 69, 21] → [7]

μ	ID/$\mathcal{L}$	Description, Program, & Examples
0.673	c001	*remove all but element 3*
	6	`(λ (x) (singleton (third x)))`
	12	`(λ (x) (cons (head (tail (tail x))) empty))`
		[2, 4, 3, 2] → [3]<br>[9, 6, 9, 8, 6] → [9]<br>[0, 0, 0, 0, 0] → [0]<br>[8, 1, 8] → [8]<br>[5, 7, 5, 7, 5] → [5]
0.67	c090	*the list 18, 42, 77, 20, 36*
	20	`(λ (x) (cons 18 (cons 42 (cons 77 (cons 20 (singleton 36))))))`
	22	`(λ (x) (cons 18 (cons 42 (cons 77 (cons 20 (cons 36 empty))))))`
		[79, 85, 85, 85] → [18, 42, 77, 20, 36]<br>[33, 33] → [18, 42, 77, 20, 36]<br>[92, 2, 2, 7, 9, 84, 52, 5, 6, 41] → [18, 42, 77, 20, 36]<br>[2, 34, 96, 49, 83, 41, 35, 4, 39, 97] → [18, 42, 77, 20, 36]<br>[89, 68, 4, 3, 68, 76, 80, 6] → [18, 42, 77, 20, 36]
0.667	c097	*concatenate 11, 21, 43, 19, input, and 7, 89, 0, 57*
	38	`(λ (x) (concat (cons 11 (cons 21 (cons 43 (singleton 19)))) (concat x (cons 7 (cons 89 (cons 0 (singleton 57)))))))`
	58	`(λ (x) (cons 11 (cons 21 (cons 43 (cons 19 (fix x (λ (y z) (if (is_empty z) (cons 7 (cons 89 (cons 0 (cons 57 empty)))) (cons (head z) (y (tail z))))))))))`
		[6, 59, 33, 33] → [11, 21, 43, 19, 6, 59, 33, 33, 7, 89, 0, 57]<br>[2, 87, 9, 99, 62, 4] → [11, 21, 43, 19, 2, 87, 9, 99, 62, 4, 7, 89, 0, 57]<br>[39, 87] → [11, 21, 43, 19, 39, 87, 7, 89, 0, 57]<br>[91] → [11, 21, 43, 19, 91, 7, 89, 0, 57]<br>[] → [11, 21, 43, 19, 7, 89, 0, 57]
0.665	c142	*every digit in order of appearance*
	23	`(λ (x) (flatten (map (λ (y) (cons (/ y 10) (singleton (% y 10)))) x)))`
		[77, 63, 83, 97, 58] → [7, 7, 6, 3, 8, 3, 9, 7, 5, 8]<br>[47, 54] → [4, 7, 5, 4]<br>[95, 60, 15, 52] → [9, 5, 6, 0, 1, 5, 5, 2]<br>[33] → [3, 3]<br>[70, 2, 92, 13, 22, 19] → [7, 0, 0, 2, 9, 2, 1, 3, 2, 2, 1, 9]
0.661	c091	*the list 81, 99, 41, 23, 22, 75, 68, 30, 24, 69*
	40	`(λ (x) (cons 81 (cons 99 (cons 41 (cons 23 (cons 22 (cons 75 (cons 68 (cons 30 (cons 24 (singleton 69)))))))))))`
	42	`(λ (x) (cons 81 (cons 99 (cons 41 (cons 23 (cons 22 (cons 75 (cons 68 (cons 30 (cons 24 (cons 69 empty)))))))))))`
		[3, 88, 88] → [81, 99, 41, 23, 22, 75, 68, 30, 24, 69]<br>[] → [81, 99, 41, 23, 22, 75, 68, 30, 24, 69]<br>[6] → [81, 99, 41, 23, 22, 75, 68, 30, 24, 69]<br>[61, 1, 59, 4, 5, 35, 48, 27, 9] → [81, 99, 41, 23, 22, 75, 68, 30, 24, 69]<br>[72, 58, 56, 49, 40, 7, 25, 1] → [81, 99, 41, 23, 22, 75, 68, 30, 24, 69]
0.659	c034	*swap elements 2 and 3*
	8	`(λ (x) (swap 2 3 x))`
	32	`(λ (x) (cons (head x) (cons (head (tail (tail x))) (cons (head (tail x)) (tail (tail (tail x)))))))`
		[1, 9, 1, 4] → [1, 1, 9, 4]<br>[7, 1, 8, 6, 3, 5, 9, 4] → [7, 8, 1, 6, 3, 5, 9, 4]<br>[6, 1, 2, 7, 4] → [6, 2, 1, 7, 4]<br>[9, 0, 2] → [9, 2, 0]<br>[3, 1, 4, 2, 6, 5, 9, 7, 0, 8] → [3, 4, 1, 2, 6, 5, 9, 7, 0, 8]

μ	ID/$\mathcal{L}$	Description, Program, & Examples
0.657	c244	*number of 3s*
	13	`(λ (x) (singleton (count (λ (y) (== 3 y)) x)))`
		[52, 3, 3, 3, 3, 3, 52] → [5]<br>[3, 3, 3, 3, 3, 14, 14, 3, 14] → [6]<br>[28, 79, 1, 3, 55, 42, 70, 60, 7, 67] → [1]<br>[92, 11, 94, 7, 2, 1, 18, 8, 89, 5] → [0]<br>[3, 43, 3, 3, 27, 3, 7, 69] → [4]
0.655	c171	*cumulative sum of elements*
	26	`(λ (x) (drop 1 (fold (λ (y z) (append y (+ (last y) z))) (singleton 0) x)))`
		[2, 9, 17, 9, 17, 4] → [2, 11, 28, 37, 54, 58]<br>[7, 7, 8, 3, 4, 4, 5, 0] → [7, 14, 22, 25, 29, 33, 38, 38]<br>[5, 7, 2, 6, 1, 0, 9] → [5, 12, 14, 20, 21, 21, 30]<br>[5, 0, 4, 15, 5, 7, 6, 15, 2, 7] → [5, 5, 9, 24, 29, 36, 42, 57, 59, 66]<br>[7, 7, 2, 7, 11, 0, 16, 16, 7] → [7, 14, 16, 23, 34, 34, 50, 66, 73]
0.651	c172	*cumulative product of elements*
	26	`(λ (x) (drop 1 (fold (λ (y z) (append y (* (last y) z))) (singleton 1) x)))`
		[4, 2, 2, 2] → [4, 8, 16, 32]<br>[5, 2, 7] → [5, 10, 70]<br>[4, 1, 1, 4, 1, 4, 1] → [4, 4, 4, 16, 16, 64, 64]<br>[7, 9] → [7, 63]<br>[2, 2, 2, 3, 3, 1] → [2, 4, 8, 24, 72, 72]
0.65	c062	*remove the last element*
	6	`(λ (x) (droplast 1 x))`
	28	`(λ (x) (fix x (λ (y z) (if (is_empty (tail z)) empty (cons (head z) (y (tail z)))))))`
		[2, 5, 2, 7] → [2, 5, 2]<br>[8] → []<br>[7, 6, 0, 7, 3] → [7, 6, 0, 7]<br>[9, 9] → [9]<br>[1, 3, 8, 5, 7, 6, 0, 9, 2, 4] → [1, 3, 8, 5, 7, 6, 0, 9, 2]
0.647	c096	*prepend 98 and append 37*
	10	`(λ (x) (cons 98 (append x 37)))`
	34	`(λ (x) (cons 98 (fix x (λ (y z) (if (is_empty z) (cons 37 empty) (cons (head z) (y (tail z))))))))`
		[20, 70, 38, 80] → [98, 20, 70, 38, 80, 37]<br>[3, 3] → [98, 3, 3, 37]<br>[] → [98, 37]<br>[8, 8, 1, 89, 85, 7, 49] → [98, 8, 8, 1, 89, 85, 7, 49, 37]<br>[6] → [98, 6, 37]
0.644	c081	*remove all but element 3*
	6	`(λ (x) (singleton (third x)))`
	12	`(λ (x) (cons (head (tail (tail x))) empty))`
		[40, 50, 76, 47, 39] → [76]<br>[9, 81, 6, 81, 6] → [6]<br>[9, 91, 70, 48, 59, 83, 43] → [70]<br>[27, 4, 38, 83, 5, 3, 15, 4, 5, 83] → [38]<br>[45, 45, 45] → [45]

μ	ID/$\mathcal{L}$	Description, Program, & Examples
0.641	c109	*product of elements*
	6	`(λ (x) (singleton (product x)))`
		[5, 1, 1, 1, 3] → [15]<br>[6, 6] → [36]<br>[] → [1]<br>[4, 3, 2, 3] → [72]<br>[7, 14, 1] → [98]
0.636	c002	*remove all but element 3*
	18	`(λ (x) (if (> 3 (length x)) empty (singleton (third x))))`
	24	`(λ (x) (if (is_empty (tail (tail x))) empty (cons (head (tail (tail x))) empty)))`
		[0, 5] → []<br>[5, 6, 1, 3, 2, 0, 7, 8, 9, 4] → [1]<br>[6, 7, 8, 1, 4, 3, 0, 5, 9, 2] → [8]<br>[7, 4, 3, 9, 5, 8, 2, 1, 6] → [3]<br>[4, 6] → []
0.636	c132	*remove element 3*
	6	`(λ (x) (cut_idx 3 x))`
		[8, 0, 3, 56, 95] → [8, 0, 56, 95]<br>[93, 5, 51, 24, 11, 7, 44] → [93, 5, 24, 11, 7, 44]<br>[27, 20, 19, 63, 13, 64] → [27, 20, 63, 13, 64]<br>[15, 57, 70, 35] → [15, 57, 35]<br>[1, 43, 23, 65, 4, 6, 28, 2, 10, 40] → [1, 43, 65, 4, 6, 28, 2, 10, 40]
0.636	c153	*each unique element followed by its number of occurrences, in order of appearance*
	24	`(λ (x) (flatten (map (λ (y) (append (take 1 y) (length y))) (group (λ (z) z) x))))`
		[23, 23, 23, 27, 27] → [23, 3, 27, 2]<br>[1, 95, 95, 41, 41, 1, 35, 95, 35] → [1, 2, 95, 3, 41, 2, 35, 2]<br>[39, 39] → [39, 2]<br>[0, 0, 97, 97, 25, 25] → [0, 2, 97, 2, 25, 2]<br>[78, 99, 50, 5, 90, 5, 78, 90, 50, 99] → [78, 2, 99, 2, 50, 2, 5, 2, 90, 2]
0.63	c051	*prepend element 1 five times*
	12	`(λ (x) (concat (repeat (first x) 5) x))`
	32	`(λ (x) (cons (head x) (cons (head x) (cons (head x) (cons (head x) (cons (head x) x))))))`
		[9, 9, 9, 9] → [9, 9, 9, 9, 9, 9, 9, 9, 9]<br>[4, 1] → [4, 4, 4, 4, 4, 4, 1]<br>[5, 7, 0, 7, 3, 5, 8] → [5, 5, 5, 5, 5, 5, 7, 0, 7, 3, 5, 8]<br>[8] → [8, 8, 8, 8, 8, 8]<br>[2, 4, 5, 1, 3, 6, 8, 0] → [2, 2, 2, 2, 2, 2, 4, 5, 1, 3, 6, 8, 0]
0.63	c225	*remove first and last two elements*
	10	`(λ (x) (drop 2 (droplast 2 x)))`
		[8, 28, 97, 66, 46] → [97]<br>[53, 95, 39, 49, 62, 74, 5, 4] → [39, 49, 62, 74]<br>[11, 87, 44, 41, 6, 27] → [44, 41]<br>[2, 40, 29, 81, 54, 48, 76, 15, 8, 80] → [29, 81, 54, 48, 76, 15]<br>[13, 38, 91, 64, 16, 0, 5] → [91, 64, 16]

μ	ID/$\mathcal{L}$	Description, Program, & Examples
0.629	c067	*swap the first and last elements*
	22	`(λ (x) (cons (last x) (append (drop 1 (droplast 1 x)) (first x))))`
	62	`(λ (x) (cons (fix x (λ (y z) (if (is_empty (tail z)) (head z) (y (tail z))))) (fix (tail x) (λ (u v) (if (is_empty (tail v)) (cons (head x) empty) (cons (head v) (u (tail v))))))))`
		[4, 8, 9, 9] → [9, 8, 9, 4]<br>[5, 0, 7, 6, 6, 0, 6, 0] → [0, 0, 7, 6, 6, 0, 6, 5]<br>[4, 7] → [7, 4]<br>[2, 1, 6, 3, 4, 0, 9, 8, 7, 4] → [4, 1, 6, 3, 4, 0, 9, 8, 7, 2]<br>[1, 3, 2, 8, 8, 5, 5] → [5, 3, 2, 8, 8, 5, 1]
0.626	c112	*count from 1 to the last element*
	10	`(λ (x) (range 1 1 (last x)))`
		[25, 0, 22, 48, 7] → [1, 2, 3, 4, 5, 6, 7]<br>[2, 66, 71, 42, 29, 99, 95, 81, 19, 3] → [1, 2, 3]<br>[5, 26, 75, 4, 97, 32, 73, 59, 1] → [1]<br>[9, 10] → [1, 2, 3, 4, 5, 6, 7, 8, 9, 10]<br>[76, 61, 15, 74, 77, 6, 2] → [1, 2]
0.624	c030	*remove the last two elements*
	6	`(λ (x) (droplast 2 x))`
	30	`(λ (x) (fix x (λ (y z) (if (is_empty (tail (tail z))) empty (cons (head z) (y (tail z)))))))`
		[6, 4, 8, 1, 0] → [6, 4, 8]<br>[9, 7, 4, 3, 8, 1] → [9, 7, 4, 3]<br>[2, 5, 7, 8, 2, 2, 9, 3, 1, 0] → [2, 5, 7, 8, 2, 2, 9, 3]<br>[5, 4, 5, 0] → [5, 4]<br>[6, 2, 9, 7, 8, 3, 8, 5, 0] → [6, 2, 9, 7, 8, 3, 8]
0.622	c149	*replace each element, M, with M * element 1; remove element 1*
	17	`(λ (x) (map (λ (y) (* y (first x))) (drop 1 x)))`
		[5, 2, 13, 6, 3] → [10, 65, 30, 15]<br>[3, 33] → [99]<br>[9] → []<br>[8, 2, 6, 9] → [16, 48, 72]<br>[6, 3, 1] → [18, 6]
0.615	c148	*count from 1 to each original element, in order of appearance*
	12	`(λ (x) (flatten (map (range 1 1) x)))`
		[2, 5, 1, 4, 1] → [1, 2, 1, 2, 3, 4, 5, 1, 1, 2, 3, 4, 1]<br>[3, 3, 2] → [1, 2, 3, 1, 2, 3, 1, 2]<br>[5, 4] → [1, 2, 3, 4, 5, 1, 2, 3, 4]<br>[3, 0, 5, 2] → [1, 2, 3, 1, 2, 3, 4, 5, 1, 2]<br>[5] → [1, 2, 3, 4, 5]
0.609	c017	*replace element 2 with an 8 if there is an element 2*
	8	`(λ (x) (replace 2 8 x))`
	26	`(λ (x) (if (is_empty (tail x)) x (cons (head x) (cons 8 (tail (tail x))))))`
		[9, 1, 7, 7] → [9, 8, 7, 7]<br>[9, 2, 1, 6, 4, 0] → [9, 8, 1, 6, 4, 0]<br>[0] → [0]<br>[1, 4, 2, 5, 3, 9, 7, 2] → [1, 8, 2, 5, 3, 9, 7, 2]<br>[0, 2, 2] → [0, 8, 2]

μ	ID/$\mathcal{L}$	Description, Program, & Examples
0.604	c092	*concatenate 92, 63, 34, 18, 55 with input*
	22	`(λ (x) (cons 92 (cons 63 (cons 34 (cons 18 (cons 55 x))))))`
	22	`(λ (x) (cons 92 (cons 63 (cons 34 (cons 18 (cons 55 x))))))`
		[97, 45, 97, 8] → [92, 63, 34, 18, 55, 97, 45, 97, 8]
		[7, 87, 87, 87, 5, 11] → [92, 63, 34, 18, 55, 7, 87, 87, 87, 5, 11]
		[8, 54, 84, 7, 9, 94, 3, 40, 6] → [92, 63, 34, 18, 55, 8, 54, 84, 7, 9, 94, 3, 40, 6]
		[1] → [92, 63, 34, 18, 55, 1]
		[66, 66] → [92, 63, 34, 18, 55, 66, 66]
0.601	c077	*input length*
	6	`(λ (x) (singleton (length x)))`
	28	`(λ (x) (cons (fix x (λ (y z) (if (is_empty z) 0 (+ 1 (y (tail z)))))) empty))`
		[1, 7, 2, 0] → [4]
		[8, 6, 6] → [3]
		[2] → [1]
		[8, 3, 9, 5, 7] → [5]
		[] → [0]
0.6	c098	*add the index to every element*
	6	`(λ (x) (mapi + x))`
	52	`(λ (x) (fix (cons 1 x) (λ (y z) (if (is_empty (tail z)) empty (cons (+ (head z) (head (tail z))) (y (cons (+ 1 (head z)) (tail (tail z)))))))))`
		[40, 52, 40, 50] → [41, 54, 43, 54]
		[0, 0, 8, 8, 8, 8] → [1, 2, 11, 12, 13, 14]
		[62, 4, 8, 85, 68, 9, 62, 85] → [63, 6, 11, 89, 73, 15, 69, 93]
		[18, 5, 79, 21, 0, 47, 91] → [19, 7, 82, 25, 5, 53, 98]
		[87, 56, 7, 56, 72, 33, 36, 57, 87, 7] → [88, 58, 10, 60, 77, 39, 43, 65, 96, 17]
0.6	c248	*elements after the last 0*
	42	`(λ (x) (first (fold (λ (y z) (if (== z 0) (cons empty y) (cons (append (first y) z) (drop 1 y)))) (singleton empty) x)))`
		[9, 0, 4, 8, 0, 2, 46, 96, 30] → [2, 46, 96, 30]
		[97, 0, 46, 8, 89, 0, 17, 3, 17, 7] → [17, 3, 17, 7]
		[0, 1, 5, 1, 32, 5, 41, 5, 0, 87] → [87]
		[70, 6, 11, 0, 37, 9, 9, 0, 13, 54] → [13, 54]
		[86, 7, 99, 0, 99, 0, 3, 4, 38] → [3, 4, 38]
0.599	c199	*elements in ascending order, each preceded by its rank*
	21	`(λ (x) (flatten (zip (range 1 1 (length x)) (sort (λ (y) y) x))))`
		[59, 22, 86, 64, 25] → [1, 22, 2, 25, 3, 59, 4, 64, 5, 86]
		[72, 69, 74, 27] → [1, 27, 2, 69, 3, 72, 4, 74]
		[6, 99, 46, 0, 96, 49, 77] → [1, 0, 2, 6, 3, 46, 4, 49, 5, 77, 6, 96, 7, 99]
		[12, 81] → [1, 12, 2, 81]
		[42] → [1, 42]
0.597	c007	*remove all but the first 2 elements*
	6	`(λ (x) (take 2 x))`
	26	`(λ (x) (if (is_empty (tail x)) x (cons (head x) (cons (head (tail x)) empty))))`
		[0, 2, 2, 0] → [0, 2]
		[] → []
		[1] → [1]
		[3, 3, 1, 9, 8] → [3, 3]
		[7, 8, 5] → [7, 8]

μ	ID/$\mathcal{L}$	Description, Program, & Examples
0.591	c176	*sums of each consecutive pair of elements, in order of appearance*
	21	`(λ (x) (map (λ (y) (sum y)) (zip (droplast 1 x) (drop 1 x))))`
		[22, 1, 6, 8, 51, 26] → [23, 7, 14, 59, 77]<br>[8, 2, 65, 9, 81, 16, 79, 3, 80, 5] → [10, 67, 74, 90, 97, 95, 82, 83, 85]<br>[8, 5, 28, 36, 58, 40, 0] → [13, 33, 64, 94, 98, 40]<br>[31, 29, 3, 19, 5, 50, 0, 76] → [60, 32, 22, 24, 55, 50, 76]<br>[20, 71, 5, 1, 38, 4, 93, 2, 50] → [91, 76, 6, 39, 42, 97, 95, 52]
0.588	c066	*left - rotate by 1*
	12	`(λ (x) (append (drop 1 x) (first x)))`
	34	`(λ (x) (fix (tail x) (λ (y z) (if (is_empty z) (cons (head x) empty) (cons (head z) (y (tail z)))))))`
		[5, 6, 5, 8] → [6, 5, 8, 5]<br>[1, 6, 6] → [6, 6, 1]<br>[8, 2, 4, 7, 3, 0] → [2, 4, 7, 3, 0, 8]<br>[7, 9, 8, 2, 5, 1, 2, 4] → [9, 8, 2, 5, 1, 2, 4, 7]<br>[3, 7] → [7, 3]
0.587	c053	*replace element 2 with element 1*
	16	`(λ (x) (concat (repeat (first x) 2) (drop 2 x)))`
	18	`(λ (x) (cons (head x) (cons (head x) (tail (tail x)))))`
		[8, 9, 6, 4] → [8, 8, 6, 4]<br>[6, 5, 8, 9, 1, 3, 4, 1, 0] → [6, 6, 8, 9, 1, 3, 4, 1, 0]<br>[9, 3, 7, 0, 1, 5, 5, 0] → [9, 9, 7, 0, 1, 5, 5, 0]<br>[5] → [5, 5]<br>[2, 0, 2, 0, 2] → [2, 2, 2, 0, 2]
0.584	c047	*concatenate 9, 6, 3, 8, 5 and input*
	22	`(λ (x) (cons 9 (cons 6 (cons 3 (cons 8 (cons 5 x))))))`
	22	`(λ (x) (cons 9 (cons 6 (cons 3 (cons 8 (cons 5 x))))))`
		[8, 3, 7, 9] → [9, 6, 3, 8, 5, 8, 3, 7, 9]<br>[4, 6, 7, 0, 7, 7, 1, 9] → [9, 6, 3, 8, 5, 4, 6, 7, 0, 7, 7, 1, 9]<br>[6] → [9, 6, 3, 8, 5, 6]<br>[] → [9, 6, 3, 8, 5]<br>[5, 2, 0, 2, 4, 0, 3, 2, 4] → [9, 6, 3, 8, 5, 5, 2, 0, 2, 4, 0, 3, 2, 4]
0.584	c064	*remove the first and last elements*
	10	`(λ (x) (drop 1 (droplast 1 x)))`
	30	`(λ (x) (fix (tail x) (λ (y z) (if (is_empty (tail z)) empty (cons (head z) (y (tail z)))))))`
		[4, 5, 0, 0] → [5, 0]<br>[3, 8, 3, 8, 3] → [8, 3, 8]<br>[4, 9, 4, 9, 4, 9, 9, 9, 9, 9] → [9, 4, 9, 4, 9, 9, 9, 9]<br>[5, 7, 7, 9, 8, 1, 4, 0, 6] → [7, 7, 9, 8, 1, 4, 0]<br>[2, 1, 1, 2, 2, 7, 2, 7] → [1, 1, 2, 2, 7, 2]
0.584	c078	*maximum element*
	6	`(λ (x) (singleton (max x)))`
	58	`(λ (x) (fix (cons 0 x) (λ (y z) (if (is_empty (tail z)) (cons (head z) empty) (y (if (> (head z) (head (tail z))) (cons (head z) (tail (tail z))) (tail z)))))))`
		[2, 3, 0, 1] → [3]<br>[2, 7, 9, 5, 4, 0, 8, 1, 3, 6] → [9]<br>[1, 0] → [1]<br>[7, 1, 5, 4, 0, 2, 8, 6] → [8]<br>[2, 5, 3, 0, 6, 4, 1] → [6]

μ	ID/$\mathcal{L}$	Description, Program, & Examples
0.584	c119	*prepend or append 8, if necessary, so the list begins and ends with 8*
	32	`(λ (x) ((λ (y) (concat (y first) (concat x (y last)))) (λ (z) (if (== (z x) 8) empty (singleton 8)))))`
		[8, 87, 23, 25, 34] → [8, 87, 23, 25, 34, 8]<br>[63] → [8, 63, 8]<br>[47, 68, 84, 8] → [8, 47, 68, 84, 8]<br>[46, 77] → [8, 46, 77, 8]<br>[52, 7, 2, 91, 5, 75, 58, 3, 8] → [8, 52, 7, 2, 91, 5, 75, 58, 3, 8]
0.573	c135	*remove the first 7*
	6	`(λ (x) (cut_val 7 x))`
		[7, 99, 63, 7, 7] → [99, 63, 7, 7]<br>[7, 7] → [7]<br>[47, 14, 45, 7, 6, 59, 48, 50, 5, 19] → [47, 14, 45, 6, 59, 48, 50, 5, 19]<br>[8, 38, 3, 42, 7, 78, 71] → [8, 38, 3, 42, 78, 71]<br>[85, 46, 84, 79, 44, 7, 1, 2, 7] → [85, 46, 84, 79, 44, 1, 2, 7]
0.571	c198	*unique elements in descending order*
	11	`(λ (x) (reverse (sort (λ (y) y) (unique x))))`
		[62, 86, 85, 62, 29, 8, 85, 29] → [86, 85, 62, 29, 8]<br>[26, 26, 26, 26, 10, 55, 6, 10, 55, 6] → [55, 26, 10, 6]<br>[18, 65, 98, 98, 98, 18, 18, 98, 65] → [98, 65, 18]<br>[7, 5, 5, 69, 69, 30, 30, 7, 5] → [69, 30, 7, 5]<br>[73, 66, 24, 52, 24, 43, 73, 24, 66, 52] → [73, 66, 52, 43, 24]
0.568	c219	*keep only every other element, starting from the end of the list*
	14	`(λ (x) (reverse (filteri (λ (y z) (is_odd y)) (reverse x))))`
		[42, 0, 46, 12, 8, 58, 50] → [42, 46, 8, 50]<br>[7, 93, 99, 86, 30, 97, 60, 62, 57, 17] → [93, 86, 97, 62, 17]<br>[3, 52, 78, 9, 94, 54, 90, 89, 37, 14] → [52, 9, 54, 89, 14]<br>[65, 2, 66, 47, 28, 71, 9, 5] → [2, 47, 71, 5]<br>[81, 22, 85, 82, 36, 59, 16, 8, 45] → [81, 85, 36, 16, 45]
0.566	c075	*remove every element with an even index*
	10	`(λ (x) (filteri (λ (y z) (is_odd y)) x))`
	28	`(λ (x) (fix x (λ (y z) (if (is_empty z) empty (cons (head z) (y (tail (tail z))))))))`
		[6, 5, 2, 9] → [6, 2]<br>[7, 3] → [7]<br>[0, 1, 1, 9, 2, 0, 1, 0, 9, 2] → [0, 1, 2, 1, 9]<br>[8, 0, 4, 2, 8] → [8, 4, 8]<br>[7, 4, 5, 2, 5, 5, 2] → [7, 5, 5, 2]
0.559	c177	*interleave the input and the reversed input*
	10	`(λ (x) (flatten (zip x (reverse x))))`
		[64, 98, 27, 26, 32] → [64, 32, 98, 26, 27, 27, 26, 98, 32, 64]<br>[1, 15, 28, 4] → [1, 4, 15, 28, 28, 15, 4, 1]<br>[80, 2, 61, 24, 37, 8] → [80, 8, 2, 37, 61, 24, 24, 61, 37, 2, 8, 80]<br>[93, 50, 81] → [93, 81, 50, 50, 81, 93]<br>[59, 90] → [59, 90, 90, 59]
0.553	c025	*remove element 2*
	6	`(λ (x) (cut_idx 2 x))`
	12	`(λ (x) (cons (head x) (tail (tail x))))`
		[1, 1, 4, 5] → [1, 4, 5]<br>[2, 6] → [2]<br>[1, 6, 6, 0, 8, 3, 9, 0, 7, 9] → [1, 6, 0, 8, 3, 9, 0, 7, 9]<br>[3, 0, 4, 9, 5] → [3, 4, 9, 5]<br>[7, 2, 5, 0, 8, 4, 1] → [7, 5, 0, 8, 4, 1]

μ	ID/$\mathcal{L}$	**Description, Program, & Examples**
0.552	c125	*keep only elements whose tens digit equals the tens digit of element 1*
	21	`(λ (x) (filter (λ (y) (== (/ (first x) 10) (/ y 10))) x))`
		[41, 6, 41, 27, 55, 66, 42, 3] → [41, 41, 42]
		[61, 62, 9, 3, 56, 85, 64, 82, 49] → [61, 62, 64]
		[5, 2, 90, 75, 57, 1, 7, 19, 8, 84] → [5, 2, 1, 7, 8]
		[32, 32, 4, 3, 32, 7, 30, 96, 5] → [32, 32, 32, 30]
		[99, 99, 97, 79, 16, 75, 75, 97, 16, 8] → [99, 99, 97, 97]
0.545	c211	*reverse input; insert elements 4 and 5 so they are fourth and third from last, respectively*
	22	`(λ (x) (splice (slice 4 5 x) (- (length x) 2) (reverse x)))`
		[22, 1, 7, 65, 21, 77] → [77, 21, 65, 65, 21, 7, 1, 22]
		[94, 72, 79, 4, 47, 46] → [46, 47, 4, 4, 47, 79, 72, 94]
		[97, 59, 0, 39, 8, 48, 53, 4] → [4, 53, 48, 8, 39, 39, 8, 0, 59, 97]
		[12, 5, 18, 62, 78, 28, 31, 68] → [68, 31, 28, 78, 62, 62, 78, 18, 5, 12]
		[49, 6, 23, 92, 45, 36, 75] → [75, 36, 45, 92, 92, 45, 23, 6, 49]
0.54	c087	*swap elements 2 and 3*
	8	`(λ (x) (swap 2 3 x))`
	32	`(λ (x) (cons (head x) (cons (head (tail (tail x))) (cons (head (tail x)) (tail (tail (tail x)))))))`
		[36, 77, 25, 3, 1] → [36, 25, 77, 3, 1]
		[21, 94, 56, 2, 0, 0, 21, 94] → [21, 56, 94, 2, 0, 0, 21, 94]
		[26, 71, 7, 31, 5, 21, 55, 4, 87] → [26, 7, 71, 31, 5, 21, 55, 4, 87]
		[72, 88, 72, 88, 45, 88, 72, 85, 45, 79] → [72, 72, 88, 88, 45, 88, 72, 85, 45, 79]
		[0, 9, 0, 68, 68, 9] → [0, 0, 9, 68, 68, 9]
0.539	c013	*elements 3 through 7*
	8	`(λ (x) (slice 3 7 x))`
	55	`(λ (x) ((λ (y) (fix (cons 5 y) (λ (z u) (if (is_equal 0 (head u)) empty (cons (head (tail u)) (z (cons (- (head u) 1) (tail (tail u))))))))) (tail (tail x))))`
		[8, 5, 5, 8, 8, 5, 5, 5] → [5, 8, 8, 5, 5]
		[9, 9, 4, 4, 9, 3, 1, 1, 9] → [4, 4, 9, 3, 1]
		[6, 4, 2, 4, 0, 0, 8, 7, 5] → [2, 4, 0, 0, 8]
		[3, 2, 0, 3, 4, 4, 6, 6] → [0, 3, 4, 4, 6]
		[9, 8, 7, 4, 1, 3, 2, 0, 6, 5] → [7, 4, 1, 3, 2]
0.534	c069	*concatenate 9, 3, 4, 0, input, and 7, 2, 9, 1*
	38	`(λ (x) (concat (cons 9 (cons 3 (cons 4 (singleton 0)))) (concat x (cons 7 (cons 2 (cons 9 (singleton 1)))))))`
	58	`(λ (x) (cons 9 (cons 3 (cons 4 (cons 0 (fix x (λ (y z) (if (is_empty z) (cons 7 (cons 2 (cons 9 (cons 1 empty)))) (cons (head z) (y (tail z)))))))))))`
		[0, 5, 5, 5] → [9, 3, 4, 0, 0, 5, 5, 5, 7, 2, 9, 1]
		[] → [9, 3, 4, 0, 7, 2, 9, 1]
		[8, 4, 7] → [9, 3, 4, 0, 8, 4, 7, 7, 2, 9, 1]
		[8] → [9, 3, 4, 0, 8, 7, 2, 9, 1]
		[7, 1] → [9, 3, 4, 0, 7, 1, 7, 2, 9, 1]
0.534	c117	*left - rotate by three elements*
	14	`(λ (x) (concat (drop 3 x) (take 3 x)))`
		[77, 90, 44, 9, 84] → [9, 84, 77, 90, 44]
		[57, 0, 17, 95, 1, 94, 68, 31, 46] → [95, 1, 94, 68, 31, 46, 57, 0, 17]
		[20, 6, 21, 56, 79, 8] → [56, 79, 8, 20, 6, 21]
		[23, 5, 42, 7, 52, 74, 25] → [7, 52, 74, 25, 23, 5, 42]
		[2, 81, 92, 80] → [80, 2, 81, 92]

μ	ID/$\mathcal{L}$	**Description, Program, & Examples**
0.531	c228	*replace each element with the number of occurrences of that element so far in the input*
	18	`(λ (x) (mapi (λ (y z) (count (== z) (take y x))) x))`
		[42, 22, 22, 42, 42] → [1, 1, 2, 2, 3]
		[11, 11, 6, 11, 11, 6, 6, 6] → [1, 2, 1, 3, 4, 2, 3, 4]
		[80, 80, 80, 80] → [1, 2, 3, 4]
		[84, 84, 84] → [1, 2, 3]
		[58, 58] → [1, 2]
0.53	c197	*replace each element with the number of occurrences of that element*
	13	`(λ (x) (map (λ (y) (count (== y) x)) x))`
		[43, 43, 19, 72, 73] → [2, 2, 1, 1, 1]
		[23, 53, 46, 79, 41, 0, 51, 41, 16, 93] → [1, 1, 1, 1, 2, 1, 1, 2, 1, 1]
		[70, 70, 70, 70, 42, 42] → [4, 4, 4, 4, 2, 2]
		[27, 74, 27, 74, 64, 74, 27, 74, 74] → [3, 5, 3, 5, 1, 5, 3, 5, 5]
		[8, 80, 80] → [1, 2, 2]
0.527	c065	*prepend 9 and append 7*
	10	`(λ (x) (cons 9 (append x 7)))`
	34	`(λ (x) (cons 9 (fix x (λ (y z) (if (is_empty z) (cons 7 empty) (cons (head z) (y (tail z))))))))`
		[1, 0, 0, 8] → [9, 1, 0, 0, 8, 7]
		[2, 2, 8] → [9, 2, 2, 8, 7]
		[] → [9, 7]
		[7] → [9, 7, 7]
		[1, 3, 1, 5, 6, 4, 4, 3, 8] → [9, 1, 3, 1, 5, 6, 4, 4, 3, 8, 7]
0.524	c156	*reverse the input and add each element to its new index*
	14	`(λ (x) (mapi (λ (y z) (+ z y)) (reverse x)))`
		[38, 61, 56, 17, 51, 4] → [5, 53, 20, 60, 66, 44]
		[28, 7, 69, 5, 55, 18, 83, 71, 46] → [47, 73, 86, 22, 60, 11, 76, 15, 37]
		[2, 33, 39, 42, 8, 7, 1, 64] → [65, 3, 10, 12, 47, 45, 40, 10]
		[37, 23, 94, 5, 27, 72, 0] → [1, 74, 30, 9, 99, 29, 44]
		[6, 48, 13, 78, 18, 88, 30, 86, 62, 21] → [22, 64, 89, 34, 93, 24, 85, 21, 57, 16]
0.523	c110	*three largest elements in ascending order*
	11	`(λ (x) (takelast 3 (sort (λ (y) y) x)))`
		[97, 7, 13, 2, 55] → [13, 55, 97]
		[18, 35, 7, 70, 96, 74, 37, 45] → [70, 74, 96]
		[45, 92, 5, 40, 3, 78, 81, 50, 4, 76] → [78, 81, 92]
		[85, 89, 8, 7, 9, 44, 30, 68, 69] → [69, 85, 89]
		[57, 15, 51, 31, 33, 61, 6] → [51, 57, 61]
0.521	c233	*number of occurrences of each unique element, in order of appearance*
	11	`(λ (x) (map length (group (λ (y) y) x)))`
		[2, 82, 82, 52, 87, 41, 87] → [1, 2, 1, 2, 1]
		[4, 63, 9, 68, 62, 67, 9, 22, 56] → [1, 1, 2, 1, 1, 1, 1, 1]
		[34, 34, 34, 34, 34, 34, 34, 34, 34, 34] → [10]
		[71, 17, 71, 71, 71, 17, 71, 17] → [5, 3]
		[33, 55, 84, 84, 64, 33, 55, 64, 6, 18] → [2, 2, 2, 2, 1, 1]
0.519	c003	*remove all but element 7*
	8	`(λ (x) (singleton (nth 7 x)))`
	20	`(λ (x) (cons (head (tail (tail (tail (tail (tail (tail x))))))) empty))`
		[7, 2, 3, 9, 0, 4, 5, 6] → [5]
		[6, 1, 6, 4, 4, 7, 0, 4, 6, 1] → [0]
		[0, 5, 7, 4, 9, 1, 3, 6, 7, 8] → [3]
		[6, 8, 5, 0, 2, 9, 8, 2] → [8]
		[1, 0, 8, 6, 7, 2, 9, 3, 5, 4] → [9]

μ	ID/$\mathcal{L}$	Description, Program, & Examples
0.519	c230	*count by 2 from the smallest element to the largest element*
	12	`(λ (x) (range (min x) 2 (max x)))`
		[8, 6, 7, 12, 2] → [2, 4, 6, 8, 10, 12]<br>[44, 48] → [44, 46, 48]<br>[5, 1, 7] → [1, 3, 5, 7]<br>[16, 6, 9, 4] → [4, 6, 8, 10, 12, 14, 16]<br>[78, 86] → [78, 80, 82, 84, 86]
0.515	c122	*remove all but penultimate element*
	8	`(λ (x) (singleton (second (reverse x))))`
		[22, 46, 27, 2, 89] → [2]<br>[77, 4, 57] → [4]<br>[80, 70] → [80]<br>[9, 26, 65, 71, 33, 5, 67, 3, 40, 56] → [40]<br>[98, 55, 32, 41, 8, 93, 11, 44, 88] → [44]
0.514	c020	*replace the first element with the last element*
	10	`(λ (x) (replace 1 (last x) x))`
	30	`(λ (x) (cons (fix x (λ (y z) (if (is_empty (tail z)) (head z) (y (tail z))))) (tail x)))`
		[7, 7, 7, 9] → [9, 7, 7, 9]<br>[8, 7, 6, 8, 9, 4, 3] → [3, 7, 6, 8, 9, 4, 3]<br>[9, 9, 2, 3, 3, 3, 2, 1, 1] → [1, 9, 2, 3, 3, 3, 2, 1, 1]<br>[8, 9, 7, 2, 7, 0] → [0, 9, 7, 2, 7, 0]<br>[8, 5] → [5, 5]
0.506	c235	*count up and down between elements*
	46	`(λ (x) (fold (λ (y z) (concat y (drop 1 (range (last y) (if (> z (last y)) 1 -1) z)))) (take 1 x) (drop 1 x)))`
		[8, 7, 5, 6, 15] → [8, 7, 6, 5, 6, 7, 8, 9, 10, 11, 12, 13, 14, 15]<br>[46, 43, 42] → [46, 45, 44, 43, 42]<br>[0, 4, 7, 6] → [0, 1, 2, 3, 4, 5, 6, 7, 6]<br>[1, 5, 7] → [1, 2, 3, 4, 5, 6, 7]<br>[6, 9] → [6, 7, 8, 9]
0.503	c157	*each element followed by 0 if even or 1 if odd, in order of appearance*
	19	`(λ (x) (flatten (map (λ (y) (cons y (singleton (% y 2)))) x)))`
		[32, 3, 5, 81, 16, 6] → [32, 0, 3, 1, 5, 1, 81, 1, 16, 0, 6, 0]<br>[63, 18, 24, 92, 44, 89, 30] → [63, 1, 18, 0, 24, 0, 92, 0, 44, 0, 89, 1, 30, 0]<br>[74, 71, 15, 46, 26, 59] → [74, 0, 71, 1, 15, 1, 46, 0, 26, 0, 59, 1]<br>[78, 94, 6, 90, 7, 77, 79] → [78, 0, 94, 0, 6, 0, 90, 0, 7, 1, 77, 1, 79, 1]<br>[96, 28, 95, 6, 4, 57, 9] → [96, 0, 28, 0, 95, 1, 6, 0, 4, 0, 57, 1, 9, 1]
0.5	c185	*replace the sublist between the first and last elements with its sum*
	26	`(λ (x) (cons (first x) (cons (sum (drop 1 (droplast 1 x))) (takelast 1 x))))`
		[41, 9, 5, 45, 30, 89] → [41, 89, 89]<br>[65, 0, 2, 23, 7, 21, 5, 3, 74] → [65, 61, 74]<br>[16, 19, 36, 12, 4, 6, 8, 2, 0, 23] → [16, 87, 23]<br>[53, 3, 1, 4, 26, 41, 35] → [53, 75, 35]<br>[81, 29, 31, 15, 7, 9, 4, 47] → [81, 95, 47]
0.5	c213	*three smallest elements in ascending order*
	11	`(λ (x) (take 3 (sort (λ (y) y) x)))`
		[16, 25, 95, 44, 39] → [16, 25, 39]<br>[58, 7, 48, 1, 43, 8, 3, 84, 53, 0] → [0, 1, 3]<br>[62, 5, 41, 7, 68, 65, 3, 32, 18] → [3, 5, 7]<br>[83, 23, 2, 52, 61, 59] → [2, 23, 52]<br>[86, 19, 21, 17, 40, 45, 13] → [13, 17, 19]

μ	ID/$\mathcal{L}$	Description, Program, & Examples
0.495	c184	*keep only odd elements with an even index*
	16	`(λ (x) (filteri (λ (y z) (and (is_even y) (is_odd z))) x))`
		[6, 91, 0, 77, 18, 25] → [91, 77, 25]
		[19, 45, 91, 1, 70, 33, 0, 81, 47, 73] → [45, 1, 33, 81, 73]
		[82, 7, 87, 9, 2, 31, 67] → [7, 9, 31]
		[54, 97, 49, 5, 6, 35, 2, 1, 78] → [97, 5, 35, 1]
		[4, 75, 8, 63, 18, 3, 51, 7] → [75, 63, 3, 7]
0.492	c155	*multiply each element by its index*
	12	`(λ (x) (mapi (λ (y z) (* z y)) x))`
		[2, 0, 6, 0, 3, 2] → [2, 0, 18, 0, 15, 12]
		[3, 7, 9, 8, 0, 6, 1, 2] → [3, 14, 27, 32, 0, 36, 7, 16]
		[5, 5, 10, 5, 6, 4, 5] → [5, 10, 30, 20, 30, 24, 35]
		[2, 4, 2, 1, 0, 2, 9, 1, 7, 6] → [2, 8, 6, 4, 0, 12, 63, 8, 63, 60]
		[9, 3, 3, 5, 8, 0, 7, 4, 2] → [9, 6, 9, 20, 40, 0, 49, 32, 18]
0.49	c082	*remove all but element 3*
	18	`(λ (x) (if (> 3 (length x)) empty (singleton (third x))))`
	24	`(λ (x) (if (is_empty (tail (tail x))) empty (cons (head (tail (tail x))) empty)))`
		[10, 6, 2, 99, 0] → [2]
		[15] → []
		[18, 79, 7, 5, 3, 7, 3, 5, 79] → [7]
		[96, 40] → []
		[91, 75, 3, 6, 8, 42, 11, 4, 1, 60] → [3]
0.487	c231	*the number of occurrences of each unique element in ascending order*
	16	`(λ (x) (sort (λ (y) y) (map length (group (λ (z) z) x))))`
		[80, 5, 98, 32, 98, 80, 98] → [1, 1, 2, 3]
		[92, 92, 92, 92, 21, 21, 92, 92] → [2, 6]
		[78, 34, 78, 78, 34, 34, 4, 4, 34, 4] → [3, 3, 4]
		[33, 31, 71, 33, 59, 64, 31, 31, 59, 7] → [1, 1, 1, 2, 2, 3]
		[38, 38, 38, 38, 38, 38, 38, 38, 38] → [9]
0.485	c099	*remove all elements whose value ¡ 50*
	11	`(λ (x) (filter (λ (y) (> y 49)) x))`
	42	`(λ (x) (fix x (λ (y z) (if (is_empty z) empty (if (> (head z) 49) (cons (head z) (y (tail z))) (y (tail z)))))))`
		[8, 87, 2, 64, 48, 79] → [87, 64, 79]
		[1, 7, 8, 25, 0, 40, 53, 94] → [53, 94]
		[78, 18, 92, 42, 95, 3, 98] → [78, 92, 95, 98]
		[7, 28, 13, 8, 0, 28, 8, 28] → []
		[86, 5, 86, 86, 11, 99, 99, 99, 5] → [86, 86, 86, 99, 99, 99]
0.485	c247	*elements before the first 0*
	44	`(λ (x) (first (reverse (fold (λ (y z) (if (== z 0) (cons empty y) (cons (append (first y) z) (drop 1 y)))) (singleton empty) x))))`
		[5, 26, 7, 23, 0, 93, 0, 18, 1] → [5, 26, 7, 23]
		[1, 71, 89, 0, 71, 46, 8, 87, 0, 7] → [1, 71, 89]
		[9, 86, 0, 60, 69, 53, 1, 0, 6, 60] → [9, 86]
		[4, 0, 80, 87, 87, 0, 45, 2, 4] → [4]
		[84, 0, 20, 0, 63, 63, 20, 20, 20] → [84]

μ	ID/$\mathcal{L}$	Description, Program, & Examples
0.476	c008	*the first 6 elements*
	6	`(λ (x) (take 6 x))`
	48	`(λ (x) (fix (cons 6 x) (λ (y z) (if (is_equal 0 (head z)) empty (cons (head (tail z)) (y (cons (- (head z) 1) (tail (tail z)))))))))`
		[1, 3, 9, 8, 1, 6, 7] → [1, 3, 9, 8, 1, 6]
		[9, 2, 2, 6, 9, 9, 2, 9] → [9, 2, 2, 6, 9, 9]
		[3, 7, 7, 0, 3, 8, 5] → [3, 7, 7, 0, 3, 8]
		[0, 4, 4, 0, 9, 0, 4] → [0, 4, 4, 0, 9, 0]
		[4, 0, 5, 6, 5, 0, 5] → [4, 0, 5, 6, 5, 0]
0.473	c055	*swap elements 1 and 3 and elements 2 and 4*
	14	`(λ (x) (swap 1 3 (swap 2 4 x)))`
	46	`(λ (x) (cons (head (tail (tail x))) (cons (head (tail (tail (tail x)))) (cons (head x) (cons (head (tail x)) (tail (tail (tail (tail x)))))))))`
		[4, 8, 7, 9] → [7, 9, 4, 8]
		[0, 2, 6, 1, 9, 5, 6, 5, 3] → [6, 1, 0, 2, 9, 5, 6, 5, 3]
		[8, 1, 7, 0, 5] → [7, 0, 8, 1, 5]
		[9, 4, 4, 3, 9, 9, 3, 4] → [4, 3, 9, 4, 9, 9, 3, 4]
		[3, 9, 7, 2, 0, 8, 5] → [7, 2, 3, 9, 0, 8, 5]
0.462	c029	*remove the first two elements*
	6	`(λ (x) (drop 2 x))`
	6	`(λ (x) (tail (tail x)))`
		[8, 1, 9, 9] → [9, 9]
		[2, 2] → []
		[5, 1, 0, 0, 5, 8, 1, 8, 3, 0] → [0, 0, 5, 8, 1, 8, 3, 0]
		[6, 6, 6] → [6]
		[7, 0, 4, 1, 7] → [4, 1, 7]
0.459	c168	*count from 1 to 10, skipping the input's length*
	14	`(λ (x) (cut_val (length x) (range 1 1 10)))`
		[66, 74, 88, 49, 15] → [1, 2, 3, 4, 6, 7, 8, 9, 10]
		[96, 25, 43, 86, 50, 44, 13, 87, 2, 84] → [1, 2, 3, 4, 5, 6, 7, 8, 9]
		[21, 85] → [1, 3, 4, 5, 6, 7, 8, 9, 10]
		[98, 30, 27, 53, 7, 45, 0] → [1, 2, 3, 4, 5, 6, 8, 9, 10]
		[65, 1, 69, 76, 33, 16] → [1, 2, 3, 4, 5, 7, 8, 9, 10]
0.45	c009	*remove all but the first 6 elements*
	6	`(λ (x) (take 6 x))`
	58	`(λ (x) (fix (cons 6 x) (λ (y z) (if (is_equal 0 (head z)) empty (if (is_empty (tail z)) empty (cons (head (tail z)) (y (cons (- (head z) 1) (tail (tail z))))))))))`
		[6, 2, 4, 4, 4, 8, 8] → [6, 2, 4, 4, 4, 8]
		[7] → [7]
		[5, 8, 8, 9, 9, 5, 8, 5] → [5, 8, 8, 9, 9, 5]
		[6, 7, 0] → [6, 7, 0]
		[1, 1, 1, 1] → [1, 1, 1, 1]
0.442	c133	*remove elements 2 through 5*
	8	`(λ (x) (cut_slice 2 5 x))`
		[17, 65, 41, 49, 9, 5] → [17, 5]
		[85, 50, 30, 14, 6, 89, 57, 77] → [85, 89, 57, 77]
		[73, 3, 2, 70, 21, 87, 86, 23, 76] → [73, 87, 86, 23, 76]
		[11, 2, 74, 41, 1, 10, 0] → [11, 10, 0]
		[31, 47, 82, 96, 52, 98, 3, 4, 68, 61] → [31, 98, 3, 4, 68, 61]

μ	ID/$\mathcal{L}$	Description, Program, & Examples
0.435	c146	*absolute difference of each consecutive pair, in order of appearance*
	27	`(λ (x) (map (λ (y) (- (max y) (min y))) (zip (droplast 1 x) (drop 1 x))))`
		[87, 67, 47, 9, 44] → [20, 20, 38, 35]<br>[80, 98, 4, 25] → [18, 94, 21]<br>[2, 53, 31, 23, 49, 6, 16] → [51, 22, 8, 26, 43, 10]<br>[58, 86, 12, 66, 90, 20, 45, 64] → [28, 74, 54, 24, 70, 25, 19]<br>[8, 34, 17, 82, 4, 93, 5, 18, 41, 11] → [26, 17, 65, 78, 89, 88, 13, 23, 30]
0.434	c004	*remove all but element 7*
	20	`(λ (x) (if (> 7 (length x)) empty (singleton (nth 7 x))))`
	40	`(λ (x) (if (is_empty (tail (tail (tail (tail (tail (tail x))))))) empty (cons (head (tail (tail (tail (tail (tail (tail x))))))) empty)))`
		[7, 4] → []<br>[0, 3, 2, 9, 4, 6, 8, 4, 8] → [8]<br>[5, 6, 0, 2, 9, 7, 3, 2, 1, 8] → [3]<br>[9, 9] → []<br>[5, 9, 8, 8, 5, 0, 0, 2] → [0]
0.433	c240	*number of elements equal to the input length*
	15	`(λ (x) (singleton (count (λ (y) (== (length x) y)) x)))`
		[21, 7, 7, 7, 83, 21, 29] → [3]<br>[8, 65, 5, 34, 8, 59, 18, 4] → [2]<br>[60, 24, 51, 8, 72, 9, 98, 2, 65, 1] → [0]<br>[0, 5, 95, 3, 7, 91, 7] → [2]<br>[68, 3, 94, 22, 99, 16, 93, 2, 9] → [1]
0.43	c239	*number of repetitions in the input*
	14	`(λ (x) (singleton (- (length x) (length (unique x)))))`
		[86, 27, 88, 71, 54, 86, 71, 54] → [3]<br>[25, 37, 28, 43, 14, 14, 6, 25, 51] → [2]<br>[13, 84, 48, 60, 84, 60, 21, 21, 96] → [3]<br>[52, 44, 0, 70, 7, 77, 18, 79, 44, 67] → [1]<br>[75, 75, 22, 31, 9, 22, 62, 31, 12, 4] → [3]
0.429	c246	*number of unique elements*
	8	`(λ (x) (singleton (length (unique x))))`
		[76, 5, 19, 22, 19, 3, 9] → [6]<br>[98, 64, 57, 6, 45, 79, 2, 59, 92] → [9]<br>[99, 77, 42, 26, 75, 7, 90, 38] → [8]<br>[] → [0]<br>[37, 87, 33, 24, 18, 2, 17, 71, 47, 66] → [10]
0.428	c138	*remove all occurrences of element 1*
	8	`(λ (x) (cut_vals (first x) x))`
		[7, 32, 7, 32, 87] → [32, 32, 87]<br>[27, 38, 68, 75, 79, 8, 22, 0, 44, 1] → [38, 68, 75, 79, 8, 22, 0, 44, 1]<br>[34, 34, 19, 34, 35, 34] → [19, 35]<br>[17, 17, 17] → []<br>[92, 31, 45, 92, 49, 26, 11, 3, 97] → [31, 45, 49, 26, 11, 3, 97]

μ	ID/$\mathcal{L}$	Description, Program, & Examples
0.427	c018	*replace element 6 with a 3*
	8	`(λ (x) (replace 6 3 x))`
	56	`(λ (x) (fix (cons 6 x) (λ (y z) (if (is_equal (head z) 1) (cons 3 (tail (tail z))) (cons (head (tail z)) (y (cons (- (head z) 1) (tail (tail z)))))))))`
		[7, 7, 7, 7, 7, 7, 7] → [7, 7, 7, 7, 7, 3, 7]<br>[8, 8, 6, 8, 5, 1, 4, 0, 5] → [8, 8, 6, 8, 5, 3, 4, 0, 5]<br>[5, 3, 2, 8, 4, 6] → [5, 3, 2, 8, 4, 3]<br>[9, 9, 3, 0, 0, 9, 0, 0, 9, 3] → [9, 9, 3, 0, 0, 3, 0, 0, 9, 3]<br>[9, 3, 1, 8, 3, 9, 3, 3, 1] → [9, 3, 1, 8, 3, 3, 3, 3, 1]
0.424	c012	*remove all but elements 2 through 4*
	8	`(λ (x) (slice 2 4 x))`
	45	`(λ (x) ((λ (y) (if (is_empty (tail (tail (tail y)))) y (cons (head y) (cons (head (tail y)) (cons (head (tail (tail y))) empty))))) (tail x)))`
		[3, 3, 4, 7, 6] → [3, 4, 7]<br>[7, 8, 2, 0, 4, 2] → [8, 2, 0]<br>[6] → []<br>[2, 9, 4] → [9, 4]<br>[6, 8] → [8]
0.419	c232	*mean value of the input*
	12	`(λ (x) (singleton (/ (sum x) (length x))))`
		[72, 14, 69, 77, 8] → [48]<br>[94, 60, 0, 89, 41] → [56]<br>[18, 10, 16, 4] → [12]<br>[13, 22, 68] → [34]<br>[52, 75, 71] → [66]
0.417	c094	*swap elements 1 and 3 and elements 2 and 4*
	14	`(λ (x) (swap 1 3 (swap 2 4 x)))`
	46	`(λ (x) (cons (head (tail (tail x))) (cons (head (tail (tail (tail x)))) (cons (head x) (cons (head (tail x)) (tail (tail (tail (tail x)))))))))`
		[4, 1, 99, 66, 4] → [99, 66, 4, 1, 4]<br>[6, 35, 46, 67, 7, 7] → [46, 67, 6, 35, 7, 7]<br>[68, 90, 68, 31, 68, 58, 90] → [68, 31, 68, 90, 68, 58, 90]<br>[96, 35, 63, 96, 63, 52, 96, 95, 37, 95] → [63, 96, 96, 35, 63, 52, 96, 95, 37, 95]<br>[73, 73, 5, 5, 73, 5, 5, 5, 5] → [5, 5, 73, 73, 73, 5, 5, 5, 5]
0.414	c086	*swap elements 1 and 4*
	8	`(λ (x) (swap 1 4 x))`
	46	`(λ (x) (cons (head (tail (tail (tail x)))) (cons (head (tail x)) (cons (head (tail (tail x))) (cons (head x) (tail (tail (tail (tail x)))))))))`
		[72, 14, 74, 16, 27] → [16, 14, 74, 72, 27]<br>[56, 0, 49, 15, 49, 80, 18, 80, 18] → [15, 0, 49, 56, 49, 80, 18, 80, 18]<br>[1, 35, 35, 9, 8, 41, 1, 85, 41, 7] → [9, 35, 35, 1, 8, 41, 1, 85, 41, 7]<br>[1, 5, 1, 5, 50, 1, 50] → [5, 5, 1, 1, 50, 1, 50]<br>[69, 3, 39, 51, 8, 51, 3, 3] → [51, 3, 39, 69, 8, 51, 3, 3]
0.413	c193	*input in ascending order, minus an occurrence of both the smallest and largest elements*
	15	`(λ (x) (drop 1 (droplast 1 (sort (λ (y) y) x))))`
		[37, 24, 71, 8, 0, 39, 6, 25, 90] → [6, 8, 24, 25, 37, 39, 71]<br>[6, 5, 7, 34, 78, 29, 23, 26, 1, 28] → [5, 6, 7, 23, 26, 28, 29, 34]<br>[53, 60, 91, 61, 12, 3, 68, 8, 79, 96] → [8, 12, 53, 60, 61, 68, 79, 91]<br>[88, 46, 2, 10, 34, 56, 45, 4, 64] → [4, 10, 34, 45, 46, 56, 64]<br>[57, 77, 16, 17, 27, 44, 0, 42, 1] → [1, 16, 17, 27, 42, 44, 57]

μ	ID/$\mathcal{L}$	Description, Program, & Examples
0.4	c056	*remove element 5*
	6	`(λ (x) (cut_idx 5 x))`
	48	`(λ (x) (cons (head x) (cons (head (tail x)) (cons (head (tail (tail x))) (cons (head (tail (tail (tail x)))) (tail (tail (tail (tail (tail x))))))))))`
		[2, 1, 2, 6, 7, 2] → [2, 1, 2, 6, 2]<br>[5, 6, 9, 6, 6, 5, 9] → [5, 6, 9, 6, 5, 9]<br>[7, 6, 7, 8, 6, 0, 7, 6, 0, 5] → [7, 6, 7, 8, 0, 7, 6, 0, 5]<br>[8, 8, 8, 8, 8, 8, 8, 8] → [8, 8, 8, 8, 8, 8, 8]<br>[1, 1, 1, 1, 1, 1, 1, 1, 1] → [1, 1, 1, 1, 1, 1, 1, 1]
0.396	c028	*remove element 2 if element 1 ¿ element 2, else remove element 3*
	20	`(λ (x) (cut_idx (if (> (first x) (second x)) 2 3) x))`
	42	`(λ (x) (cons (head x) (if (> (head x) (head (tail x))) (tail (tail x)) (cons (head (tail x)) (tail (tail (tail x)))))))`
		[1, 2, 0, 6] → [1, 2, 6]<br>[3, 2, 5, 8, 0, 9, 5] → [3, 5, 8, 0, 9, 5]<br>[5, 7, 1, 9, 0, 6, 2, 8, 4, 7] → [5, 7, 9, 0, 6, 2, 8, 4, 7]<br>[3, 1, 4] → [3, 4]<br>[3, 0, 7, 6, 9, 4] → [3, 7, 6, 9, 4]
0.395	c220	*if input length is even, double each element, else triple it*
	21	`(λ (x) (map (λ (y) (* y (if (is_even (length x)) 2 3))) x))`
		[4, 8, 3, 29, 15] → [12, 24, 9, 87, 45]<br>[5, 39] → [10, 78]<br>[19] → [57]<br>[1, 4, 0, 9, 25, 6, 2, 28, 7] → [3, 12, 0, 27, 75, 18, 6, 84, 21]<br>[43, 23, 11, 5, 8, 30, 41, 2] → [86, 46, 22, 10, 16, 60, 82, 4]
0.394	c241	*number of even elements*
	8	`(λ (x) (singleton (count is_even x)))`
		[49, 87, 13, 67, 4, 5, 8] → [2]<br>[0, 45, 84, 90, 72, 8, 68, 72, 30] → [8]<br>[5, 53, 61, 57, 7, 63, 12, 3] → [1]<br>[22, 22, 10, 6, 28, 26, 26, 16, 22, 26] → [10]<br>[3, 44, 36, 82, 4, 6, 2, 14, 32, 32] → [9]
0.392	c060	*elements 3, 2, 1, the number 4, then elements 5 and 7, in that order*
	22	`(λ (x) (swap 3 1 (replace 4 4 (cut_idx 6 (take 7 x)))))`
	62	`(λ (x) (cons (head (tail (tail x))) (cons (head (tail x)) (cons (head x) (cons 4 (cons (head (tail (tail (tail (tail x))))) (cons (head (tail (tail (tail (tail (tail (tail x))))))) empty)))))))`
		[7, 9, 0, 2, 6, 8, 3, 5, 1] → [0, 9, 7, 4, 6, 3]<br>[1, 7, 8, 2, 5, 6, 0, 4, 3, 9] → [8, 7, 1, 4, 5, 0]<br>[6, 7, 1, 3, 2, 0, 8, 9, 4, 5] → [1, 7, 6, 4, 2, 8]<br>[9, 2, 0, 5, 8, 7, 6, 4, 1, 3] → [0, 2, 9, 4, 8, 6]<br>[9, 2, 5, 1, 3, 4, 7, 0, 6, 8] → [5, 2, 9, 4, 3, 7]
0.391	c169	*second largest element*
	12	`(λ (x) (singleton (max (cut_vals (max x) x))))`
		[26, 68, 87, 84, 58, 10] → [84]<br>[8, 24, 51, 14, 2, 69, 9, 28, 48] → [51]<br>[5, 32, 76, 7, 90, 53, 65, 54] → [76]<br>[36, 99, 8, 9, 16, 67, 94, 0, 4, 40] → [94]<br>[3, 13, 1, 95, 17, 20, 12] → [20]

μ	ID/$\mathcal{L}$	Description, Program, & Examples
0.39	c057	*insert a 4 as element 7*
	8	`(λ (x) (insert 4 7 x))`
	84	`(λ (x) (cons (head x) (cons (head (tail x)) (cons (head (tail (tail x))) (cons (head (tail (tail (tail x)))) (cons (head (tail (tail (tail (tail x))))) (cons (head (tail (tail (tail (tail (tail x)))))) (cons 4 (tail (tail (tail (tail (tail (tail x))))))))))))))`
		[3, 3, 3, 3, 3, 3, 3] → [3, 3, 3, 3, 3, 3, 4, 3]
		[2, 7, 8, 4, 0, 6, 5, 1] → [2, 7, 8, 4, 0, 6, 4, 5, 1]
		[2, 3, 9, 7, 6, 0, 0, 8] → [2, 3, 9, 7, 6, 0, 4, 0, 8]
		[2, 9, 9, 2, 9, 9, 2, 2, 2] → [2, 9, 9, 2, 9, 9, 4, 2, 2, 2]
		[5, 9, 3, 7, 2, 1, 6, 0, 8] → [5, 9, 3, 7, 2, 1, 4, 6, 0, 8]
0.388	c150	*count from element 1 up to each element, in order of appearance*
	31	`(λ (x) (flatten (map (λ (y) (if (> y (first x)) (range (first x) 1 y) (singleton y))) x)))`
		[37, 20, 47, 8, 5] → [37, 20, 37, 38, 39, 40, 41, 42, 43, 44, 45, 46, 47, 8, 5]
		[28, 29, 2] → [28, 28, 29, 2]
		[83, 19, 7, 32, 9, 86] → [83, 19, 7, 32, 9, 83, 84, 85, 86]
		[13, 1, 18] → [13, 1, 13, 14, 15, 16, 17, 18]
		[24, 15, 27, 4] → [24, 15, 24, 25, 26, 27, 4]
0.386	c033	*swap elements 1 and 4*
	8	`(λ (x) (swap 1 4 x))`
	46	`(λ (x) (cons (head (tail (tail (tail x)))) (cons (head (tail x)) (cons (head (tail (tail x))) (cons (head x) (tail (tail (tail (tail x))))))))))`
		[8, 1, 8, 5, 5, 2] → [5, 1, 8, 8, 5, 2]
		[7, 9, 1, 5, 6, 3, 0] → [5, 9, 1, 7, 6, 3, 0]
		[7, 8, 6, 5, 6, 7, 7, 5, 6, 5] → [5, 8, 6, 7, 6, 7, 7, 5, 6, 5]
		[1, 9, 6, 2] → [2, 9, 6, 1]
		[7, 8, 4, 6, 1, 2, 9, 5] → [6, 8, 4, 7, 1, 2, 9, 5]
0.386	c218	*keep only elements 2 and following equal to element 1*
	17	`(λ (x) (filter (λ (y) (== y (first x))) (drop 1 x)))`
		[93, 93, 52, 93, 99, 99, 71, 52] → [93, 93]
		[90, 61, 9, 9, 37, 90, 30, 3, 3] → [90]
		[55, 2, 55, 6, 6, 19, 53, 8, 55, 55] → [55, 55, 55]
		[15, 25, 73, 6, 7, 20, 67, 13, 23] → []
		[45, 27, 1, 45, 1, 27, 45, 45, 45, 1] → [45, 45, 45, 45]
0.385	c014	*remove all but elements 3 through 7*
	8	`(λ (x) (slice 3 7 x))`
	65	`(λ (x) ((λ (y) (fix (cons 5 y) (λ (z u) (if (is_equal 0 (head u)) empty (if (is_empty (tail u)) empty (cons (head (tail u)) (z (cons (- (head u) 1) (tail (tail u)))))))))) (tail (tail x))))`
		[6, 3, 0, 4] → [0, 4]
		[9, 9, 1] → [1]
		[0, 7, 9, 6, 1, 8, 2, 3] → [9, 6, 1, 8, 2]
		[3, 0, 5, 7, 7, 5, 3, 0, 5] → [5, 7, 7, 5, 3]
		[9] → []
0.376	c136	*remove the first occurrence of the largest element*
	8	`(λ (x) (cut_val (max x) x))`
		[95, 95, 73, 95, 23] → [95, 73, 95, 23]
		[35, 22, 46, 94, 94, 52] → [35, 22, 46, 94, 52]
		[2, 90, 8, 90, 72, 14, 13, 1, 80] → [2, 8, 90, 72, 14, 13, 1, 80]
		[92, 92] → [92]
		[61, 79, 89, 71, 74, 20, 30, 62, 67, 3] → [61, 79, 71, 74, 20, 30, 62, 67, 3]

μ	ID/$\mathcal{L}$	Description, Program, & Examples
0.352	c083	*remove all but element 7*
	8	`(λ (x) (singleton (nth 7 x)))`
	20	`(λ (x) (cons (head (tail (tail (tail (tail (tail (tail x))))))) empty))`
		[47, 27, 63, 1, 28, 26, 82, 1] → [82]
		[67, 3, 20, 9, 50, 72, 29, 9, 35, 82] → [29]
		[3, 4, 23, 57, 46, 60, 70, 7] → [70]
		[6, 4, 54, 54, 5, 5, 4, 68] → [4]
		[0, 6, 89, 5, 13, 89, 21, 21, 3, 30] → [21]
0.351	c019	*replace element 6 with a 3 if there is an element 6*
	8	`(λ (x) (replace 6 3 x))`
	66	`(λ (x) (fix (cons 6 x) (λ (y z) (if (is_empty (tail z)) empty (if (is_equal (head z) 1) (cons 3 (tail (tail z))) (cons (head (tail z)) (y (cons (- (head z) 1) (tail (tail z))))))))))`
		[4, 5, 5, 0, 4, 2] → [4, 5, 5, 0, 4, 3]
		[6, 6] → [6, 6]
		[3, 7, 2, 0, 4, 9, 8] → [3, 7, 2, 0, 4, 3, 8]
		[1, 1, 7, 4, 0, 8, 9, 0] → [1, 1, 7, 4, 0, 3, 9, 0]
		[4, 3, 5, 8, 3, 2, 7, 9, 1] → [4, 3, 5, 8, 3, 3, 7, 9, 1]
0.347	c203	*the first M positive multiples of the smallest element, where M is the input's length*
	14	`(λ (x) (mapi (λ (y z) (* (min x) y)) x))`
		[7, 3, 5, 2, 1] → [1, 2, 3, 4, 5]
		[37, 82] → [37, 74]
		[23, 92, 84] → [23, 46, 69]
		[67, 64, 29, 99, 8, 62, 22, 81, 44] → [8, 16, 24, 32, 40, 48, 56, 64, 72]
		[36, 78, 19, 89] → [19, 38, 57, 76]
0.331	c115	*the unique elements, prepended and appended by their sum*
	20	`(λ (x) (cons (sum (unique x)) (append (unique x) (sum (unique x)))))`
		[7, 17, 45, 17, 12] → [81, 7, 17, 45, 12, 81]
		[4, 4, 31, 38, 38, 31, 38, 38] → [73, 4, 31, 38, 73]
		[24, 3, 24, 1, 3, 2, 42, 2, 1, 42] → [72, 24, 3, 1, 2, 42, 72]
		[27, 14, 14, 14, 14, 27, 27, 27, 27] → [41, 27, 14, 41]
		[] → [0, 0]
0.329	c207	*element - wise sum of the input and the reversed input*
	12	`(λ (x) (map sum (zip x (reverse x))))`
		[7, 6, 9, 79, 46] → [53, 85, 18, 85, 53]
		[14, 8, 22] → [36, 16, 36]
		[1, 31, 84, 4, 68, 89] → [90, 99, 88, 88, 99, 90]
		[19, 1, 97, 62] → [81, 98, 98, 81]
		[5, 13, 51] → [56, 26, 56]
0.325	c088	*swap elements 1 and 4 if element 2 = element 3, else swap elements 2 and 3*
	28	`(λ (x) (if (== (second x) (third x)) (swap 1 4 x) (swap 2 3 x)))`
	96	`(λ (x) (if (is_equal (head (tail x)) (head (tail (tail x)))) (cons (head (tail (tail (tail x)))) (cons (head (tail x)) (cons (head (tail (tail x))) (cons (head x) (tail (tail (tail (tail x)))))))) (cons (head x) (cons (head (tail (tail x))) (cons (head (tail x)) (tail (tail (tail x))))))))`
		[19, 1, 99, 19, 20, 99, 20] → [19, 99, 1, 19, 20, 99, 20]
		[95, 31, 31, 4, 39, 5, 32, 0] → [4, 31, 31, 95, 39, 5, 32, 0]
		[15, 53, 53, 95] → [95, 53, 53, 15]
		[84, 86, 3, 84, 3, 89] → [84, 3, 86, 84, 3, 89]
		[17, 41, 41, 85, 25, 2, 17, 25, 17, 10] → [85, 41, 41, 17, 25, 2, 17, 25, 17, 10]

μ	ID/$\mathcal{L}$	Description, Program, & Examples
0.322	c040	*append 3 if the list contains a 3, else append 9 if the list contains a 9*
	30	`(λ (x) (if (is_in x 3) (append x 3) (if (is_in x 9) (append x 9) x)))`
	86	`(λ (x) ((λ (y) (fix x (λ (z u) (if (is_empty u) (if (y 3) (cons 3 empty) (if (y 9) (cons 9 empty) empty)) (cons (head u) (z (tail u))))))) (λ (v) (fix x (λ (w a) (if (is_equal a empty) false (if (is_equal (head a) v) true (w (tail a)))))))))`
		[4, 8, 3, 7, 8] → [4, 8, 3, 7, 8, 3]<br>[5, 8, 2, 9, 0, 0] → [5, 8, 2, 9, 0, 0, 9]<br>[3, 8, 5] → [3, 8, 5, 3]<br>[9, 1, 1, 5, 1, 6, 5, 6] → [9, 1, 1, 5, 1, 6, 5, 6, 9]<br>[7, 0] → [7, 0]
0.32	c085	*remove all but element N + 1, N = element 1*
	14	`(λ (x) (singleton (nth (first x) (drop 1 x))))`
	44	`(λ (x) (fix x (λ (y z) (if (is_equal 1 (head z)) (cons (head (tail z)) empty) (y (cons (- (head z) 1) (tail (tail z))))))))`
		[4, 39, 4, 48, 46, 48] → [46]<br>[2, 67, 52, 72, 93, 9, 67, 5, 68] → [52]<br>[5, 4, 3, 80, 36, 66, 75] → [66]<br>[4, 28, 5, 26, 29, 6, 94, 3] → [29]<br>[1, 4, 4] → [4]
0.316	c111	*repeat the largest element N times, where N is the smallest element*
	10	`(λ (x) (repeat (max x) (min x)))`
		[38, 76, 18, 31, 5] → [76, 76, 76, 76, 76]<br>[90, 28, 72, 2, 5, 94, 85, 88, 68] → [94, 94]<br>[52, 75, 83, 30, 3, 93] → [93, 93, 93]<br>[1, 82, 56, 49, 1, 60, 60, 90] → [90]<br>[0, 24, 65] → []
0.316	c073	*add the index to every element*
	6	`(λ (x) (mapi + x))`
	52	`(λ (x) (fix (cons 1 x) (λ (y z) (if (is_empty (tail z)) empty (cons (+ (head z) (head (tail z))) (y (cons (+ 1 (head z)) (tail (tail z)))))))))`
		[4, 4, 4, 4] → [5, 6, 7, 8]<br>[1] → [2]<br>[5, 5, 5, 2, 2, 2] → [6, 7, 8, 6, 7, 8]<br>[1, 3, 3, 5, 3, 0, 0, 1, 0] → [2, 5, 6, 9, 8, 6, 7, 9, 9]<br>[3, 3] → [4, 5]
0.315	c027	*remove element 2 if element 1 = element 2, else remove element 3*
	20	`(λ (x) (cut_idx (if (== (first x) (second x)) 2 3) x))`
	40	`(λ (x) (if (is_equal (head x) (head (tail x))) (tail x) (cons (head x) (cons (head (tail x)) (tail (tail (tail x)))))))`
		[0, 0, 9, 0] → [0, 9, 0]<br>[8, 8, 4, 1, 8, 4, 1, 4] → [8, 4, 1, 8, 4, 1, 4]<br>[6, 4, 6, 2, 3, 3] → [6, 4, 2, 3, 3]<br>[1, 2, 5, 1, 3, 2, 5] → [1, 2, 1, 3, 2, 5]<br>[9, 9, 7, 7, 7, 6, 6, 7, 2] → [9, 7, 7, 7, 6, 6, 7, 2]
0.312	c234	*repetitions in order of first appearance*
	18	`(λ (x) (flatten (map (λ (y) (drop 1 y)) (group (λ (z) z) x))))`
		[66, 0, 66, 58, 25, 0, 25, 92] → [66, 0, 25]<br>[48, 1, 6, 94, 96, 65, 82, 45, 90, 5] → []<br>[7, 26, 84, 6, 62, 77, 2, 84, 10, 80] → [84]<br>[3, 22, 5, 24, 1, 22, 21, 19, 5, 2] → [22, 5]<br>[4, 16, 85, 16, 8, 16, 4, 85, 85] → [4, 16, 16, 85, 85]

μ	ID/$\mathcal{L}$	Description, Program, & Examples
0.307	c165	*reverse the input and keep only even elements*
	8	`(λ (x) (filter is_even (reverse x)))`
		[68, 9, 86, 13, 57, 14, 72, 25, 69] → [72, 14, 86, 68]<br>[77, 5, 56, 4, 34, 22, 65, 94, 20, 3] → [20, 94, 22, 34, 4, 56]<br>[42, 7, 59, 1, 92, 48, 24, 63, 5, 8] → [8, 24, 48, 92, 42]<br>[53, 5, 0, 7, 78, 43, 45, 39, 2] → [2, 78, 0]<br>[54, 0, 97, 79, 99, 50, 6, 93, 3, 84] → [84, 6, 50, 0, 54]
0.306	c191	*repeat each element N times, where N is its tens digit, in order of appearance*
	17	`(λ (x) (flatten (map (λ (y) (repeat y (/ y 10))) x)))`
		[2, 0, 7, 30, 26] → [30, 30, 30, 26, 26]<br>[37] → [37, 37, 37]<br>[1, 12, 7, 9, 8, 23, 0, 6, 15] → [12, 23, 23, 15]<br>[55, 5, 7, 20, 1, 27] → [55, 55, 55, 55, 55, 20, 20, 27, 27]<br>[3, 4, 2] → []
0.303	c026	*remove element 3*
	6	`(λ (x) (cut_idx 3 x))`
	22	`(λ (x) (cons (head x) (cons (head (tail x)) (tail (tail (tail x))))))`
		[4, 2, 2, 5] → [4, 2, 5]<br>[0, 7, 7, 4, 6] → [0, 7, 4, 6]<br>[3, 9, 8, 1, 6, 2, 0, 4, 5, 7] → [3, 9, 1, 6, 2, 0, 4, 5, 7]<br>[1, 1, 3, 2, 2, 3, 3] → [1, 1, 2, 2, 3, 3]<br>[8, 3, 9] → [8, 3]
0.3	c059	*swap elements 4 and 8*
	8	`(λ (x) (swap 4 8 x))`
	122	`(λ (x) (cons (head x) (cons (head (tail x)) (cons (head (tail (tail x))) (cons (head (tail (tail (tail (tail (tail (tail (tail x)))))))) (cons (head (tail (tail (tail (tail x))))) (cons (head (tail (tail (tail (tail (tail x)))))) (cons (head (tail (tail (tail (tail (tail (tail x))))))) (cons (head (tail (tail (tail x)))) (tail (tail (tail (tail (tail (tail (tail (tail x))))))))))))))))))`
		[0, 5, 3, 8, 1, 9, 4, 6, 2] → [0, 5, 3, 6, 1, 9, 4, 8, 2]<br>[6, 1, 8, 5, 2, 3, 7, 9, 0, 4] → [6, 1, 8, 9, 2, 3, 7, 5, 0, 4]<br>[1, 0, 7, 8, 6, 4, 2, 6, 1, 9] → [1, 0, 7, 6, 6, 4, 2, 8, 1, 9]<br>[9, 5, 5, 9, 3, 7, 6, 3, 9, 3] → [9, 5, 5, 3, 3, 7, 6, 9, 9, 3]<br>[7, 3, 4, 0, 1, 6, 8, 1, 5] → [7, 3, 4, 1, 1, 6, 8, 0, 5]
0.298	c074	*remove every element whose value ¡ 8*
	11	`(λ (x) (filter (λ (y) (> y 7)) x))`
	42	`(λ (x) (fix x (λ (y z) (if (is_empty z) empty (if (> (head z) 7) (cons (head z) (y (tail z))) (y (tail z)))))))`
		[8, 2, 7, 6, 8, 6] → [8, 8]<br>[9, 2, 0, 5, 7, 5, 2, 3, 4, 7] → [9]<br>[8, 8, 8, 2, 7, 7, 2] → [8, 8, 8]<br>[0] → []<br>[8, 9, 9, 1, 1, 9, 8, 8, 9, 1] → [8, 9, 9, 9, 8, 8, 9]
0.295	c173	*replace element N with the largest element in elements 1 through N*
	14	`(λ (x) (mapi (λ (y z) (max (take y x))) x))`
		[2, 6, 74, 86, 1, 89] → [2, 6, 74, 86, 86, 89]<br>[8, 9, 19, 2, 67, 83, 53, 4, 56, 95] → [8, 9, 19, 19, 67, 83, 83, 83, 83, 95]<br>[3, 5, 9, 50, 7, 62, 78, 0] → [3, 5, 9, 50, 50, 62, 78, 78]<br>[9, 11, 21, 25, 55, 48, 7, 1, 70] → [9, 11, 21, 25, 55, 55, 55, 55, 70]<br>[47, 66, 81, 0, 1, 99, 4] → [47, 66, 81, 81, 81, 99, 99]

μ	ID/$\mathcal{L}$	Description, Program, & Examples
0.294	c175	*keep only elements larger than any preceding element*
	34	`(λ (x) (fold (λ (y z) (if (> z (last y)) (append y z) y)) (take 1 x) (drop 1 x)))`
		[45, 58, 87, 48, 31, 34] → [45, 58, 87]<br>[7, 8, 39, 95, 11, 1, 72] → [7, 8, 39, 95]<br>[2, 44, 50, 62, 65, 9, 3, 8, 88, 91] → [2, 44, 50, 62, 65, 88, 91]<br>[2, 25, 39, 51, 16, 5, 66, 7] → [2, 25, 39, 51, 66]<br>[4, 31, 40, 6, 63, 1, 3, 66, 88] → [4, 31, 40, 63, 66, 88]
0.294	c204	*keep only elements followed by 0*
	29	`(λ (x) (map first (filter (λ (y) (== (second y) 0)) (zip (droplast 1 x) (drop 1 x)))))`
		[9, 34, 0, 0, 96] → [34, 0]<br>[4, 0, 29, 3, 0, 5, 6, 51] → [4, 3]<br>[29, 28, 0, 2, 0, 64] → [28, 2]<br>[13, 53, 88, 0, 6, 65, 21, 0, 9] → [88, 21]<br>[28, 4, 97, 34, 14, 0, 0, 1, 0, 88] → [14, 0, 1]
0.293	c031	*remove whichever are equal: the two elements or the last two*
	20	`(λ (x) (if (== (first x) (second x)) drop droplast 2 x))`
	50	`(λ (x) (if (is_equal (head x) (head (tail x))) (tail (tail x)) (fix x (λ (y z) (if (is_empty (tail (tail z))) empty (cons (head z) (y (tail z))))))))`
		[6, 6, 6, 8] → [6, 8]<br>[0, 0, 5, 5, 0, 5, 5, 0] → [5, 5, 0, 5, 5, 0]<br>[1, 4, 4] → [1]<br>[4, 4, 8, 3, 4, 9, 9, 9, 3, 6] → [8, 3, 4, 9, 9, 9, 3, 6]<br>[4, 0, 2, 4, 2, 2, 7, 9, 9] → [4, 0, 2, 4, 2, 2, 7]
0.292	c236	*even elements divided by 2*
	15	`(λ (x) (map (λ (y) (/ y 2)) (filter is_even x)))`
		[18, 37, 3, 50, 13, 95, 9] → [9, 25]<br>[25, 24, 7, 5, 38, 52, 74, 94] → [12, 19, 26, 37, 47]<br>[92, 84, 9, 23, 7, 87, 73, 28, 90] → [46, 42, 14, 45]<br>[64, 80, 8, 20, 2, 7, 6, 0, 44, 12] → [32, 40, 4, 10, 1, 3, 0, 22, 6]<br>[86, 53, 99, 5, 30, 65, 72, 93, 43] → [43, 15, 36]
0.286	c054	*replace elements 1 and 2 with element 3*
	16	`(λ (x) (concat (repeat (third x) 3) (drop 3 x)))`
	26	`(λ (x) (cons (head (tail (tail x))) (cons (head (tail (tail x))) (tail (tail x)))))`
		[5, 5, 9, 5] → [9, 9, 9, 5]<br>[7, 5, 2, 5, 7, 7, 2] → [2, 2, 2, 5, 7, 7, 2]<br>[4, 1, 1, 1, 1, 4, 4, 1, 3, 3] → [1, 1, 1, 1, 1, 4, 4, 1, 3, 3]<br>[9, 5, 9, 2, 3, 8, 2, 3, 8] → [9, 9, 9, 2, 3, 8, 2, 3, 8]<br>[9, 3, 6, 7, 0] → [6, 6, 6, 7, 0]
0.267	c227	*interleave input and reversed input; keep only unique elements in order of appearance*
	12	`(λ (x) (unique (flatten (zip x (reverse x)))))`
		[9, 66, 10, 0, 43, 66, 9] → [9, 66, 10, 43, 0]<br>[85, 39, 0, 33, 26, 27, 83, 18] → [85, 18, 39, 83, 0, 27, 33, 26]<br>[40, 75, 49, 75, 40, 49, 49, 68, 49] → [40, 49, 75, 68]<br>[55, 17, 20, 89, 22, 5, 20, 6, 65, 1] → [55, 1, 17, 65, 20, 6, 89, 22, 5]<br>[65, 99, 86, 86, 28, 42, 7, 42, 53, 86] → [65, 86, 99, 53, 42, 7, 28]

μ	ID/$\mathcal{L}$	Description, Program, & Examples
0.265	c024	*insert as element 2: 8 if element 1 ¿ 5 else 5*
	20	`(λ (x) (insert (if (> 5 (first x)) 8 5) 2 x))`
	26	`(λ (x) (cons (head x) (cons (if (> 5 (head x)) 8 5) (tail x))))`
		[8, 7, 4, 1] → [8, 5, 7, 4, 1]<br>[0, 8, 3] → [0, 8, 8, 3]<br>[9, 9, 4, 9, 6, 1] → [9, 5, 9, 4, 9, 6, 1]<br>[7, 6, 0, 7, 6] → [7, 5, 6, 0, 7, 6]<br>[5, 2] → [5, 5, 2]
0.264	c200	*tens digits of the elements in ascending order*
	16	`(λ (x) (sort (λ (y) y) (map (λ (z) (/ z 10)) x)))`
		[49, 0, 24, 33, 92] → [0, 2, 3, 4, 9]<br>[5, 54, 41, 72] → [0, 4, 5, 7]<br>[68, 5, 91, 59, 36, 18, 71] → [0, 1, 3, 5, 6, 7, 9]<br>[14, 89, 46, 34, 79, 0] → [0, 1, 3, 4, 7, 8]<br>[87, 90, 16] → [1, 8, 9]
0.256	c010	*elements 2 through N + 1, N = element 1*
	12	`(λ (x) (take (first x) (drop 1 x)))`
	44	`(λ (x) (fix x (λ (y z) (if (is_equal 0 (head z)) empty (cons (head (tail z)) (y (cons (- (head z) 1) (tail (tail z)))))))))`
		[2, 3, 2, 7, 6] → [3, 2]<br>[3, 9, 8, 6, 5, 1] → [9, 8, 6]<br>[1, 2, 4, 5, 0, 8, 9, 7, 8] → [2]<br>[5, 5, 5, 1, 1, 5] → [5, 5, 1, 1, 5]<br>[0, 2] → []
0.256	c123	*remove all but element N, N = last element*
	10	`(λ (x) (singleton (nth (last x) x)))`
		[28, 48, 57, 36, 4] → [36]<br>[90, 54, 16, 3] → [16]<br>[22, 9, 14, 87, 71, 3] → [14]<br>[72, 1] → [72]<br>[69, 63, 50, 8, 86, 17, 0, 80, 19, 7] → [0]
0.256	c023	*insert as element 2: 8 if the list length ¿ 5 else 5*
	20	`(λ (x) (insert (if (> 5 (length x)) 8 5) 2 x))`
	46	`(λ (x) (cons (head x) (cons (if (> 5 (fix x (λ (y z) (if (is_empty z) 0 (+ 1 (y (tail z))))))) 8 5) (tail x))))`
		[2, 0, 5, 4] → [2, 8, 0, 5, 4]<br>[0, 2, 7, 9, 5, 8, 6, 3, 0, 9] → [0, 5, 2, 7, 9, 5, 8, 6, 3, 0, 9]<br>[9, 7, 6, 1, 2] → [9, 5, 7, 6, 1, 2]<br>[8, 8] → [8, 8, 8]<br>[7, 0, 2] → [7, 8, 0, 2]
0.256	c194	*reverse the input; prepend and append the input's length*
	16	`(λ (x) (cons (length x) (append (reverse x) (length x))))`
		[76, 62, 80, 54, 23] → [5, 23, 54, 80, 62, 76, 5]<br>[81, 43] → [2, 43, 81, 2]<br>[1, 63, 21, 16] → [4, 16, 21, 63, 1, 4]<br>[92, 51, 35, 20, 9, 0, 18] → [7, 18, 0, 9, 20, 35, 51, 92, 7]<br>[39, 90, 8] → [3, 8, 90, 39, 3]

μ	ID/$\mathcal{L}$	**Description, Program, & Examples**
0.25	c245	*number of times element 1 appears in elements 2 and following*
	19	`(λ (x) (singleton (count (λ (y) (== (first x) y)) (drop 1 x))))`
		[5, 5, 5, 5, 5, 41, 5] → [5]<br>[67, 23, 84, 30, 18, 80, 1, 69, 28] → [0]<br>[1, 40, 1, 3, 51, 9, 91, 1, 2, 1] → [3]<br>[59, 87, 59, 91, 53, 0, 2, 62, 76, 61] → [1]<br>[18, 49, 72, 7, 71, 8, 27, 97] → [0]
0.242	c032	*remove two elements: the first two if element 1 ¿ last element, else the last two*
	20	`(λ (x) (if (> (first x) (last x)) drop droplast 2 x))`
	68	`(λ (x) (if (> (head x) (fix x (λ (y z) (if (is_empty (tail z)) (head z) (y (tail z)))))) (tail (tail x)) (fix x (λ (u v) (if (is_empty (tail (tail v))) empty (cons (head v) (u (tail v))))))))`
		[0, 1, 7, 9, 3] → [0, 1, 7]<br>[7, 6, 4, 4, 1, 8, 3] → [4, 4, 1, 8, 3]<br>[6, 3, 2, 9, 9, 2, 6, 2] → [2, 9, 9, 2, 6, 2]<br>[0, 9, 4, 6, 8, 2, 5, 7, 1] → [0, 9, 4, 6, 8, 2, 5]<br>[3, 7, 0, 5, 1, 4, 8, 6, 2, 9] → [3, 7, 0, 5, 1, 4, 8, 6]
0.242	c058	*remove the first 7 elements*
	6	`(λ (x) (drop 7 x))`
	16	`(λ (x) (tail (tail (tail (tail (tail (tail (tail x))))))))`
		[7, 2, 9, 5, 3, 6, 4, 5] → [5]<br>[9, 8, 9, 8, 9, 8, 5, 0, 2, 5] → [0, 2, 5]<br>[3, 0, 1, 4, 8, 2, 7] → []<br>[2, 8, 6, 3, 9, 5, 7, 6, 4] → [6, 4]<br>[9, 2, 4, 8, 0, 5, 3, 1, 7] → [1, 7]
0.242	c174	*keep the first N elements, where N is the number of unique elements*
	10	`(λ (x) (take (length (unique x)) x))`
		[49, 32, 85, 49, 32, 2] → [49, 32, 85, 49]<br>[29, 0, 77, 35, 50, 7, 53, 35, 8, 82] → [29, 0, 77, 35, 50, 7, 53, 35, 8]<br>[66, 71, 9, 72, 11, 86, 91, 9] → [66, 71, 9, 72, 11, 86, 91]<br>[67, 1, 24, 37, 5, 18, 67] → [67, 1, 24, 37, 5, 18]<br>[9, 52, 96, 27, 83, 4, 42, 98, 4] → [9, 52, 96, 27, 83, 4, 42, 98]
0.238	c229	*keep only first N elements of the reversed input, N = element 1*
	10	`(λ (x) (take (first x) (reverse x)))`
		[7, 58, 5, 9, 21, 22, 51] → [51, 22, 21, 9, 5, 58, 7]<br>[4, 89, 16, 33, 53, 3, 6, 1, 76] → [76, 1, 6, 3]<br>[5, 18, 99, 7, 7, 99, 81, 11] → [11, 81, 99, 7, 7]<br>[1, 3, 49, 5, 2, 15, 77, 68, 27, 13] → [13]<br>[9, 2, 72, 56, 1, 0, 26, 69, 95, 86] → [86, 95, 69, 26, 0, 1, 56, 72, 2]
0.237	c162	*replace each element, M, with 3 * M + 7*
	15	`(λ (x) (map (λ (y) (+ 7 (* 3 y))) x))`
		[8, 0, 17, 5, 5, 0] → [31, 7, 58, 22, 22, 7]<br>[3, 9, 1, 7, 2, 4, 8, 0, 15, 5] → [16, 34, 10, 28, 13, 19, 31, 7, 52, 22]<br>[18, 1, 5, 11, 2, 1, 18] → [61, 10, 22, 40, 13, 10, 61]<br>[8, 7, 3, 9, 5, 1, 5, 1, 4] → [31, 28, 16, 34, 22, 10, 22, 10, 19]<br>[19, 3, 4, 1, 6, 2, 0, 9] → [64, 16, 19, 10, 25, 13, 7, 34]

μ	ID/$\mathcal{L}$	Description, Program, & Examples
0.231	c039	*append 3 if the list length is 3, else append 9 if the list length is 9*
	34	`(λ (x) (if (== (length x) 3) (append x 3) (if (== (length x) 9) (append x 9) x)))`
	79	`(λ (x) ((λ (y) (fix x (λ (z u) (if (is_empty u) (if (is_equal y 3) (cons 3 empty) (if (is_equal y 9) (cons 9 empty) empty)) (cons (head u) (z (tail u))))))) (fix x (λ (v w) (if (is_empty w) 0 (+ 1 (v (tail w))))))))`
		[9, 3, 6] → [9, 3, 6, 3]
		[2, 1, 0, 1, 7, 8, 1, 8, 7] → [2, 1, 0, 1, 7, 8, 1, 8, 7, 9]
		[9, 1, 4] → [9, 1, 4, 3]
		[0, 5, 6, 5, 5] → [0, 5, 6, 5, 5]
		[4, 5, 8, 4, 0, 2, 8, 7, 2] → [4, 5, 8, 4, 0, 2, 8, 7, 2, 9]
0.23	c181	*reverse the order of elements with even indices*
	26	`(λ (x) (flatten (zip (filteri (λ (y z) (is_odd y)) x) (reverse (filteri (λ (u v) (is_even u)) x)))))`
		[24, 99, 36, 61, 55, 6] → [24, 6, 36, 61, 55, 99]
		[1, 53, 21, 2, 57, 48, 74, 7] → [1, 7, 21, 48, 57, 2, 74, 53]
		[16, 97, 40, 26, 35, 65, 63, 59] → [16, 59, 40, 65, 35, 26, 63, 97]
		[4, 19, 51, 96, 33, 3] → [4, 3, 51, 96, 33, 19]
		[39, 50, 8, 82, 68, 52, 1, 89, 14, 5] → [39, 5, 8, 89, 68, 52, 1, 82, 14, 50]
0.23	c163	*replace each element, M, with 2 * M - 10*
	15	`(λ (x) (map (λ (y) (- (* y 2) 10)) x))`
		[7, 9, 8, 24, 23] → [4, 8, 6, 38, 36]
		[6, 8, 47, 6] → [2, 6, 84, 2]
		[7, 11, 7, 5] → [4, 12, 4, 0]
		[9, 5, 22, 18] → [8, 0, 34, 26]
		[33, 19, 29, 8] → [56, 28, 48, 6]
0.22	c154	*triple each even element*
	19	`(λ (x) (map (λ (y) (if (is_even y) (* 3 y) y)) x))`
		[5, 93, 14, 73, 4, 8] → [5, 93, 42, 73, 12, 24]
		[77, 0, 6, 8, 35, 7, 22, 21] → [77, 0, 18, 24, 35, 7, 66, 21]
		[81, 23, 89, 6, 9, 2, 1, 5, 55] → [81, 23, 89, 18, 9, 6, 1, 5, 55]
		[71, 75, 8, 1, 99, 6, 4] → [71, 75, 24, 1, 99, 18, 12]
		[6, 3, 16, 53, 20, 47, 69, 5, 33, 0] → [18, 3, 48, 53, 60, 47, 69, 5, 33, 0]
0.215	c166	*unique elements in ascending order by the sum of their digits*
	21	`(λ (x) (sort (λ (y) (+ (% y 10) (/ y 10))) (unique x)))`
		[43, 20, 1, 20, 17, 55] → [1, 20, 43, 17, 55]
		[92, 24, 11, 25, 21, 53, 25, 21] → [11, 21, 24, 25, 53, 92]
		[86, 7, 63, 81, 9, 97, 41, 86, 3] → [3, 41, 7, 63, 81, 9, 86, 97]
		[70, 70, 50, 70, 8, 50, 8, 50, 8, 8] → [50, 70, 8]
		[58, 58, 58, 82, 58, 82, 58] → [82, 58]
0.209	c084	*remove all but element 7*
	20	`(λ (x) (if (> 7 (length x)) empty (singleton (nth 7 x))))`
	40	`(λ (x) (if (is_empty (tail (tail (tail (tail (tail (tail x))))))) empty (cons (head (tail (tail (tail (tail (tail (tail x))))))) empty)))`
		[2, 42] → []
		[90, 8, 5, 34, 79, 65, 8, 48, 79, 9] → [8]
		[94, 0, 58, 62, 7, 3, 1, 55, 88] → [1]
		[5, 5] → []
		[85, 18, 85, 91, 91, 18, 91] → [91]

μ	ID/$\mathcal{L}$	Description, Program, & Examples
0.2	c249	*use 0s to delimit sublists; give element 1 of each sublist*
	46	`(λ (x) (map first (reverse (fold (λ (y z) (if (== z 0) (cons empty y) (cons (append (first y) z) (drop 1 y)))) (singleton empty) x))))`
		[64, 0, 50, 1, 50, 1, 0, 1, 64] → [64, 50, 1]<br>[8, 0, 4, 68, 68, 78, 0, 68, 25, 68] → [8, 4, 68]<br>[94, 9, 3, 5, 5, 0, 5, 0, 95] → [94, 5, 95]<br>[2, 3, 67, 0, 44, 0, 6, 91, 76] → [2, 44, 6]<br>[42, 37, 80, 0, 47, 13, 80, 0, 80, 42] → [42, 47, 80]
0.199	c118	*left - rotate by N elements, N = last element*
	18	`(λ (x) (concat (drop (last x) x) (take (last x) x)))`
		[24, 8, 57, 44, 4] → [4, 24, 8, 57, 44]<br>[97, 28, 30, 5, 48, 7, 2, 76, 9, 1] → [28, 30, 5, 48, 7, 2, 76, 9, 1, 97]<br>[18, 96, 25, 71, 99, 1] → [96, 25, 71, 99, 1, 18]<br>[39, 85, 2] → [2, 39, 85]<br>[5, 6, 22, 44, 90, 11, 3] → [44, 90, 11, 3, 5, 6, 22]
0.199	c152	*replace each element with the product of its digits*
	19	`(λ (x) (map (λ (y) (* (/ y 10) (% y 10))) x))`
		[37, 98, 4, 19, 82] → [21, 72, 0, 9, 16]<br>[87, 5, 77, 7, 54, 67, 8] → [56, 0, 49, 0, 20, 42, 0]<br>[59, 47, 46] → [45, 28, 24]<br>[65, 25] → [30, 10]<br>[88] → [64]
0.197	c237	*cumulative sum of unique elements*
	32	`(λ (x) (fold (λ (y z) (append y (+ (last y) z))) (take 1 (unique x)) (drop 1 (unique x))))`
		[14, 1, 3, 2, 14, 3] → [14, 15, 18, 20]<br>[7, 15, 2, 1, 0, 20, 20, 0, 4] → [7, 22, 24, 25, 25, 45, 49]<br>[1, 4, 1, 7, 0, 3, 2, 0] → [1, 5, 12, 12, 15, 17]<br>[0, 7, 4, 3, 8, 16, 16, 6, 2, 0] → [0, 7, 11, 14, 22, 38, 44, 46]<br>[6, 7, 8, 5, 7, 9, 7] → [6, 13, 21, 26, 35]
0.196	c243	*number of odd elements*
	8	`(λ (x) (singleton (count is_odd x)))`
		[6, 92, 34, 20, 69, 34, 0] → [1]<br>[19, 5, 4, 33, 17, 3, 19, 45, 93, 7] → [9]<br>[24, 21, 15, 94, 32, 47, 2, 3] → [4]<br>[2, 25, 18, 8, 46, 29, 38, 99] → [3]<br>[75, 8, 55, 5, 42, 62, 67, 89, 43] → [6]
0.194	c188	*replace each element with 1 if it is divisible by 3, else 0*
	21	`(λ (x) (map (λ (y) (if (== (% y 3) 0) 1 0)) x))`
		[84, 9, 10, 11, 4] → [1, 1, 0, 0, 0]<br>[79, 3, 48, 13, 53, 41, 22, 2, 5] → [0, 1, 1, 0, 0, 0, 0, 0, 0]<br>[95, 28, 86, 2, 21, 6, 40, 55] → [0, 0, 0, 0, 1, 1, 0, 0]<br>[90, 60] → [1, 1]<br>[38, 51, 9] → [0, 1, 1]
0.192	c139	*remove all occurrences of the smallest and largest elements*
	14	`(λ (x) (cut_vals (max x) (cut_vals (min x) x)))`
		[28, 97, 22, 97, 22] → [28]<br>[0, 85, 96, 30] → [85, 30]<br>[20, 45, 76, 66, 92, 52, 7, 8] → [20, 45, 76, 66, 52, 8]<br>[0, 4, 38, 1, 88, 88, 62, 2, 81, 87] → [4, 38, 1, 62, 2, 81, 87]<br>[14, 13, 91, 43, 13, 17] → [14, 43, 17]

μ	ID/$\mathcal{L}$	**Description, Program, & Examples**
0.189	c036	*swap elements 2 and 3 if element 2 ¿ element 3, else swap elements 1 and 4*
	28	`(λ (x) (if (> (second x) (third x)) (swap 2 3 x) (swap 1 4 x)))`
	96	`(λ (x) (if (> (head (tail x)) (head (tail (tail x)))) (cons (head x) (cons (head (tail (tail x))) (cons (head (tail x)) (tail (tail (tail x)))))) (cons (head (tail (tail (tail x)))) (cons (head (tail x)) (cons (head (tail (tail x))) (cons (head x) (tail (tail (tail (tail x))))))))))`
		[1, 4, 0, 4] → [1, 0, 4, 4]
		[6, 0, 8, 2, 5, 2, 1, 7, 3, 4] → [2, 0, 8, 6, 5, 2, 1, 7, 3, 4]
		[9, 6, 9, 6, 9, 6] → [6, 6, 9, 9, 9, 6]
		[5, 8, 5, 8, 3, 6, 1] → [5, 5, 8, 8, 3, 6, 1]
		[0, 4, 1, 9, 7] → [0, 1, 4, 9, 7]
0.186	c201	*elements ¿ element 1, followed by elements ¡ element 1*
	28	`(λ (x) (concat (filter (λ (y) (< (first x) y)) x) (filter (λ (z) (> (first x) z)) x)))`
		[9, 0, 73, 25, 4] → [73, 25, 0, 4]
		[57, 62, 34, 54, 3, 6, 75, 8, 91, 99] → [62, 75, 91, 99, 34, 54, 3, 6, 8]
		[51, 20, 95, 39, 52, 53, 78, 2] → [95, 52, 53, 78, 20, 39, 2]
		[40] → []
		[43, 41, 22, 48, 77, 82, 18] → [48, 77, 82, 41, 22, 18]
0.182	c217	*elements in ascending order by ones digits*
	11	`(λ (x) (sort (λ (y) (% y 10)) x))`
		[34, 33, 80, 4, 79] → [80, 33, 34, 4, 79]
		[36, 72, 45, 67, 50, 90] → [50, 90, 72, 45, 36, 67]
		[66, 52, 15, 32] → [52, 32, 15, 66]
		[83, 30, 28, 38, 21, 0, 5] → [30, 0, 21, 83, 5, 28, 38]
		[63, 42, 1, 69, 61, 75, 46] → [1, 61, 42, 63, 75, 46, 69]
0.178	c005	*remove all but element N + 1, N = element 1*
	14	`(λ (x) (singleton (nth (first x) (drop 1 x))))`
	44	`(λ (x) (fix x (λ (y z) (if (is_equal 1 (head z)) (cons (head (tail z)) empty) (y (cons (- (head z) 1) (tail (tail z))))))))`
		[2, 1, 9, 6, 7, 0, 4, 5, 3] → [9]
		[7, 2, 1, 8, 0, 6, 3, 5, 9, 4] → [5]
		[5, 1, 7, 6, 9, 8, 2, 0, 3, 4] → [8]
		[9, 1, 6, 4, 7, 5, 3, 8, 2, 0] → [0]
		[4, 1, 9, 6, 3, 2, 5, 0, 8, 7] → [3]
0.178	c209	*elements in ascending order; insert sum of smallest and largest elements at index 3*
	21	`(λ (x) (insert (+ (max x) (min x)) 3 (sort (λ (y) y) x)))`
		[34, 2, 3, 96, 64] → [2, 3, 98, 34, 64, 96]
		[87, 76, 1, 38, 85, 83] → [1, 38, 88, 76, 83, 85, 87]
		[9, 67, 94, 5] → [5, 9, 99, 67, 94]
		[39, 86, 23, 8, 7, 31] → [7, 8, 93, 23, 31, 39, 86]
		[25, 72, 49] → [25, 49, 97, 72]
0.174	c063	*remove the first N + 1 elements, N = element 1*
	12	`(λ (x) (drop (first x) (drop 1 x)))`
	38	`(λ (x) (fix x (λ (y z) (if (is_equal 0 (head z)) (tail z) (y (cons (- (head z) 1) (tail (tail z))))))))`
		[2, 6, 2, 6, 6, 6] → [6, 6, 6]
		[4, 1, 1, 4, 1, 1, 3] → [1, 3]
		[3, 3, 3, 3, 3, 3, 3, 3, 3, 3] → [3, 3, 3, 3, 3, 3]
		[1, 7, 9, 9, 8, 4, 1, 7, 8] → [9, 9, 8, 4, 1, 7, 8]
		[0, 3, 9, 4, 6, 6, 7, 8, 2] → [3, 9, 4, 6, 6, 7, 8, 2]

μ	ID/$\mathcal{L}$	Description, Program, & Examples
0.161	c141	*replace element M + 2 with N, M = element 1, N = element 2; remove elements 1 and 2*
	16	`(λ (x) (replace (first x) (second x) (drop 2 x)))`
		[4, 3, 12, 6, 67, 1, 9] → [12, 6, 67, 3, 9]<br>[3, 1, 2, 10, 90, 6, 0, 76] → [2, 10, 1, 6, 0, 76]<br>[5, 7, 33, 0, 71, 9, 78, 4, 2, 66] → [33, 0, 71, 9, 7, 4, 2, 66]<br>[1, 0, 1, 39, 49, 14, 90, 57, 0, 99] → [0, 39, 49, 14, 90, 57, 0, 99]<br>[2, 4, 59, 62, 5, 6, 36, 45, 64] → [59, 4, 5, 6, 36, 45, 64]
0.155	c089	*swap elements 2 and 3 if element 2 ¿ element 3, else swap elements 1 and 4*
	28	`(λ (x) (if (> (second x) (third x)) (swap 2 3 x) (swap 1 4 x)))`
	96	`(λ (x) (if (> (head (tail x)) (head (tail (tail x)))) (cons (head x) (cons (head (tail (tail x))) (cons (head (tail x)) (tail (tail (tail x)))))) (cons (head (tail (tail (tail x)))) (cons (head (tail x)) (cons (head (tail (tail x))) (cons (head x) (tail (tail (tail (tail x))))))))))`
		[90, 7, 14, 59, 53, 24, 25] → [59, 7, 14, 90, 53, 24, 25]<br>[28, 99, 6, 43, 41, 28] → [28, 6, 99, 43, 41, 28]<br>[96, 8, 51, 44, 20] → [44, 8, 51, 96, 20]<br>[58, 65, 32, 63] → [58, 32, 65, 63]<br>[78, 97, 77, 93, 64, 3, 38, 18, 0, 24] → [78, 77, 97, 93, 64, 3, 38, 18, 0, 24]
0.148	c124	*element M, M = element N, N = element 1*
	14	`(λ (x) (singleton (nth (nth (first x) x) x)))`
		[2, 5, 0, 82, 9] → [9]<br>[4, 93, 98, 3, 1, 96] → [98]<br>[3, 27, 5, 2, 8, 7, 97, 84, 42] → [8]<br>[8, 25, 16, 35, 6, 1, 49, 3, 2, 55] → [16]<br>[7, 61, 0, 56, 8, 92, 4, 2] → [56]
0.141	c216	*elements in ascending order by tens digits*
	11	`(λ (x) (sort (λ (y) (/ y 10)) x))`
		[77, 74, 26, 9, 31] → [9, 26, 31, 77, 74]<br>[54, 86, 4, 66, 25, 13, 84] → [4, 13, 25, 54, 66, 86, 84]<br>[91, 20, 3, 82] → [3, 20, 82, 91]<br>[62, 32, 78, 53, 42, 8, 6] → [8, 6, 32, 42, 53, 62, 78]<br>[22, 38, 58, 30, 92, 14] → [14, 22, 38, 30, 58, 92]
0.14	c143	*replace every occurrence of the largest element with the smallest element*
	21	`(λ (x) (map (λ (y) (if (== y (max x)) (min x) y)) x))`
		[9, 0, 49, 49, 3] → [9, 0, 0, 0, 3]<br>[8, 82, 71, 25, 24, 31, 90, 34, 69, 6] → [8, 82, 71, 25, 24, 31, 6, 34, 69, 6]<br>[9, 83, 0, 56, 18, 48, 61, 5, 12] → [9, 0, 0, 56, 18, 48, 61, 5, 12]<br>[74, 8, 2, 74, 22, 4] → [2, 8, 2, 2, 22, 4]<br>[52, 44, 16, 1, 6, 5, 52, 23] → [1, 44, 16, 1, 6, 5, 1, 23]
0.139	c215	*replace element M + 1 with the length of the input, M = element 1; drop element 1*
	16	`(λ (x) (replace (first x) (length x) (drop 1 x)))`
		[3, 59, 55, 17, 3] → [59, 55, 5, 3]<br>[8, 4, 0, 43, 95, 70, 1, 34, 0, 4] → [4, 0, 43, 95, 70, 1, 34, 10, 4]<br>[5, 74, 35, 35, 36, 78, 36, 35] → [74, 35, 35, 36, 8, 36, 35]<br>[5, 31, 60, 84, 7, 89, 96] → [31, 60, 84, 7, 7, 96]<br>[6, 25, 8, 72, 79, 27, 24, 2, 94] → [25, 8, 72, 79, 27, 9, 2, 94]

μ	ID/ℒ	Description, Program, & Examples
0.136	c144	*replace every occurrence of the largest or smallest element with their absolute difference*
	37	`(λ (x) (map (λ (y) (if (or (== y (max x)) (== y (min x))) (- (max x) (min x)) y)) x))`
		[60, 87, 71, 94, 35] → [60, 87, 71, 59, 59]<br>[11, 90, 4, 16] → [11, 86, 86, 16]<br>[14, 52, 5] → [14, 47, 47]<br>[23, 77] → [54, 54]<br>[22] → [0]
0.129	c128	*elements in ascending order after removing elements 1, 2, and 5*
	15	`(λ (x) (sort (λ (y) y) (cut_idx 3 (drop 2 x))))`
		[14, 46, 41, 44, 85, 5] → [5, 41, 44]<br>[34, 74, 96, 40, 9, 7, 58, 2] → [2, 7, 40, 58, 96]<br>[69, 8, 38, 91, 83, 54, 10, 17, 51] → [10, 17, 38, 51, 54, 91]<br>[3, 1, 28, 0, 6, 93, 90, 9, 56, 45] → [0, 9, 28, 45, 56, 90, 93]<br>[26, 49, 8, 4, 21, 16, 63] → [4, 8, 16, 63]
0.127	c208	*keep only the last N elements, where N is the last element*
	8	`(λ (x) (takelast (last x) x))`
		[3, 1, 5, 35, 65, 7, 84, 4] → [65, 7, 84, 4]<br>[28, 69, 5, 58, 51, 66, 16, 9, 7] → [5, 58, 51, 66, 16, 9, 7]<br>[23, 9, 22, 0, 76, 3, 5, 75, 2, 1] → [1]<br>[2, 24, 92, 14, 95, 54, 79, 42, 71, 8] → [92, 14, 95, 54, 79, 42, 71, 8]<br>[94, 8, 68, 86, 7, 95, 56, 0, 6] → [86, 7, 95, 56, 0, 6]
0.124	c035	*swap elements 1 and 4 if element 2 = element 3, else swap elements 2 and 3*
	28	`(λ (x) (if (== (second x) (third x)) (swap 1 4 x) (swap 2 3 x)))`
	96	`(λ (x) (if (is_equal (head (tail x)) (head (tail (tail x)))) (cons (head (tail (tail (tail x)))) (cons (head (tail x)) (cons (head (tail (tail x))) (cons (head x) (tail (tail (tail (tail x)))))))) (cons (head x) (cons (head (tail (tail x))) (cons (head (tail x)) (tail (tail (tail x))))))))`
		[0, 5, 0, 0] → [0, 0, 5, 0]<br>[7, 1, 7, 7, 1, 7, 1] → [7, 7, 1, 7, 1, 7, 1]<br>[3, 3, 3, 2, 2, 6] → [2, 3, 3, 3, 2, 6]<br>[9, 9, 8, 9, 8] → [9, 8, 9, 9, 8]<br>[5, 5, 4, 5, 6, 6, 4, 4, 4, 6] → [5, 4, 5, 5, 6, 6, 4, 4, 4, 6]
0.118	c214	*remove elements 1 and N + 1, where N is element 1*
	12	`(λ (x) (cut_idx (first x) (drop 1 x)))`
		[4, 47, 54, 54, 4, 4] → [47, 54, 54, 4]<br>[7, 6, 30, 64, 8, 41, 41, 36, 56] → [6, 30, 64, 8, 41, 41, 56]<br>[2, 7, 23, 63, 63, 23, 93, 78] → [7, 63, 63, 23, 93, 78]<br>[3, 3, 21, 32, 21, 21, 98] → [3, 21, 21, 21, 98]<br>[9, 27, 19, 1, 64, 61, 61, 67, 27, 65] → [27, 19, 1, 64, 61, 61, 67, 27]
0.116	c130	*elements 2 through N + 1, N = element 1*
	12	`(λ (x) (take (first x) (drop 1 x)))`
		[5, 0, 24, 4, 41, 18, 9] → [0, 24, 4, 41, 18]<br>[1, 75, 48, 90, 5, 9, 4, 21, 59] → [75]<br>[0, 96, 6, 62, 83, 96, 43, 25, 52, 43] → []<br>[3, 81, 0, 8, 87, 62, 6, 10] → [81, 0, 8]<br>[7, 9, 6, 4, 15, 45, 88, 83, 26, 92] → [9, 6, 4, 15, 45, 88, 83]

μ	ID/$\mathcal{L}$	Description, Program, & Examples
0.115	c159	*element N counts the number of occurrences of N, up to the largest element*
	24	`(λ (x) (map (λ (y) (count (λ (z) (== y z)) x)) (range 1 1 (max x))))`
		[8, 8, 8, 6, 5, 5, 5, 6] → [0, 0, 0, 0, 3, 2, 0, 3]<br>[3, 4, 3, 5, 4, 4, 5, 5, 4, 3] → [0, 0, 3, 4, 3]<br>[7, 6, 6, 6, 6, 2, 2, 7, 6] → [0, 2, 0, 0, 0, 5, 2]<br>[4, 4, 7, 7, 9, 4, 9, 2, 2] → [0, 2, 0, 3, 0, 0, 2, 0, 2]<br>[1, 1, 1, 1, 1, 1, 1, 1, 1, 1] → [10]
0.114	c205	*product of elements divisible by 4*
	19	`(λ (x) (singleton (product (filter (λ (y) (== (% y 4) 0)) x))))`
		[4, 1, 9, 20, 3, 11, 70] → [80]<br>[99, 46, 7, 5, 2, 8, 5, 12] → [96]<br>[15, 73, 98, 8, 53, 1, 95, 9, 4] → [32]<br>[67, 19, 23, 7, 2, 12, 8, 23, 97, 6] → [96]<br>[83, 37, 16, 21, 0, 6, 2, 87, 7] → [0]
0.112	c076	*the maximum, last element, length, first element, and minimum, in that order*
	30	`(λ (x) (cons (max x) (cons (last x) (cons (length x) (cons (first x) (singleton (min x)))))))`
	172	`(λ (x) (cons (fix (cons 0 x) (λ (y z) (if (is_empty (tail z)) (head z) (y (if (> (head z) (head (tail z))) (cons (head z) (tail (tail z))) (tail z)))))) (cons (fix x (λ (u v) (if (is_empty (tail v)) (head v) (u (tail v))))) (cons (fix x (λ (w a) (if (is_empty a) 0 (+ 1 (w (tail a)))))) (cons (head x) (cons (fix (cons 9 x) (λ (b c) (if (is_empty (tail c)) (head c) (b (if (> (head c) (head (tail c))) (tail c) (cons (head c) (tail (tail c)))))))) empty))))))`
		[5, 7, 9, 4] → [9, 4, 4, 5, 4]<br>[0, 6, 1, 9, 7, 8, 4, 2, 5, 3] → [9, 3, 10, 0, 0]<br>[6] → [6, 6, 1, 6, 6]<br>[8, 2] → [8, 2, 2, 8, 2]<br>[3, 5, 4, 0, 8, 7, 1] → [8, 1, 7, 3, 0]
0.107	c250	*use 0s to delimit sublists; concatenate reverse of each sublist*
	48	`(λ (x) (flatten (map reverse (reverse (fold (λ (y z) (if (== z 0) (cons empty y) (cons (append (first y) z) (drop 1 y)))) (singleton empty) x)))))`
		[38, 0, 38, 3, 60, 60, 0, 3, 38] → [38, 60, 60, 3, 38, 38, 3]<br>[4, 8, 0, 77, 0, 25, 66, 77, 40, 66] → [8, 4, 77, 66, 40, 77, 66, 25]<br>[7, 27, 0, 8, 5, 0, 86, 24, 4, 4] → [27, 7, 5, 8, 4, 4, 24, 86]<br>[6, 14, 75, 0, 1, 75, 75, 0, 0] → [75, 14, 6, 75, 75, 1]<br>[2, 0, 62, 80, 54, 49, 0, 6, 4] → [2, 49, 54, 80, 62, 4, 6]
0.103	c186	*keep only elements greater than element 1*
	13	`(λ (x) (filter (λ (y) (> y (first x))) x))`
		[6, 71, 97, 98, 0, 64, 60, 3, 0] → [71, 97, 98, 64, 60]<br>[79, 7, 32, 2, 8, 86, 93, 51, 95, 5] → [86, 93, 95]<br>[31, 30, 53, 74, 9, 12, 12, 5, 31, 31] → [53, 74]<br>[77, 20, 26, 99, 12, 81, 27, 90, 90, 77] → [99, 81, 90, 90]<br>[72, 17, 4, 50, 80, 17, 37, 29, 57] → [80]
0.0979	c242	*take the largest unique element, append the second largest unique element, prepend the third largest unique element, append the fourth largest unique element, and so on*
	25	`(λ (x) (fold (λ (y z) (append (reverse y) z)) empty (reverse (unique (sort (λ (u) u) x)))))`
		[43, 43, 43, 17, 17, 4, 17, 17] → [17, 43, 4]<br>[32, 81, 53, 32, 1, 32, 81, 53, 81, 1] → [32, 81, 53, 1]<br>[66, 0, 6, 66, 31, 66, 28, 28] → [6, 31, 66, 28, 0]<br>[60, 92, 9, 18, 35, 9, 10, 60, 10, 92] → [10, 35, 92, 60, 18, 9]<br>[51, 46, 74, 46, 75, 48, 89, 89, 51] → [48, 74, 89, 75, 51, 46]

μ	ID/$\mathcal{L}$	Description, Program, & Examples
0.0909	c160	*replace each element, M, with 99 - M*
	11	`(λ (x) (map (λ (y) (- 99 y)) x))`
		[55, 82, 57, 4, 59, 6] → [44, 17, 42, 95, 40, 93]
		[1, 13, 34, 6, 36, 79, 21, 11, 64, 46] → [98, 86, 65, 93, 63, 20, 78, 88, 35, 53]
		[32, 70, 51, 41, 5, 69, 28] → [67, 29, 48, 58, 94, 30, 71]
		[9, 72, 7, 75, 74, 1, 79, 84, 2] → [90, 27, 92, 24, 25, 98, 20, 15, 97]
		[8, 66, 3, 43, 2, 73, 48, 74] → [91, 33, 96, 56, 97, 26, 51, 25]
0.0727	c167	*keep only elements divisible by 3*
	15	`(λ (x) (filter (λ (y) (== (% y 3) 0)) x))`
		[18, 78, 3, 5, 62, 8] → [18, 78, 3]
		[27, 70, 0, 21, 74, 33, 87, 12, 22] → [27, 0, 21, 33, 87, 12]
		[31, 91, 84, 30, 38, 10, 66, 0, 98, 94] → [84, 30, 66, 0]
		[90, 50, 3, 1, 99, 0, 93] → [90, 3, 99, 0, 93]
		[45, 8, 2, 69, 39, 9, 44, 4] → [45, 69, 39, 9]
0.0682	c202	*the indices of every even number*
	6	`(λ (x) (find is_even x))`
		[31, 98, 55, 14, 50] → [2, 4, 5]
		[71, 7, 23, 20, 13, 66, 26, 42, 52] → [4, 6, 7, 8, 9]
		[91, 78, 5, 46, 15, 33, 0, 62] → [2, 4, 7, 8]
		[73, 41, 58, 24] → [3, 4]
		[90, 60, 25] → [1, 2]
0.0556	c129	*elements M + 2 through N + 2, M = element 1, N = element 2*
	16	`(λ (x) (slice (first x) (second x) (drop 2 x)))`
		[2, 3, 75, 17, 8, 6] → [17, 8]
		[1, 4, 99, 5, 4, 14, 73, 28] → [99, 5, 4, 14]
		[4, 6, 37, 8, 1, 31, 7, 69, 62, 67] → [31, 7, 69]
		[4, 5, 20, 66, 61, 19, 16] → [19, 16]
		[6, 6, 70, 27, 86, 99, 7, 30, 66] → [30]
0.0481	c221	*sum of even elements*
	10	`(λ (x) (singleton (sum (filter is_even x))))`
		[46, 91, 4, 9, 1, 67, 5] → [50]
		[65, 44, 3, 1, 91, 7, 41, 43, 20] → [64]
		[55, 26, 34, 95, 19, 6, 0, 79, 8, 53] → [74]
		[81, 21, 0, 32, 16, 4, 2, 28, 33] → [82]
		[6, 13, 7, 10, 47, 75, 80, 93] → [96]
0.0455	c131	*keep only elements whose tens digit is even*
	13	`(λ (x) (filter (λ (y) (is_even (/ y 10))) x))`
		[68, 93, 10, 24, 5] → [68, 24, 5]
		[54, 99, 84, 58] → [84]
		[3, 38, 7, 16, 78, 51, 41, 12, 67, 77] → [3, 7, 41, 67]
		[95, 37, 73, 97, 97, 56, 97, 97] → []
		[94, 0, 9, 5, 6, 33, 4, 59, 54] → [0, 9, 5, 6, 4]
0.0455	c180	*take the smallest element, append the second smallest, prepend the third smallest, append the fourth smallest, and so on*
	21	`(λ (x) (fold (λ (y z) (append (reverse y) z)) empty (sort (λ (u) u) x)))`
		[43, 6, 18, 33, 8, 38] → [38, 18, 6, 8, 33, 43]
		[11, 7, 56, 84, 4, 60, 5] → [60, 11, 5, 4, 7, 56, 84]
		[1, 67, 23, 63, 59, 36, 45, 21, 5] → [63, 45, 23, 5, 1, 21, 36, 59, 67]
		[93, 3, 48, 34, 21, 83, 14, 66, 65, 80] → [83, 66, 48, 21, 3, 14, 34, 65, 80, 93]
		[92, 94, 57, 2, 98, 89, 13, 28] → [94, 89, 28, 2, 13, 57, 92, 98]

μ	ID/$\mathcal{L}$	Description, Program, & Examples
0.042	c015	*elements M + 2 through N + 2, M = element 1, N = element 2*
	16	`(λ (x) (slice (first x) (second x) (drop 2 x)))`
	88	`(λ (x) (fix (cons (head x) (fix (tail x) (λ (y z) (if (is_equal 0 (head z)) empty (cons (head (tail z)) (y (cons (- (head z) 1) (tail (tail z))))))))) (λ (u v) (if (is_equal 1 (head v)) (tail v) (u (cons (- (head v) 1) (tail (tail v))))))))`
		[3, 4, 5, 5, 4, 3, 3] → [4, 3]<br>[1, 3, 9, 2, 0, 5, 7, 5, 7, 1] → [9, 2, 0]<br>[4, 5, 6, 6, 4, 6, 9, 9] → [6, 9]<br>[1, 1, 3, 2, 3, 4, 5, 6, 5, 1] → [3]<br>[1, 5, 8, 3, 6, 2, 4, 8, 0] → [8, 3, 6, 2, 4]
0.0404	c179	*take the largest element, append the second largest, prepend the third largest, append the fourth largest, and so on*
	23	`(λ (x) (fold (λ (y z) (append (reverse y) z)) empty (reverse (sort (λ (u) u) x))))`
		[80, 68, 56, 38, 97, 92] → [56, 80, 97, 92, 68, 38]<br>[3, 81, 25, 8, 5, 7, 41, 75, 39] → [5, 8, 39, 75, 81, 41, 25, 7, 3]<br>[46, 85, 95, 0, 38, 4, 66, 35] → [4, 38, 66, 95, 85, 46, 35, 0]<br>[53, 27, 30, 13, 1, 18, 55] → [13, 27, 53, 55, 30, 18, 1]<br>[11, 4, 36, 71, 19, 2, 90, 6, 10, 86] → [4, 10, 19, 71, 90, 86, 36, 11, 6, 2]
0.0395	c226	*remove first M and last N elements, M = element 1, N = last element*
	14	`(λ (x) (drop (first x) (droplast (last x) x)))`
		[1, 15, 15, 1, 15, 1, 1] → [15, 15, 1, 15, 1]<br>[6, 67, 67, 67, 6, 67, 1, 1] → [1]<br>[5, 64, 64, 5, 83, 83, 83, 83, 8, 5] → []<br>[1, 14, 61, 49, 2, 12, 98, 4, 4] → [14, 61, 49, 2]<br>[1, 5, 3, 96, 37, 35, 68, 5, 23, 0] → [5, 3, 96, 37, 35, 68, 5, 23, 0]
0.0341	c134	*remove elements M through N, M = element 1, N = element 2*
	12	`(λ (x) (cut_slice (first x) (second x) x))`
		[3, 4, 9, 6, 91] → [3, 4, 91]<br>[2, 9, 3, 29, 19, 61, 23, 59, 66, 76] → [2, 76]<br>[3, 5, 31, 85, 37, 9, 4] → [3, 5, 9, 4]<br>[1, 8, 0, 65, 95, 28, 3, 7] → []<br>[2, 4, 0, 7] → [2]
0.0303	c164	*replace each element, M, with M / 4 + 5*
	15	`(λ (x) (map (λ (y) (+ (/ y 4) 5)) x))`
		[22, 14, 26, 39, 26, 13] → [10, 8, 11, 14, 11, 8]<br>[6, 50, 18, 72, 7, 84, 94, 0, 46, 8] → [6, 17, 9, 23, 6, 26, 28, 5, 16, 7]<br>[31] → [12]<br>[55, 3, 92, 85, 63, 58, 33, 67, 48] → [18, 5, 28, 26, 20, 19, 13, 21, 17]<br>[8, 2, 42, 59, 95, 97, 3] → [7, 5, 15, 19, 28, 29, 5]
0.0202	c178	*keep only elements followed by an even number*
	27	`(λ (x) (map first (filter (λ (y) (is_even (second y))) (zip (droplast 1 x) (drop 1 x)))))`
		[27, 4, 9, 71, 45, 69] → [27]<br>[10, 68, 80, 5, 29, 23, 9, 33, 69] → [10, 68]<br>[73, 32, 70, 0, 22, 2, 46, 8, 7, 92] → [73, 32, 70, 0, 22, 2, 46, 7]<br>[19, 81, 1, 53, 85, 3, 97] → []<br>[40, 2, 91, 28, 61, 0, 55, 4] → [40, 91, 61, 55]
0.0182	c113	*keep only elements whose ones digit is greater than element 1*
	17	`(λ (x) (filter (λ (y) (> (first x) (% y 10))) x))`
		[3, 91, 59, 91, 60, 6, 44, 2] → [91, 91, 60, 2]<br>[7, 35, 37, 74, 73, 22, 85, 8, 68, 7] → [35, 74, 73, 22, 85]<br>[7, 38, 1, 29, 40, 48, 45, 81] → [1, 40, 45, 81]<br>[2, 93, 68, 36, 41, 8, 27, 20, 8, 50] → [41, 20, 50]<br>[4, 82, 5, 52, 83, 7, 5, 4, 9] → [82, 52, 83]

μ	ID/$\mathcal{L}$	Description, Program, & Examples
0.013	c206	*keep only elements whose value is between the first two elements*
	31	`(λ (x) (filter (λ (y) (and (> (max (take 2 x)) y) (> y (min (take 2 x))))) x))`
		[43, 3, 80, 40, 29, 31] → [40, 29, 31]<br>[1, 63, 2, 7, 48, 9, 97, 4] → [2, 7, 48, 9, 4]<br>[39, 80, 95, 9, 44, 77, 2, 33, 75, 6] → [44, 77, 75]<br>[6, 90, 25, 9, 18, 0, 7] → [25, 9, 18, 7]<br>[87, 5, 71, 7, 3, 19, 8, 22, 56] → [71, 7, 19, 8, 22, 56]
0.0101	c210	*unique elements with last element inserted at index M, where M is element 1*
	14	`(λ (x) (insert (last x) (first x) (unique x)))`
		[2, 9, 57, 6, 9, 6] → [2, 6, 9, 57, 6]<br>[4, 0, 50, 4, 19, 34, 50, 34, 4, 19] → [4, 0, 50, 19, 19, 34]<br>[8, 79, 23, 60, 74, 49, 71, 0, 76] → [8, 79, 23, 60, 74, 49, 71, 76, 0, 76]<br>[2, 99, 87, 2, 87, 99, 87] → [2, 87, 99, 87]<br>[7, 65, 3, 68, 73, 66, 9, 5] → [7, 65, 3, 68, 73, 66, 5, 9, 5]
0.0	c158	*replace each element with 1 if element N equals N, else 0*
	18	`(λ (x) (mapi (λ (y z) (if (== z y) 1 0)) x))`
		[1, 45, 3, 4, 23, 55] → [1, 0, 1, 1, 0, 0]<br>[4, 68, 3, 68, 24, 8, 7, 8] → [0, 0, 1, 0, 0, 0, 1, 1]<br>[84, 2, 29, 7, 35, 2, 4, 8, 9, 31] → [0, 1, 0, 0, 0, 0, 0, 1, 1, 0]<br>[9, 3, 8, 6, 7, 5, 2] → [0, 0, 0, 0, 0, 0, 0]<br>[16, 20, 67, 4, 63, 1, 7, 6, 9, 4] → [0, 0, 0, 1, 0, 0, 1, 0, 1, 0]
0.0	c183	*list the index minus 1 of elements 2 and following equal to element 1*
	14	`(λ (x) (find (== (first x)) (drop 1 x)))`
		[3, 4, 3, 3, 3, 9, 5, 8] → [2, 3, 4]<br>[8, 7, 2, 1, 1, 2, 8, 9, 6] → [6]<br>[2, 2, 2, 2, 5, 2, 5, 4, 4, 5] → [1, 2, 3, 5]<br>[6, 3, 6, 6, 9, 9, 3, 6, 6, 6] → [2, 3, 7, 8, 9]<br>[2, 12, 2, 49, 8, 2, 65, 83, 36] → [2, 5]

Supplementary References

1. *Term rewriting systems* (eds Bezem, M., Klop, J. W. & de Vrijer, R.) (Cambridge University Press, 2003).
2. Baader, F. & Nipkow, T. *Term Rewriting And All That* (Cambridge University Press, 1999).
3. Robinson, J. A. A machine-oriented logic based on the resolution principle. *Journal of the ACM (JACM)* **12,** 23–41 (1965).
4. Martelli, A. & Montanari, U. An efficient unification algorithm. *ACM Transactions on Programming Languages and Systems (TOPLAS)* **4,** 258–282 (1982).
5. Baader, F. & Snyder, W. in *Handbook of automated reasoning* 447–533 (Gulf Professional, 2001).
6. Hwang, I., Stuhlmüller, A. & Goodman, N. D. Inducing probabilistic programs by Bayesian program merging. *arXiv preprint arXiv:1110.5667* (2011).
7. Cao, D. *et al.* babble: Learning Better Abstractions with E-Graphs and Anti-Unification. *Proceedings of the ACM on Programming Languages* **7,** 396–424 (2023).
8. Plotkin, G. D. A note on inductive generalization. *Machine intelligence* **5,** 153–163 (1970).
9. Plotkin, G. D. A further note on inductive generalization. *Machine intelligence* **6,** 101–124 (1971).
10. Fowler, M. *flat-map* (Accessed 2023-10-24). https://martinfowler.com/articles/collection-pipeline/flat-map.html.
11. Of Glasgow, T. U. *Data.List* (Accessed 2023-10-24). https://hackage.haskell.org/package/base-4.19.0.0/docs/Data-List.html#v:concatMap.
12. Contributors, M. *Array.Prototype.flatMap* (Accessed 2023-10-24). https://developer.mozilla.org/en-US/docs/Web/JavaScript/Reference/Global_Objects/Array/flatMap.
13. Britt, J. *module Enumerable - RDoc Documentation* (Accessed 2023-10-24). https://ruby-doc.org/3.2.2/Enumerable.html#method-i-flat_map.
14. Kim, Z. *mapcat - clojure.core — ClojureDocs - Community-Powered Clojure Documentation and Examples* (Accessed 2023-10-24). https://clojuredocs.org/clojure.core/mapcat.
15. Douglas, B. D., Ewell, P. J. & Brauer, M. Data quality in online human-subjects research: Comparisons between MTurk, Prolific, CloudResearch, Qualtrics, and SONA. *Plos one* **18,** e0279720 (2023).
