# Supplementary material for: Symbolic metaprogram search improves learning efficiency and explains rule learning in humans
Source: Nat Commun. 2024 Aug 10;15:6847. doi: 10.1038/s41467-024-50966-x (PMC11316799; doi:10.1038/s41467-024-50966-x)
Supplement: Supplementary file 3 — Reporting Summary [file 41467_2024_50966_MOESM3_ESM.pdf]

nature portfolio

Corresponding author(s): Joshua S. Rule

Last updated by author(s): Jul 17, 2024

Reporting Summary

Nature Portfolio wishes to improve the reproducibility of the work that we publish. This form provides structure for consistency and transparency in reporting. For further information on Nature Portfolio policies, see our Editorial Policies and the Editorial Policy Checklist.

Statistics

For all statistical analyses, confirm that the following items are present in the figure legend, table legend, main text, or Methods section.

n/a	Confirmed	
☐	☒	The exact sample size (*n*) for each experimental group/condition, given as a discrete number and unit of measurement
☐	☒	A statement on whether measurements were taken from distinct samples or whether the same sample was measured repeatedly
☐	☒	The statistical test(s) used AND whether they are one- or two-sided<br>*Only common tests should be described solely by name; describe more complex techniques in the Methods section.*
☐	☒	A description of all covariates tested
☐	☒	A description of any assumptions or corrections, such as tests of normality and adjustment for multiple comparisons
☐	☒	A full description of the statistical parameters including central tendency (e.g. means) or other basic estimates (e.g. regression coefficient) AND variation (e.g. standard deviation) or associated estimates of uncertainty (e.g. confidence intervals)
☐	☒	For null hypothesis testing, the test statistic (e.g. *F*, *t*, *r*) with confidence intervals, effect sizes, degrees of freedom and *P* value noted<br>*Give P values as exact values whenever suitable.*
☐	☒	For Bayesian analysis, information on the choice of priors and Markov chain Monte Carlo settings
☒	☐	For hierarchical and complex designs, identification of the appropriate level for tests and full reporting of outcomes
☐	☒	Estimates of effect sizes (e.g. Cohen's *d*, Pearson's *r*), indicating how they were calculated

Our web collection on statistics for biologists contains articles on many of the points above.

Software and code

Policy information about availability of computer code

Data collection	Model data collection relied on several models previously reported in the literature (Enumerate, Fleet, Metagol, RobustFill, Codex) and one novel model (HL). Code to download and run this novel model is available at https://doi.org/10.17605/OSF.IO/GQ2HJ. Human behavioral data collection relied on an experimental paradigm available at https://github.com/joshrule/list-routines-human-experiments.
Data analysis	Data analysis relied on custom code available at https://doi.org/10.17605/OSF.IO/GQ2HJ.

For manuscripts utilizing custom algorithms or software that are central to the research but not yet described in published literature, software must be made available to editors and reviewers. We strongly encourage code deposition in a community repository (e.g. GitHub). See the Nature Portfolio guidelines for submitting code & software for further information.

Data

Policy information about availability of data

All manuscripts must include a data availability statement. This statement should provide the following information, where applicable:

- Accession codes, unique identifiers, or web links for publicly available datasets
- A description of any restrictions on data availability
- For clinical datasets or third party data, please ensure that the statement adheres to our policy

All data are publicly available at https://doi.org/10.17605/OSF.IO/GQ2HJ.

Human research participants

Policy information about studies involving human research participants and Sex and Gender in Research.

Reporting on sex and gender	Our analysis sample included 392 participants: 253 self-reported as male, 132 self-reported as female, 2 self-reported as other genders, and 5 did not respond. Neither sex nor gender were included in the study design and did not figure into our analysis beyond identifying the makeup of our subject pool.
Population characteristics	See below.
Recruitment	Participants were recruited using the Amazon Mechanical Turk platform. We required that users complete the experiment on a laptop or desktop computer (i.e. phones and tablets were excluded), be located in the United States, be capable of understanding English text, and have successfully completed at least 95% of their prior assignments on Amazon Mechanical Turk. Participants self-selected the experiment based on keywords and a brief description of the task.
Ethics oversight	The Institutional Review Board at Massachusetts Institute of Technology approved the study.

Note that full information on the approval of the study protocol must also be provided in the manuscript.

Field-specific reporting

Please select the one below that is the best fit for your research. If you are not sure, read the appropriate sections before making your selection.

☐ Life sciences ☒ Behavioural & social sciences ☐ Ecological, evolutionary & environmental sciences

For a reference copy of the document with all sections, see nature.com/documents/nr-reporting-summary-flat.pdf

Behavioural & social sciences study design

All studies must disclose on these points even when the disclosure is negative.

Study description	We report a quantitative experimental study involving human participants and computational models of human learning.
Research sample	498 people participated in the experiment. 106 were excluded (see below), and we analyzed data from the remaining 392. Participant age for this group ranged from 18.6yrs to 69.4yrs (median: 39.2 yrs), with 253 self-reporting as male, 132 self-reporting as female, 2 self-reporting as other genders, and 5 not self-reporting. See "Recruitment" above for more details on the sample. Using Amazon Mechanical Turk platform allowed us to investigate a much larger sample than would have been possible otherwise.
Sampling strategy	We recruited using convenience sampling. We publicly posted our experiment on the Amazon Mechanical Turk platform and included those participants who chose to complete the experiment. Sample size was chosen such that we would be able to test ~20 people per concept in our 250 concept pool, consistent with prior large-scale behavioral investigations of concept learning (e.g. Piantadosi, Goodman, Tenenbaum, 2016).
Data collection	Participants generated data independently via computer using the Amazon Mechanical Turk platform. The experimenter did not interact with the participant or the data until after completion of the experiment.
Timing	Behavioral data was collected between 2020-07-08 and 2020-07-15. Each participant completed the experiment independently in a single session.
Data exclusions	After reviewing pilot data, we pre-established three exclusion criteria. Participants were excluded for completing the experiment: 1) in less than 20min; 2) with fewer than 10 correct responses; or 3) with more than 20 identical responses. These criteria were designed to remove participants who did not appear to make a good faith effort to complete the experiment and were primarily motivated by the financial compensation. Using these criteria, we excluded 106 of our original 498 participants, analyzing data from the remaining 392.
Non-participation	779 participants began our experiment, 498 completed the experiment and were compensated, and 281 did not complete the experiment either due to technical errors or voluntarily leaving the experiment before completion. Those experiencing technical difficulties were compensated for their participation. The practice of beginning and then abandoning an assignment (known on Amazon Mechanical Turk as "returning the HIT") is common, particularly for challenging tasks like the paradigm we used in our experiment.
Randomization	Each participant completed 10 blocks of trials. Each block was associated with a different concept. Concepts were randomly assigned to participants.

Reporting for specific materials, systems and methods

We require information from authors about some types of materials, experimental systems and methods used in many studies. Here, indicate whether each material, system or method listed is relevant to your study. If you are not sure if a list item applies to your research, read the appropriate section before selecting a response.

Materials & experimental systems

n/a	Involved in the study	
☒	☐	Antibodies
☒	☐	Eukaryotic cell lines
☒	☐	Palaeontology and archaeology
☒	☐	Animals and other organisms
☒	☐	Clinical data
☒	☐	Dual use research of concern

Methods

n/a	Involved in the study	
☒	☐	ChIP-seq
☒	☐	Flow cytometry
☒	☐	MRI-based neuroimaging
